# Esters of terpene alcohols as highly potent, reversible, and low toxic skin penetration enhancers

**DOI:** 10.1038/s41598-019-51226-5

**Published:** 2019-10-10

**Authors:** Monika Kopečná, Miloslav Macháček, Anna Nováčková, Georgios Paraskevopoulos, Jaroslav Roh, Kateřina Vávrová

**Affiliations:** 1 Skin Barrier Research Group, Charles University, Faculty of Pharmacy in Hradec Králové, Akademika Heyrovského 1203, 50005 Hradec Králové, Czech Republic; 2 Department of Biochemical Sciences, Charles University, Faculty of Pharmacy in Hradec Králové, Akademika Heyrovského 1203, 50005 Hradec Králové, Czech Republic; 3 Department of Organic and Bioorganic Chemistry, Charles University, Faculty of Pharmacy in Hradec Králové, Akademika Heyrovského 1203, 50005 Hradec Králové, Czech Republic

**Keywords:** Skin diseases, Drug development, Membranes

## Abstract

Skin penetration/permeation enhancers are compounds that improve (trans)dermal drug delivery. We designed hybrid terpene-amino acid enhancers by conjugating natural terpenes (citronellol, geraniol, nerol, farnesol, linalool, perillyl alcohol, menthol, borneol, carveol) or cinnamyl alcohol with 6-(dimethylamino)hexanoic acid through a biodegradable ester linker. The compounds were screened for their ability to increase the delivery of theophylline and hydrocortisone through and into human skin *ex vivo*. The citronellyl, bornyl and cinnamyl esters showed exceptional permeation-enhancing properties (enhancement ratios up to 82) while having low cellular toxicities. The barrier function of enhancer-treated skin (assessed by transepidermal water loss and electrical impedance) recovered within 24 h. Infrared spectroscopy suggested that these esters fluidized the *stratum corneum* lipids. Furthermore, the citronellyl ester increased the epidermal concentration of topically applied cidofovir, which is a potent antiviral and anticancer drug, by 15-fold. In conclusion, citronellyl 6-(dimethylamino)hexanoate is an outstanding enhancer with an advantageous combination of properties, which may improve the delivery of drugs that have a limited ability to cross biological barriers.

## Introduction

Drug application *via* the skin is an attractive non-invasive alternative to conventional routes of administration^1,2^. However, skin functions as an effective barrier protecting the organism from excessive water loss and absorption of potentially harmful compounds^3^. Hence, for the successful (trans)dermal administration of most drugs, the skin barrier properties must be temporarily suppressed^4^. To date, numerous approaches to overcoming the skin barrier, both physical and chemical, have been described^4–7^. Permeation enhancers enable drug administration by manipulating the skin barrier lipids, proteins or drug partitioning equilibria^4,6–8^. Unfortunately, the enhancer effects are often drug-specific and/or accompanied by skin irritation^9^. Thus, there is a continuous need for new/improved enhancement strategies.

**Figure 1:**
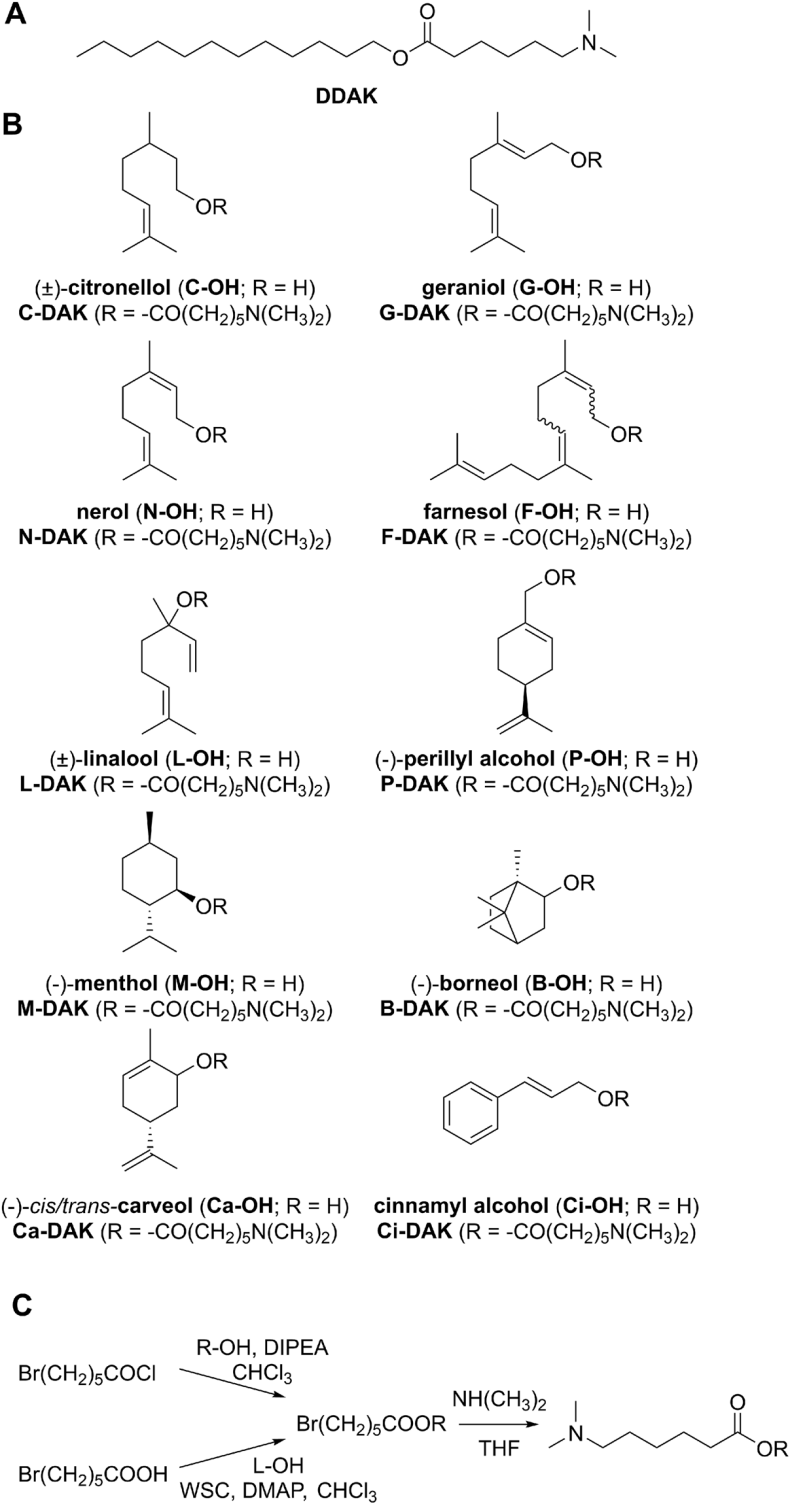
Structures of the studied enhancers – the parent amino acid ester DDAK (panel A), terpene alcohols and hybrid terpene-amino acid esters designed in this study (panel B). Two approaches were used for enhancer synthesis (panel C). Abbreviations: diisopropylethylamine (DIPEA), terpene alcohol (R-OH), linalool (L-OH), 4-(dimethylamino)pyridine (DMAP), 1-ethyl-3-(3-dimethylaminopropyl)carbodiimide (WSC), tetrahydrofuran (THF).

There are several groups of potent enhancers with low toxicity and limited irritation potential. Such compounds take advantage of natural compounds such as amino acids (*e.g*., proline derivative L-Pro2 and dodecyl 6-(dimethylamino)hexanoate – DDAK; Fig. 1A)^10,11^, and sugars (*e.g*., sorbitan monolaurate^12^, glucosyl and galactosyl derivatives^13,14^). The design of permeation enhancers as esters of nontoxic natural compounds was proven beneficial because such molecules are hydrolyzed by esterases into metabolites with low toxicity^11,15–18^.

Terpenes are a class of natural compounds with strong permeation-enhancing potential and have been generally recognized as safe (GRAS) adjuvants with relatively low and transient irritation^19,20^. For example, the acyclic monoterpene alcohols citronellol^21^, geraniol^22^, and linalool^23^, enhanced the permeation of ondansetron, caffeine and haloperidol, respectively. The cyclic monoterpenes borneol^24^, carveol^25^, menthol^26^, and limonene^27^ were reported as enhancers for ibuprofen, curcumin, indomethacin, and valsartan, respectively. In addition, sesquiterpene farnesol increased the permeation of haloperidol^28^.

In this study, new permeation enhancers were designed by combining the acyl part of the amino acid derivative DDAK (Fig. 1A) with a group of terpene alcohols. DDAK enhanced skin permeation of a broad spectrum of drugs, showed limited toxicity, reversible action and no dermal irritation^11,29,30^. In our hybrid terpene-amino acid enhancers, we exchanged the original dodecyl chain of DDAK for a terpene alcohol (Fig. 1B). To probe the structure-activity relationships in such enhancers, mono- and sesquiterpene alcohols with different levels of unsaturation, both acyclic and cyclic, with primary, secondary or tertiary hydroxyl groups were selected. Apart from the terpenes mentioned in the paragraph above, nerol (a *trans*-isomer of geraniol) and perillyl alcohol (a hydroxy derivative of limonene) were used. For comparison, another natural compound of nonterpene origin, cinnamyl alcohol, which enhanced skin permeation of ketoprofen^31^ and had negligible dermal toxicity and irritation, was included^32^.

The enhancing potencies of the prepared esters, along with all the parent terpene alcohols, DDAK, and dodecyl alcohol, were studied using human skin and two model drugs, theophylline (TH) and hydrocortisone (HC). Selected enhancers – citronellyl 6-(dimethylamino)hexanoate (C-DAK), bornyl 6-(dimethylamino)hexanoate (B-DAK) and cinnamyl 6-(dimethylamino)hexanoate (Ci-DAK) – were further probed for the reversibility of their action on human skin using transepidermal water loss (TEWL) and electrical impedance. The interactions of C-DAK, B-DAK and Ci-DAK with the barrier proteins and lipids in the uppermost epidermal layer, the stratum corneum (SC), were investigated using Fourier transform infrared spectroscopy (FTIR). The cellular toxicities of these enhancers were assessed in HaCaT keratinocytes and 3T3 fibroblasts, and the influence of these enhancers on the cell morphology was studied *via* confocal laser scanning microscopy. Finally, the effects of C-DAK, B-DAK and Ci-DAK on the delivery of cidofovir (CDV), a potent antiviral drug, through and into human skin were investigated.

## Results and Discussion

### Enhancer synthesis

Esters of terpene alcohols with 6-bromohexanoic acid were synthesized from 6-bromohexanoyl chloride and the respective terpene alcohol in 32–96% yields (Fig. 1). Primary alcohols reacted in 75–96% yields whereas terpenes with secondary hydroxyl groups afforded the esters in considerably lower yields (32–42%). The tertiary alcohol linalool did not react with 6-bromohexanoyl chloride; thus, the desired bromo ester was prepared from 6-bromohexanoic acid using carbodiimide/4-dimethylaminopyridine in a 21% yield. This manner of ester preparation can also be used to increase the yields of esters with secondary alcohols (for instance, borneol derivative was prepared in 50% yield compared to 32% using 6-bromohexanoyl chloride). Next, bromine was exchanged for the dimethylamino group in 58–83% yields. The final esters were oily compounds with MW 275–364 g/mol and logP values 3.3–5.5 (calculated using ChemDraw Professional 17.1, Supporting Table S1).

### Enhancing effects of the prepared compounds on delivery of theophylline (TH) and hydrocortisone (HC) through and into human skin

**Figure 2:**
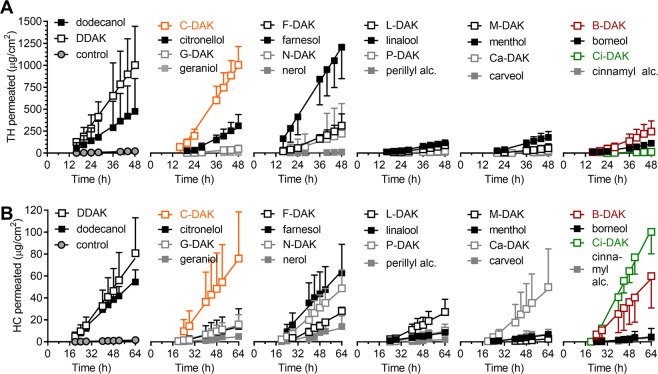
The effects of the studied enhancers on the permeation of the model drugs theophylline (TH, panel A) and hydrocortisone (HC, panel B) through human skin. Data are presented as the means ± SD; n ≥ 3. The flux values were calculated from the linear regions of the plots (mostly after 20 h). For flux values and statistical significance, see Tables 1 and 2.

The permeation-enhancing effects of the prepared compounds, along with the parent terpenes DDAK and dodecyl alcohol, were investigated in human skin using two model drugs: 5% TH or 2% HC in 60% aqueous propylene glycol (PG; Fig. 2, Tables 1 and 2). In previous studies, DDAK showed excellent enhancement efficacy when applied at 1%, which is ~30 mM^11,29^. Thus, all enhancers herein were tested at a 30 mM concentration. TH was selected as a small-molecule model permeant with balanced hydrophilic/lipophilic properties (M_w_ = 180 g/mol; logP = −0.02). The TH solubility in the donor solvent was ~27 mg/ml, and the studied compounds increased this value by 30% or less (Table 1). The second model permeant, HC, is a larger molecule with higher lipophilicity (M_w_ = 362 g/mol; logP = 1.61) than that of TH. The HC solubility in 60% PG was ~6 mg/ml, and the studied compounds did not significantly affect this value. Thus, all donor samples were applied at the maximum thermodynamic activities of the drugs.

**Table 1:** Effects of the studied enhancers (30 mM) on the skin permeability of a model drug theophylline (TH) applied at 5% w/v in 60% PG with or without the enhancer.

Compound	*C*_0_ (mg/ml)	*J*_*SS*_ (µg/cm^2^/h)	*ER*	*K*_*p*_ (×10^−5^ cm/h)	*C*_*Skin*_ (µg/mg)
control (no enhancer)	27.14 ± 1.67	0.68 ± 0.93	—	2.5	0.37 ± 0.34
dodecanol	27.11 ± 0.99	14.08 ± 9.58*	20.7	51.9	0.72 ± 0.35
DDAK	32.19 ± 2.20*	28.45 ± 8.62*	41.8	88.4	1.17 ± 0.42*
citronellol	29.77 ± 2.41	10.32 ± 3.25*	15.2	34.7	1.34 ± 0.30*
C-DAK	35.12 ± 3.16*	31.91 ± 4.60*^, **≠**^	46.9	90.9	1.18 ± 0.41*
geraniol	29.97 ± 0.06	0.25 ± 0.27	0.4	0.8	0.22 ± 0.09
G-DAK	34.97 ± 0.40*	1.97 ± 1.37	2.9	5.6	0.45 ± 0.09^**≠**^
nerol	30.33 ± 0.25	0.40 ± 0.33	0.6	1.3	0.28 ± 0.07
N-DAK	33.47 ± 0.32*	5.83 ± 4.19	8.6	17.4	0.66 ± 0.24^**≠**^
farnesol	27.97 ± 2.11	32.73 ± 7.51*	48.1	117.0	1.23 ± 0.18*
F-DAK	26.41 ± 0.99	8.84 ± 2.19^**≠**^	13.0	33.5	0.56 ± 0.14^**≠**^
linalool	28.59 ± 0.94	3.37 ± 1.11	5.0	11.8	0.50 ± 0.08
L-DAK	26.11 ± 0.56	2.42 ± 1.70	3.6	9.3	0.49 ± 0.14
perillyl alcohol	30.77 ± 0.15*	0.29 ± 0.01	0.4	0.9	0.29 ± 0.02
P-DAK	34.33 ± 0.45*	0.84 ± 0.95	1.2	2.4	0.40 ± 0.06^**≠**^
menthol	28.51 ± 1.75	5.60 ± 2.20	8.2	19.6	0.55 ± 0.10
M-DAK	26.52 ± 0.19	1.94 ± 1.53^**≠**^	2.9	7.3	0.45 ± 0.15
borneol	29.33 ± 1.26	3.28 ± 0.69	4.8	11.2	0.72 ± 0.19
B-DAK	32.46 ± 0.81*	7.64 ± 2.92^**≠**^	11.2	23.5	0.86 ± 0.08*
Carveol	30.23 ± 0.85	0.10 ± 0.06	0.1	0.3	0.29 ± 0.02
Ca-DAK	34.90 ± 0.53*	1.62 ± 2.18	2.4	4.6	0.54 ± 0.19
cinnamyl alcohol	31.27 ± 0.06*	0.07 ± 0.05	0.1	0.2	0.25 ± 0.08
Ci-DAK	35.27 ± 0.67*	0.57 ± 0.50	0.8	1.6	0.42 ± 0.04^**≠**^

Solubility of TH in the donor samples (*C*_0_), steady-state TH flux values (*J*_*SS*_), enhancement ratio (*ER*), permeability coefficient (*Kp*), concentration of TH in skin (*C*_*Skin*_). Data are presented as the means ± SD; n ≥ 3. *Statistically significant difference compared to negative control (without enhancer) at p < 0.05. ^**≠**^Statistically significant difference compared to the respective parent terpene at p < 0.05.

**Table 2:** Effects of the studied enhancers (30 mM) on the skin permeability of a model drug hydrocortisone (HC) applied at 2% w/v in 60% PG with or without the enhancer.

Compound	*C*_0_ (mg/ml)	*J*_*SS*_ (µg/cm^2^/h)	*ER*	*K*_*p*_ (×10^−5^ cm/h)	*C*_*Skin*_ (µg/mg)
control (no enhancer)	6.42 ± 0.89	0.03 ± 0.02	—	0.5	0.14 ± 0.05
dodecanol	6.97 ± 1.17	1.23 ± 0.33*	41.0	17.6	0.33 ± 0.07
DDAK	7.00 ± 1.05	1.70 ± 1.19*	56.7	24.3	0.23 ± 0.19
citronellol	7.20 ± 1.45	0.34 ± 0.31	11.3	4.7	0.45 ± 0.22
C-DAK	7.39 ± 1.35	1.67 ± 0.87*^, ≠^	55.7	22.6	0.24 ± 0.10
geraniol	7.79 ± 0.11	0.12 ± 0.02	4.0	1.5	0.58 ± 0.29*
G-DAK	7.69 ± 0.11	0.39 ± 0.18^≠^	13.0	5.1	0.29 ± 0.07
nerol	7.64 ± 0.07	0.31 ± 0.26	10.3	4.1	0.20 ± 0.06
N-DAK	7.54 ± 0.19	1.17 ± 0.36*^, ≠^	39.0	15.5	0.31 ± 0.11
farnesol	7.25 ± 1.00	1.31 ± 0.45*	43.7	18.1	0.21 ± 0.07
F-DAK	7.17 ± 0.64	0.72 ± 0.32^≠^	24.0	10.0	0.23 ± 0.04
linalool	6.41 ± 0.78	0.17 ± 0.05	5.7	2.7	0.25 ± 0.04
L-DAK	7.20 ± 1.14	0.66 ± 0.28^≠^	22.0	9.2	0.12 ± 0.02^≠^
perillyl alcohol	7.66 ± 0.02	0.07 ± 0.04	2.3	0.9	0.19 ± 0.05
P-DAK	7.87 ± 0.19	0.29 ± 0.16	9.7	3.7	0.30 ± 0.11
menthol	6.90 ± 0.90	0.16 ± 0.04	5.3	2.3	0.12 ± 0.03
M-DAK	7.03 ± 1.03	0.08 ± 0.06^≠^	2.7	1.1	0.13 ± 0.03
borneol	6.81 ± 1.18	0.16 ± 0.21	5.3	2.3	0.24 ± 0.02
B-DAK	6.96 ± 1.27	1.35 ± 0.60*^, ≠^	45.0	19.4	0.15 ± 0.06
carveol	7.51 ± 0.12	0.18 ± 0.09	6.0	2.4	0.19 ± 0.13
Ca-DAK	7.76 ± 0.46	1.14 ± 0.66*	38.0	14.7	0.31 ± 0.15
cinnamyl alcohol	7.39 ± 0.10	0.08 ± 0.06	2.7	1.1	0.31 ± 0.30
Ci-DAK	7.92 ± 0.16	2.47 ± 0.44*^, +, ≠^	82.3	31.2	0.20 ± 0.08

Solubility of HC in the donor samples (*C*_0_), steady-state HC flux values (*J*_*SS*_), enhancement ratio (*ER*), permeability coefficient (*Kp*), concentration of HC in skin (*C*_*Skin*_). Data are presented as the means ± SD; n ≥ 3. *Statistically significant difference compared to negative control (without enhancer) at p < 0.05. ^+^Statistically significant difference compared to DDAK (positive control) at p < 0.05. ^**≠**^Statistically significant difference compared to the respective parent terpene at p < 0.05.

The control TH sample without enhancer resulted in TH flux of 0.7 ± 0.9 µg/cm^2^/h (Fig. 2, Table 1). The permeability coefficient *K*_*p*_ was 2.5 × 10^−5^ cm/h, which is consistent with previous studies using human (2.1 × 10^−5^ cm/h)^13^ and porcine skin (1.1 × 10^–4^ cm/h)^10^. The HC flux through human skin was 0.03 ± 0.02 µg/cm^2^/h, giving a *K*_*p*_ value of 0.5 × 10^−5^ cm/h (Fig. 2, Table 2), which is comparable to reported values (2.99 × 10^−6^ and 3.0 × 10^−6^)^14,33^. The parent enhancer DDAK increased the TH and HC significantly over the control; the enhancement ratios (ER, calculated as the ratios of flux with and without an enhancer) were 42 and 57, respectively. These DDAK effects are consistent with previous data from porcine skin: the ER values for TH, HC, indomethacin (logP = 4.3, MW = 358 g/mol), and adefovir (logP = −2, MW = 273 g/mol) were 17, 43, 9, and 14, respectively^11^.

Citronellol was a mixture of naturally occurring (+) and (−) enantiomers, which are found in citronella and oil of rose, respectively. This acyclic monoterpene alcohol enhanced the flux of both TH (ER = 15) and HC (ER = 11). The citronellyl ester C-DAK was a significantly more potent enhancer than citronellol; ERs were equal to 47 and 56 for TH and HC, respectively. As citronellol was previously identified as a potent enhancer for ondansetron hydrochloride (ER = 31 in porcine skin)^21^, its structural modification to C-DAK holds potential for enhancing the skin permeation of a broader pool of drugs.

Introduction of a double bond in position 2 of citronellol leads to geraniol (*trans* isomer, the primary component of rose oil) and nerol (*cis* isomer, found in lemongrass). This desaturation was apparently detrimental for their enhancing efficacy towards TH, as geraniol, nerol, as well as their esters G-DAK and N-DAK did not have significant effects on the TH permeation. However, nerol ester N-DAK showed ER = 39 for HC. Notably, the enhancing efficacies of *cis* isomer N-DAK were 3-fold higher (not significant for TH, significant for HC) than those of *trans* isomer G-DAK. The higher efficacy of the *cis* isomer in contrast to the *trans* was previously observed for oleic (*cis)* and elaidic (*trans*) acids^34^.

A sesquiterpene farnesol (a component of many essential oils, *e.g*., citronella) enhanced the skin absorption of both TH and HC (ERs = 48 and 44, respectively). The stronger farnesol effect compared to that of the monoterpenes is consistent with a previous study using diclofenac^35^. Unfortunately, farnesol esterification to F-DAK reduced its efficacy for both model drugs. A similar reduction of efficacy upon esterification was observed for cyclic monoterpene menthol (the TH and HC flux values were 2–3-fold lower with M-DAK than with menthol). The tertiary alcohol linalool, which is found in, *e.g*., lavender, and its ester L-DAK enhanced neither TH nor HC permeation.

Esterification of the bicyclic monoterpene borneol (which is found in numerous plants as well as in castoreum) to B-DAK resulted in a significant increase in TH and HC flux (ER = 11 and 45, respectively) compared to that of borneol. The cyclic monoterpenes carveol (secondary alcohol, a constituent of spearmint) and perillyl alcohol (primary alcohol, a constituent of, *e.g*., lavender) are hydroxylated derivatives of limonene. However, perillyl alcohol, carveol, and P-DAK did not significantly enhance TH and HC permeation. Carveol ester Ca-DAK was equally inactive for TH but enhanced skin permeability of HC (ER = 38).

The strongest permeation-enhancing effect for HC was found for cinnamyl alcohol ester Ci-DAK (ER = 82). This Ci-DAK effect was also higher than the effect of DDAK by a factor of 1.5 (or 1.3 when considering their *K*_*p*_ values). In contrast, the skin permeability of TH was not affected by Ci-DAK.

Next, the amount of the model drugs TH and HC remaining in the skin were determined (Tables 1 and 2, respectively). Without enhancers, the TH amount found in the skin was 0.37 ± 0.34 µg/mg. DDAK, citronellol, C-DAK, and farnesol increased the TH concentration in the skin 3–4-fold. The effects of other compounds were weaker than those of DDAK. The studied compounds did not significantly affect the amount of HC retained in the skin (0.14 ± 0.05 µg/mg), with the exception of geraniol, which increased the HC skin concentration 4-fold.

Thus, among the primary terpene alcohols studied here, only citronellol, farnesol, and dodecanol showed significant TH and HC flux-enhancing efficacies at 30 mM. This molar concentration corresponds to 0.4% (cinnamyl alcohol) − 0.7% w/v (farnesol), which is approximately an order of magnitude lower than the concentrations used in previous studies with terpene enhancers^22,23,36^. The secondary alcohols menthol and borneol and the tertiary alcohol linalool were inactive. Cyclic terpenes were also inactive, which is consistent with the previously reported lower efficacy of cyclic *vs*. acyclic terpenes for diclofenac^37^. In general, the lack of enhancing effect of 30 mM monoterpene alcohols for TH and HC indicates drug-, dose- and/or skin-specific effects of these terpene enhancers.

Esterification of the natural alcohols with 6-(dimethylamino)hexanoic acid had generally positive effects on their efficacies, except for menthol, linalool (with TH), and farnesol. These increased efficacies of the esters may be partly related to their higher lipophilicities compared to their parent alcohols (Supporting Table S1). More lipophilic enhancers would have higher affinities to the highly hydrophobic SC lipid matrix. Such enhancers could incorporate into the hydrophobic tail regions of the SC lipids, disrupt their tight organization, and facilitate diffusion of lipid-like penetrants^7^. Williams and Barry also suggested that more lipophilic terpenes are better enhancers for lipophilic drugs than more hydrophilic terpenes^8^. However, our previous studies suggested that the structure-activity relationships in enhancers are more complex, and enhancer lipophilicity is only one contributing factor to their ability to promote drug absorption through the skin^18,38^. Actually, we have recently described complex interactions among the enhancer, drug, solvent and skin during the permeation process^39^.

Thus, some of the prepared esters strongly enhanced the permeation of the model drugs TH and HC through human skin with no or weak effects on their retention in the skin. Notably, the terpene derivatives described here are much stronger enhancers for TH than the previously described sugar-based compounds^13,14^. For further experiments, we selected the acyclic citronellol derivative C-DAK, which was a potent enhancer for both TH and HC, and bicyclic borneol ester B-DAK, which had high enhancing efficacy for HC and a moderate effect on TH. For comparison, a derivative of cinnamyl alcohol Ci-DAK, which showed excellent efficacy for HC, was also included.

### Cellular toxicities of B-DAK, C-DAK and Ci-DAK enhancers

**Figure 3:**
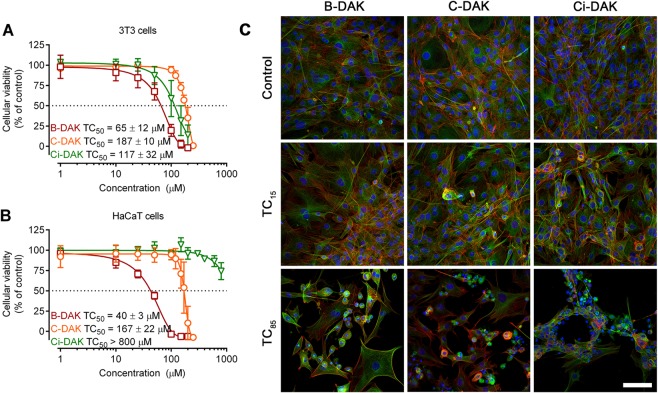
The viability of mouse fibroblast and human keratinocyte cell lines (panels A and B, respectively) after 48 h of incubation with the selected enhancers B-DAK, C-DAK and Ci-DAK. Data are presented as the means ± SD; n ≥ 3. Panel C: Morphological changes of 3T3 cells treated with low (TC_15_) and high (TC_85_) doses of enhancers. Cells were stained for nuclei (blue), microfilaments (green) and microtubules (red). Bar represents 100 µm.

To estimate the safety of the selected enhancers B-DAK, C-DAK and Ci-DAK, their cellular toxicities were assessed using two cell lines: HaCaT keratinocytes and 3T3 fibroblasts. The toxicity is reported as the TC_50_ value, *i.e*., the enhancer concentration that caused a 50% decrease in cell viability after 48 h incubation (as the 24-h incubation did not lead to significant toxicities). B-DAK had TC_50_ values in tens of µM (Fig. 3) whereas C-DAK and Ci-DAK had negligible toxicities (TC_50_ over 160 µM and over 110 µM, respectively). Notably, the Ci-DAK toxicities strongly depended on the cell type: the TC_50_ was 117 ± 32 µM in 3T3 cells, whereas only a 30% decrease in HaCaT viability was detected at 800 µM.

These toxicities of C-DAK and Ci-DAK are comparable or lower than those of the parent DDAK (TC_50_ values in 3T3 and HaCaT cells were 175 ± 28 and 76 ± 13 µM, respectively) or the L-proline-based enhancer L-Pro2 (TC_50_ values in 3T3 and HaCaT cells were 183 ± 7 and 68 ± 12 µM, respectively)^10^. Notably, L-Pro2 showed no dermal toxicity in rats *in vivo*^10^. Thus, C-DAK seems to have a beneficial combination of high permeation enhancing potency for both model drugs and low toxicity.

Confocal microscopy demonstrated that treatment with enhancers at their TC_15_ (which is over 100 µM for C-DAK) only slightly increased the number of cells undergoing apoptosis (Figs 3, S1). Treatment with enhancers at their TC_85_ (which are extremely high concentrations, for example, ~200 µM for C-DAK) led to morphological features typical of apoptotic cell death, such as retraction or rounding of the cells, membrane blebs, redistribution of both microfilaments and microtubules or loss of the signal of both cytoskeleton molecules, pyknosis, and karyorrhexis. The unaffected cells retained their typical morphological features. Importantly, no signs of necrosis that would initiate undesirable inflammatory reactions *in vivo* were detected^40^.

These relatively low cellular toxicities of C-DAK and B-DAK are consistent with the low toxicities of their precursor alcohols, citronellol and borneol^19,20^. Cinnamyl alcohol and its esters have also been described as compounds without safety concerns^32^. Considering the biodegradability of the enhancers, DDAK was hydrolyzed *in vitro* by a porcine esterase with a half-life of 17 min^11^. Recently, we described the hydrolysis of structurally similar ester enhancers in freshly excised *ex vivo* human skin (mostly in living epidermis, but some hydrolytic products were detected in lower SC layers)^39^. Hence, C-DAK and Ci-DAK, which are esters of primary alcohols, are expected to be hydrolyzed into nontoxic metabolites after they exert their permeation-enhancing effects and reach deeper skin layers.

### Reversibilities of the effects of B-DAK, C-DAK and Ci-DAK enhancers on transepidermal water loss (TEWL) and electrical impedance

**Figure 4:**
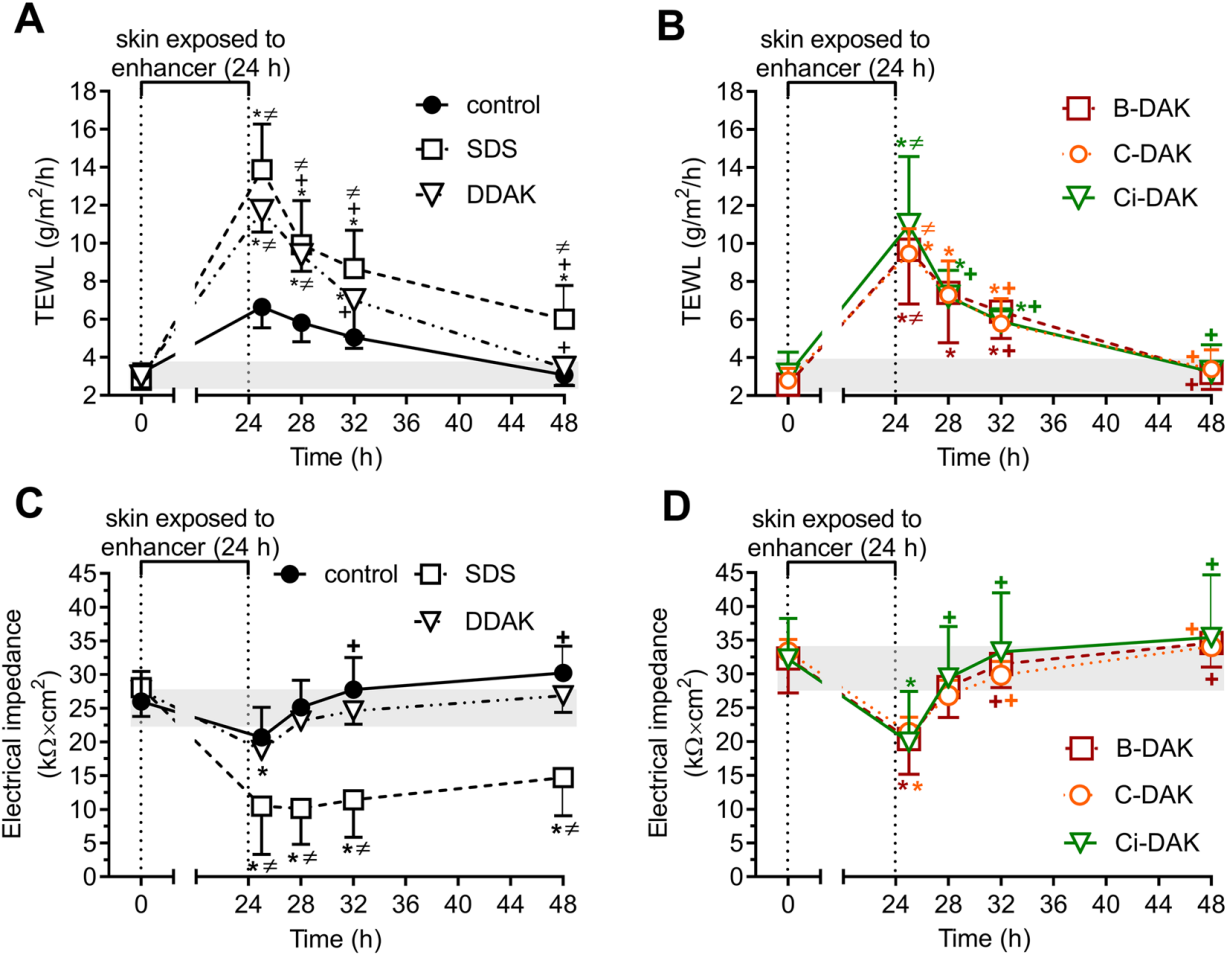
Reversibility of the effects on TEWL (panels A and B) and electrical impedance of human skin (**C,D**) of selected enhancers (DDAK, B-DAK, C-DAK and Ci-DAK) and SDS compared to respective control solvents without enhancers (60% PG). All samples were applied for 24 h and then removed. Gray areas indicate the baseline values before treatment. Data are presented as the means ± SD; n ≥ 3. *Significant compared to the value before enhancer application at p < 0.05. ^+^Significant compared to the value after the enhancer removal (time 25 h) at p < 0.05. ^**≠**^Significant compared to the control (60% PG without enhancer) at the same time point, at p < 0.05.

Because the selected enhancers had low cellular toxicities, their effects on skin were further studied. To probe the direct effects of B-DAK, C-DAK and Ci-DAK on the skin barrier without any possible interactions with the drugs, we examined the TEWL and electrical impedance of the skin before and after the 24 h enhancer treatment (Supporting Table S2 and Fig. 4). TEWL is a method commonly used in dermatology to estimate the water permeability barrier^41^. To eliminate possible adverse effects of the donor samples on the TEWL probe, TEWL was measured 1 h after the donor samples had been removed from the skin. The basal TEWL values were 2.6–3.4 g/m^2^/h. The control sample (60% PG) applied for 24 h did not significantly change TEWL, which is consistent with our previous studies^13,14^. DDAK, B-DAK, C-DAK and Ci-DAK increased TEWL 3–4-fold (significant over the baseline TEWL as well as the TEWL found in the solvent-treated control samples).

The direct enhancer effects on the skin barrier were further assessed using electrical impedance, which is reciprocally related to the permeability of the skin to ions. This parameter (or its reciprocal, conductivity) was previously used to characterize the effects of enhancers^10,13,14^ or in their high-throughput screening^42^. Skin impedance values before the sample application were 26–33 kΩ × cm^2^. The 60% PG (control) did not significantly change the impedance value (consistent with previous data^10,13,14^). The enhancers B-DAK, C-DAK and Ci-DAK significantly decreased the skin impedance to 62–64% of the baseline values, which is comparable to the effect of DDAK. Thus, both TEWL and impedance showed that the studied enhancers directly influenced skin barrier function.

The action of chemical enhancers on the skin should be reversible to prevent the access of undesirable compounds from the environment into the body or excessive water loss^8^. As TEWL and impedance were significantly altered by enhancer treatment, we used these two markers to determine whether the enhancer-treated skin barrier returns fully or partially to the values observed in untreated skin after the enhancer was removed from the skin.

We used the donor solvent (60% PG) without enhancers as the negative control. In addition, we included another control, sodium dodecyl sulfate (SDS), which should elicit an irreversible effect. The SDS effects on skin barrier properties are not fully reversible, as demonstrated by Löffler and Happle, who found increased TEWL values in human volunteers even 10 d after the SDS had been removed from the skin^43^. In our setup, SDS application increased TEWL values almost 5-fold (Fig. 4). Within the next 24 h, TEWL decreased by approximately half but remained significantly higher than the basal TEWL and TEWL of the control (PG-treated) skin at the same time point. This result was confirmed by electrical impedance. SDS application significantly decreased the impedance to 37% of the value measured for untreated skin. After SDS removal from the skin, the impedance values remained approximately half of the basal value of untreated skin for at least 24 h.

In contrast, TEWL of the skin treated with DDAK, B-DAK, C-DAK and Ci-DAK returned to the levels comparable to the basal (untreated) TEWL within 24 h after the enhancer removal (~3 g/m^2^/h, Fig. 4). Electrical impedance confirmed these reversible effects as the impedance returned within 4 h to the values observed before treatment. Hence, both methods are suitable to distinguish between the reversible and irreversible effects of compounds on skin barrier properties, as suggested in our previous study of sugar-based enhancers^13,14^.

### Interaction of B-DAK, C-DAK and Ci-DAK enhancers with the skin barrier probed by FTIR spectroscopy

To elucidate the manner in which the studied enhancers decrease the skin barrier properties, we used FTIR spectroscopy of isolated human SC. FTIR is a powerful method to investigate the interactions of enhancers with SC lipids or proteins^44^. No protein changes, as deduced from the amide I vibrations^45,46^, were observed in the SC treated with 30 mM B-DAK, C-DAK or Ci-DAK for 24 h compared to the control (60% PG-treated SC), which is consistent with the reversibility of their action.

**Figure 5:**
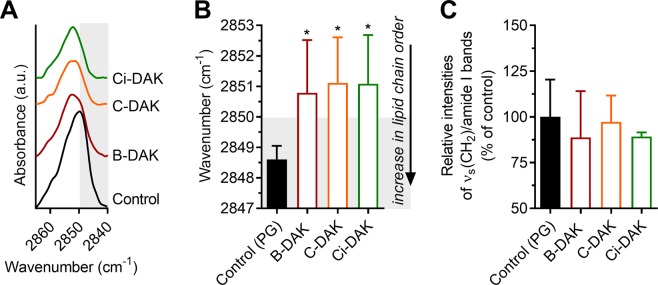
Methylene symmetric stretching vibration after the treatment of human SC by selected enhancers (panel A – example spectra, B – wavenumbers). Panel C – Relative intensities of the methylene symmetric stretching band to amide I band. Data are presented as the means ± SD; n ≥ 3. *Statistically significant difference compared to control (60% PG without enhancer) at p < 0.05.

In contrast, the enhancers significantly decreased the overall SC lipid chain order, as suggested by the shifts of the methylene symmetric stretching vibration from 2848.6 cm^−1^ in control PG-treated SC (which is indicative of well-ordered lipid chains with prevailing *all-trans* conformers)^46^ to 2850.8 cm^−1^ (B-DAK) and 2851.1 cm^−1^ (C-DAK and Ci-DAK; Fig. 5). The wavenumbers over 2850 cm^−1^ suggest increased proportion of less ordered *gauche* conformers in the SC lipid matrix^46^. These wavenumber shifts caused by B-DAK, C-DAK and Ci-DAK were accompanied by peak broadening by 1.4, 0.8 and 2.0 cm^−1^, respectively (the full width at half maximum values were 5.7 ± 0.8, 5.1 ± 0.6, and 6.3 ± 0.7 cm^−1^), compared to the control (4.3 ± 0.9 cm^−1^), which further supports the presence of less ordered chains.

In addition, no significant decreases in the intensity ratios of methylene to amide I bands were observed compared to the control (these simple lipid/protein ratios in the SC treated with B-DAK, C-DAK and Ci-DAK were 89%, 97%, and 89% of that of the control, respectively, Fig. 5C). Thus, these enhancers did not cause any significant lipid extraction at 30 mM concentrations, which is in contrast with previous studies using terpene alcohols (*e.g*., 5% linalool in PG^23^ or 10% nerol in PG^47^). The lack of lipid extraction by our enhancers may be explained by the acylation of the parent terpenes with 6-(dimethylamino)hexanoic acid and/or their 5–10-fold lower concentrations compared to those of the abovementioned studies.

Similar enhancer interactions with the skin barrier, *i.e*., involving lipids but not proteins, were found with other terpenes^20^, Azone^48^ and other amphiphilic enhancers (based on amino acids^10,39^ or sugars^13,14^). Theoretically, the observed enhancer effects on the SC lipids indicate either lipid fluidization or the presence of separated pools of disordered enhancer chains. As these enhancer tails are largely branched/cyclic and unsaturated, they actually have only a few methylene groups that would interfere with the methylene symmetric stretching band of SC lipids. Thus, we assume that the wavenumber shifts are mostly caused by the disordering/fluidization of the SC lipid chains. These results are in good agreement with Pham *et al*., who studied enhancer interactions with SC using solid-state NMR spectroscopy and found that monoterpenes increase lipid acyl chain mobility but generate no or only minor changes in the molecular mobility of SC proteins^48^.

### Effect of selected enhancers on the delivery of antiviral cidofovir (CDV) into and through human skin

Finally, we explored the enhancing potencies of the selected enhancers B-DAK, C-DAK and Ci-DAK using a clinically relevant drug that would benefit from improving its delivery through/into skin, a potent antiviral CDV^49^. CDV is a hydrophilic derivative of deoxycytidine monophosphate, which is active against skin tumors and infections caused by a wide spectrum of viruses (*e.g*., polyoma-, papilloma-, adeno- and pox-virus, human herpes viruses, varicella-zoster- or Epstein-Barr virus)^50^. CDV was applied at 1% solution in citrate buffer at pH 6. The amounts of this acyclic nucleoside phosphonate in the acceptor compartment, with or without enhancers, were close to or below the detection limit. This was expected because these highly hydrophilic (logP = −3.9) drugs do not readily cross biological barriers as found for CDV^13,14^, adefovir^29^, and 2,6-diaminopurine antivirals^30^. Thus, we only report here the CDV concentrations retained in the epidermis and dermis.

**Figure 6:**
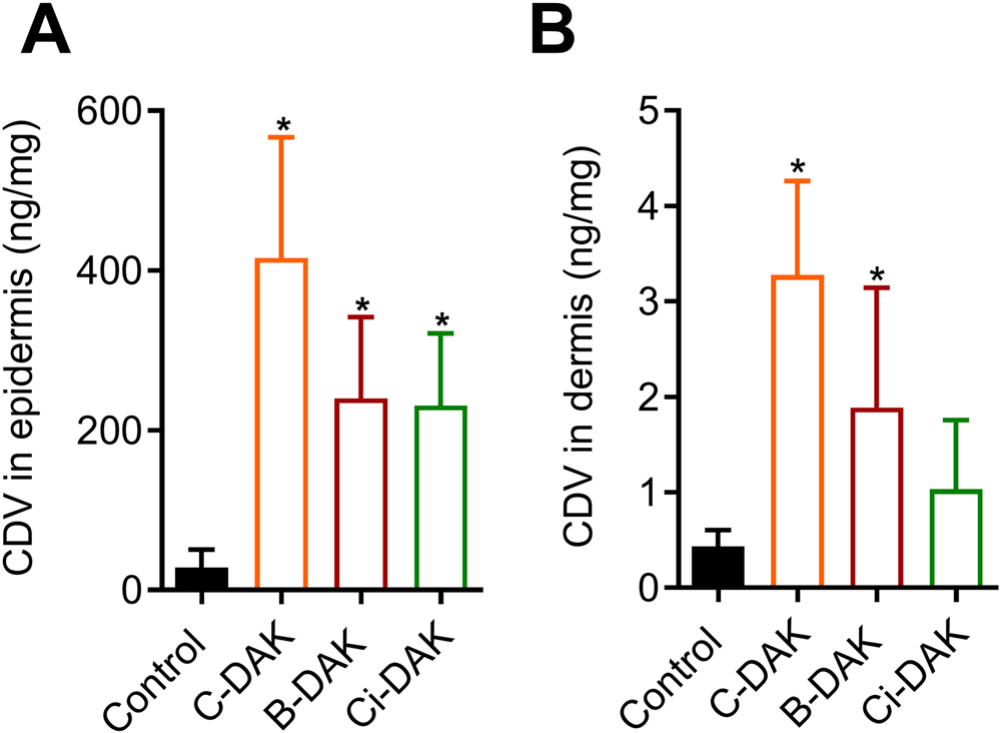
Effects of selected enhancers B-DAK, C-DAK and Ci-DAK (30 mM in citrate buffer at pH 6) on CDV concentration in human epidermis (**A**) and dermis (**B**). No CDV was found in the acceptor compartment. Data are presented as the means ± SD; n = 5. *Significant compared to control (without enhancer) at p < 0.05.

Without enhancers, only 28.4 ± 22.5 ng CDV per mg of epidermis was found (Fig. 6A). C-DAK, B-DAK and Ci-DAK caused a 15-fold, 9-fold and 8-fold increase in the epidermal CDV concentrations, respectively. The dermal CDV concentration was 0.44 ± 0.17 ng/mg in the control without enhancers (Fig. 6B). C-DAK and B-DAK significantly increased this value (8- and 4-fold, respectively). Thus, B-DAK, C-DAK and Ci-DAK selectively augmented CDV accumulation in the human epidermis without increasing its permeation into the receptor compartment. These effects appear promising for the treatment of skin viral infections or tumors sensitive to CDV.

## Conclusion

Skin penetration/permeation enhancers have a strong potential for improving drug delivery through and into the skin. The wider use of enhancers in transdermal/topical formulations has been hampered by the inherent toxicities or irritation potential of these substances. We show here that enhancers designed as esters of natural alcohols (citronellol, borneol and cinnamyl alcohol) and 6-(dimethylamino)hexanoic acid combine high potencies with safe toxicity profiles. In addition, the effects of these compounds on the skin barrier function are temporary and involve mostly fluidization of the skin barrier lipids. In particular, citronellyl 6-(dimethylamino)hexanoate increased the TH flux 47-fold, the HC flux 50-fold, and the epidermal concentration of CDV 15-fold, with TC_50_ values in HaCaT and 3T3 cell lines exceeding 160 µM.

## Materials and Methods

### Synthesis of enhancers

See Supporting Information.

### Donor samples

Donor samples for the skin permeation experiments were prepared as follows: a 5% (w/v) TH suspension in 60% propylene glycol (PG), 2% (w/v) HC suspension in 60% PG, and 1% (w/v) CDV solution in citrate buffer (0.1 M, pH = 6). All samples were prepared with or without 30 mM enhancers. The enhancing potentials of the newly prepared esters for TH and HC were compared to the respective terpene alcohols, dodecanol, and DDAK (all at 30 mM). The samples were thoroughly mixed, equilibrated at 32 °C for 24 h and carefully re-suspended prior to their application on the skin. Samples for FTIR and reversibility studies were prepared in 60% PG without the model drug in the same manner as those for permeation studies. For the effects of the studied compounds on the drug solubility in the donor solvent, *C*_0_^13,14^, see Supporting Information.

### Human skin

Human skin from Caucasian female patients who underwent abdominal plastic surgery was used with the approval of the Ethics Committee of the Sanus Surgical Centre (4/5/2018), according to the principles of the Declaration of Helsinki. Informed consent has been obtained. The subcutaneous fat was carefully removed, and the remaining full-thickness skin fragments were washed with saline, blotted dry and stored at −20 °C.

### Permeation experiments

The effects of the prepared compounds on human skin permeability were studied *in vitro* using Franz diffusion cells with a permeation area of 1 cm^2^ and phosphate-buffered saline at pH 7.4 (PBS) as the acceptor phase^13,14,39^; see Supporting Information for details. An infinite dose (150 µl) of the respective donor sample was applied on the skin surface. The experiments with TH, HC and CDV were conducted for 48, 64, and 72 h, respectively, to reach steady-state conditions.

The drug concentrations in the acceptor samples were determined by HPLC (Supporting Information). The cumulative amount of TH or HC (CDV was not detected in the acceptor), which permeated through the skin, corrected for the acceptor phase replacement, was plotted against time. The flux of the model drug through the skin *J*_*ss*_ (µg/cm^2^/h) was calculated from the linear region of the permeation curve. For comparison, free SAMPA software was also used for the calculation of flux^51^. The flux values calculated by these two methods did not significantly differ. The permeability coefficient *K*_*p*_ (cm/h) is the value of *J*_*ss*_ divided by the concentration of model drug in the donor sample *C*_0_ (mg/ml). The enhancement ratio (ER) was calculated as the ratio of flux with and without an enhancer.

At the end of the permeation experiment, the Franz cells were dismounted, and the skin was washed with PBS. The area of skin exposed to the donor sample was punched out, weighed and extracted by 5 ml of an extraction solvent for 24 h (for TH and HC). For CDV extraction, the skin samples were heated to 80 °C for 2 min, and the epidermis was peeled off from the dermis with tweezers. Epidermis and dermis were weighed and extracted with 1 and 2 ml of an extraction solvent, respectively, for 24 h. The skin extract was filtered and analyzed by HPLC (Supporting Information). The extraction solvent composition was the same as that of the HPLC mobile phase for the appropriate model drug^13,14^.

### Cellular toxicities of enhancers and confocal microscopy

The toxicities of selected enhancers were evaluated on two widely used non-malignant cell lines: HaCaT spontaneously immortalized human keratinocytes and 3T3 Swiss albino mouse embryonic fibroblasts. The cell lines were cultivated as described previously^13,14^. For toxicity studies and confocal microscopy, cells were seeded at a density of 10,000 cells per well into 96-well plates and 100,000 cells per well into 4-well cell-imaging slides. Cell viability was determined using a 3-(4,5-dimethylthiazol-2-yl)-2,5-diphenyltetrazolium bromide (MTT) uptake assay after 48 h incubation with the enhancer^13,14^, see Supporting Information for details.

Morphological changes were evaluated using laser scanning confocal microscopy using a Nikon A1 + confocal system after 48 h incubation of the cells with enhancers using concentrations of compounds corresponding to their TC_15_ and TC_85_ as described before^13,14^. Staining for actin and tubulin cytoskeleton was performed for 90 min using 5 U/mL Alexa Fluor 555 phalloidin and 2 µg/mL α-tubulin antibody and Alexa Fluor 488 conjugate, respectively (Supporting Information).

### Reversibility of enhancer effects

Human skin was mounted into Franz cells in the same manner as for the permeation experiments. After 1 h of equilibration at 32 °C, baseline electrical impedance and transepidermal water loss (TEWL) values of the skin samples were measured by LCR meter 4080 and Tewameter® TM 300, respectively (Supporting Information)^13,14^. Then, the skin samples received 150 µl of donor sample containing either 60% PG (negative control), 30 mM enhancer in 60% PG, or 5% (*w/v)* sodium dodecyl sulfate in 60% PG (SDS, positive control). After 24 h, the donor samples were cautiously removed, and the exposed skin surface was washed with PBS and blotted dry. Next, the electrical impedance and TEWL were measured at predetermined time points over 24 h, as described previously^13,14^.

### Fourier transform infrared (FTIR) spectroscopy

The effects of the selected enhancers on the skin barrier lipids and proteins were studied on isolated human SC using FTIR spectroscopy. Heat-separated human epidermis (60 °C for 2 min) was incubated overnight with trypsin to obtain SC sheets^52^. Hydrated SC sheets (approximately 1 mg) were treated with 60% PG (control) or 30 mM enhancers in 60% PG at 32 °C for 24 h. Then, the excess donor sample was carefully removed from the SC surface, and FTIR spectra were recorded on a Nicolet 6700 FT-FTIR spectrometer equipped with a single-reflection MIRacle attenuated total reflectance ZnSe crystal. The spectra were generated by co-addition of 128 scans recorded at a 2 cm^−1^ resolution and analyzed using Bruker OPUS software.

## Supplementary information

Supplementary info
