# Supplementary material for: Esters of terpene alcohols as highly potent, reversible, and low toxic skin penetration enhancers
Source: Sci Rep. 2019 Oct 10;9:14617. doi: 10.1038/s41598-019-51226-5 (PMC6787078; doi:10.1038/s41598-019-51226-5)

SUPPORTING INFORMATION

Esters of terpene alcohols as highly potent, reversible, and low toxic skin penetration enhancers

Monika Kopečná[†], Miloslav Macháček[‡], Anna Nováčková[†], Georgios Paraskevopoulos[†], Jaroslav Roh[§], Kateřina Vávrová[†,*]

[†]Skin Barrier Research Group, [‡]Department of Biochemical Sciences, and [§]Department of Organic and Bioorganic Chemistry, Charles University, Faculty of Pharmacy in Hradec Králové, Akademika Heyrovského 1203, 50005 Hradec Králové, Czech Republic

* katerina.vavrova@faf.cuni.cz

CONTENTS:

Supporting Tables and Figures

- Table S1 – Properties of the studied compounds (p. 2)
- Table S2 – TEWL and electrical impedance of enhancer-treated human skin (p. 2)
- Figure S1 - Morphological changes of enhancer-treated HaCaT cells (p. 3)

Supporting experimental procedures

- Synthesis of enhancers (p. 4)
- Characterization of the prepared compounds (p. 4-9)
- Drug solubility in donor samples (p. 9)
- Permeation experiments (p. 9-10)
- High-Performance Liquid Chromatography (HPLC) (p. 10)
- Cellular toxicities of enhancers (p. 10-11)
- Laser scanning confocal microscopy (p. 11)
- Skin electrical impedance (p. 11)
- Transepidermal water loss (TEWL) (p. 11-12)

References (p. 12)

Copies of NMR spectra (p. 13-32)

Table S1. Properties of the studied compounds (MW and logP - calculated using ChemDraw Professional 17.1).

alcohol	M_w [g/mol]	logP	ester	M_w [g/mol]	logP
**dodecanol**	186.34	4.31	**DDAK**	327.55	5.76
**citronellol**	156.27	2.82	**C-DAK**	297.48	4.28
**geraniol**	154.25	2.49	**G-DAK**	295.47	3.94
**nerol**	154.25	2.49	**N-DAK**	295.47	3.94
**farnesol**	222.37	4.01	**F-DAK**	363.59	5.47
**linalool**	154.25	2.55	**L-DAK**	295.47	4.00
**perillyl alc.**	152.24	1.95	**P-DAK**	293.45	3.41
**menthol**	156.27	2.75	**M-DAK**	297.48	4.20
**borneol**	154.25	2.43	**B-DAK**	295.47	3.88
**carveol**	152.24	1.92	**Ca-DAK**	293.45	3.38
**cinnamyl alc.**	134.18	1.98	**Ci-DAK**	275.39	3.43

Table S2. Transepidermal water loss (TEWL) and electrical impedance of enhancer-treated human skin.

Compound	Before enhancer treatment	1 h after enhancer treatment	24 h after enhancer treatment
		**TEWL ($g/m^2/h$)**	
**Control**	3.43 ± 0.46	6.13 ± 0.95	3.28 ± 0.17
**DDAK**	3.00 ± 0.60	11.70 ± 1.11 *	3.45 ± 0.94
**B-DAK**	2.58 ± 0.59	9.68 ± 2.86 *	3.20 ± 0.88
**C-DAK**	2.80 ± 0.64	9.48 ± 1.31 *	3.40 ± 1.01
**Ci-DAK**	3.10 ± 1.18	10.98 ± 3.60 *	3.25 ± 1.43
		**Electrical impedance ($k\Omega \times cm^2$)**	
**Control**	25.99 ± 4.50	20.69 ± 4.44	30.25 ± 3.98
**DDAK**	27.66 ± 0.94	18.75 ± 0.72 *	26.85 ± 2.47
**B-DAK**	32.29 ± 5.10	20.36 ± 5.20 *	34.60 ± 3.57
**C-DAK**	33.28 ± 1.85	21.35 ± 2.26 *	34.05 ± 1.95
**Ci-DAK**	32.24 ± 5.98	19.91 ± 7.51 *	35.42 ± 9.27

Data are presented as means ± SD; n ≥ 3. Control = 60% PG without enhancer. * statistically significant difference compared with the value before application of an enhancer at $p < 0.05$.

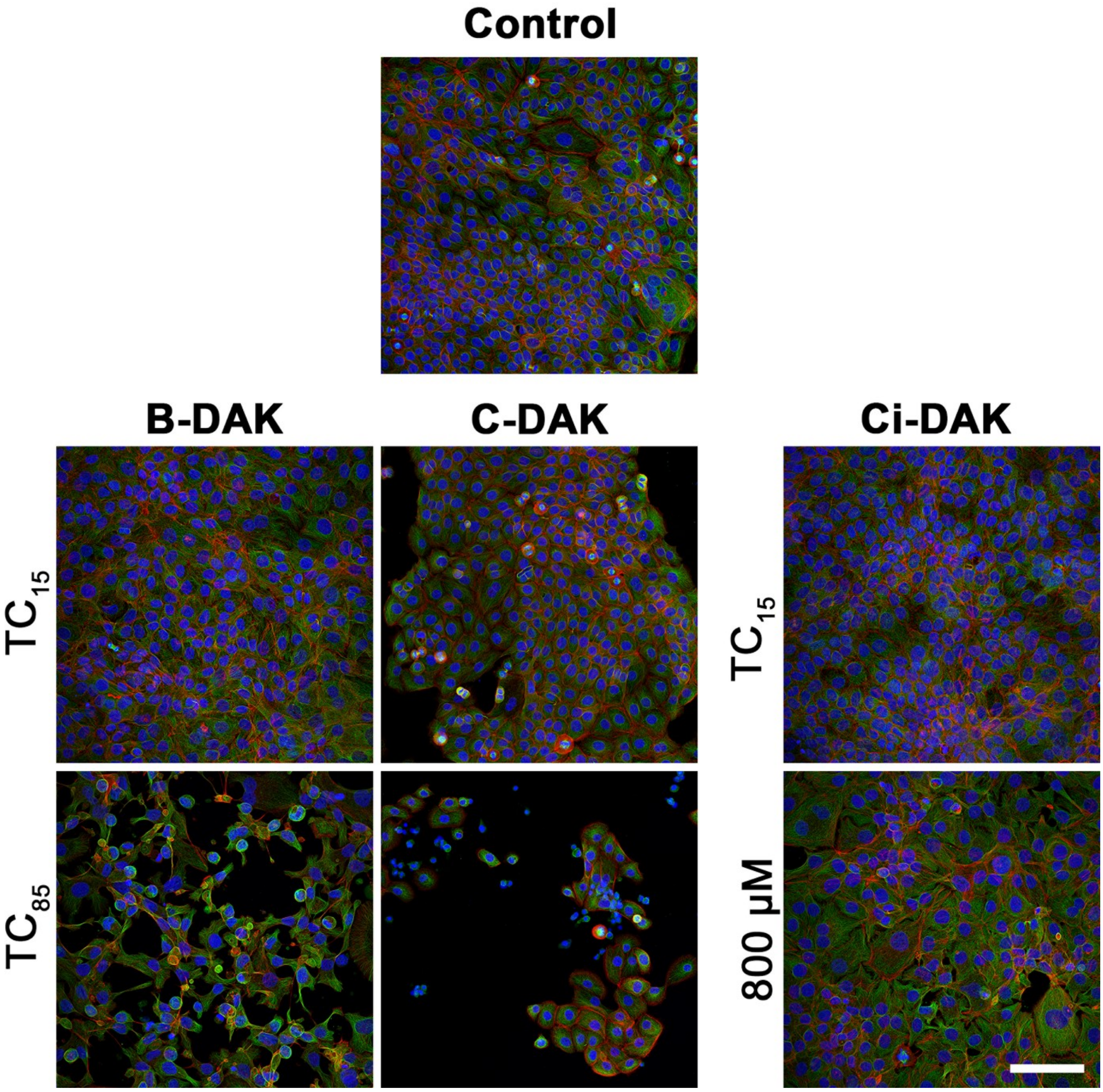

Figure S1 - Morphological changes of HaCaT cells treated with low (TC_{15}) and high (TC_{85}) dose of enhancers. Cells were stained for nuclei (blue), microfilaments (green) and microtubules (red). Bar represents 100 µm.

Synthesis of enhancers

All chemicals used for synthesis were obtained from Sigma-Aldrich (Germany). Reactions and column chromatography were monitored by TLC on aluminum plates with silica gel 60 F254. Column chromatography was performed using Merck Kieselgel 60 (0.040-0.063 mm). The structure and identity of synthesized compounds were confirmed by ^{1}H-NMR, ^{13}C-NMR and FTIR spectroscopy and by mass spectrometry. ^{1}H and ^{13}C NMR spectra were recorded using a Varian Mercury Vx BB 300 or VNMR S500 NMR spectrometer (Varian, CA, USA). Chemical shifts were indirectly referred to tetramethylsilane (TMS) via the solvent signal and reported as δ values in parts per million (ppm). FTIR spectra of studied compounds were recorded on a Nicolet 6700 FTIR spectrometer (Thermo Scientific, USA) in an ATR mode. Mass spectrometry was recorded with an Agilent 500 Ion Trap LC/MS (Agilent Technologies, CA, USA). Optical activity of prepared compounds was measured using polarimeter P 3000 (Krüss, Germany) after dissolving of the samples in $CHCl_3$ in concentration of 0.01 g/ml.

General procedure for the preparation of 6-bromohexanoic acid esters: 2 mmol of the appropriate alcohol and 2 equivalents (4 mmol) of diisopropylethylamine were dissolved in 5 ml of dry chloroform. Subsequently, 1 equivalent of 6-bromohexanoyl chloride was added dropwise, and the reaction mixture was stirred at room temperature for 20 h. The reaction was monitored by TLC on silica gel using a hexane:ethyl acetate 9:1 (v/v) mobile phase. The reaction mixture was washed with 1 M HCl, saturated $NaHCO_3$, and water and dried under reduced pressure. Linalool did not react under the abovementioned conditions; thus, its ester was prepared differently (see Supporting Information). The pure products were obtained after column chromatography on silica gel using a hexane:ethyl acetate 19:1 (v/v) mobile phase. Apart from the desired 6-bromo product, a 6-chloro byproduct was also obtained. The 6-bromo/chloro mixture (in an approximately 9:1 ratio according to NMR spectra) was used in the following synthetic step without further purification because the 6-chloro derivative reacts in the same manner as the 6-bromo analogue.

General procedure for the preparation of 6-(dimethylamino)hexanoic acid esters: First, 1 mmol of 6-bromohexanoyl ester was dissolved in 5 ml of tetrahydrofuran. Five equivalents of dimethyl amine (2 M solution in tetrahydrofuran) were added, and the reaction mixture was stirred at room temperature. The reactions were monitored by TLC on silica gel using an acetone: triethylamine 100:1 (v/v) mobile phase. After 24 h, the reaction mixture was dried in vacuo, dissolved in chloroform and subsequently washed with 0.1M HCl and water. The organic phase was collected and dried in vacuo, and the pure product was obtained after column chromatography on silica gel using an acetone: triethylamine 100:1 (v/v) mobile phase.

Characterization of the prepared compounds

3,7-dimethyloct-6-en-1-yl 6-bromohexanoate (**C-Br**): Yield 84 % as a colorless oil; M_w = 333.31. ^{1}H NMR (500 MHz, $CDCl_3$) δ 5.13 – 5.06 (m, 1H), 4.18 – 4.05 (m, 2H), 3.41 (t, *J* = 6.8 Hz, 2H), 2.32 (t, *J* = 7.4 Hz, 2H), 2.07 – 1.91 (m, 2H), 1.93 – 1.84 (m, 2H), 1.74 – 1.61 (m, 6H), 1.61 (d, *J* = 1.3 Hz, 3H), 1.59 – 1.51 (m, 1H), 1.54 – 1.38 (m, 3H), 1.41 – 1.29 (m,

1H), 1.24 – 1.14 (m, 1H), 0.92 (d, J = 6.6 Hz, 3H). ^{13}C NMR (126 MHz, $CDCl_3$) δ 173.49, 131.31, 124.52, 62.90, 36.95, 35.44, 34.10, 33.43, 32.38, 29.48, 27.64, 25.69, 25.36, 24.10, 19.39, 17.63. FTIR (ATR): ν_{max} 2929; 1734; 1541; 1457; 1254; 1174 cm^{-1}. MS (ESI, pos): m/z 355.3, 357.2 $[M+Na]^+$.

3,7-dimethyloct-6-en-1-yl 6-(dimethylamino)hexanoate (**C-DAK**): Yield 78 % as a yellowish oil; M_w = 297.48. ^1H NMR (500 MHz, $CDCl_3$) δ 5.13 – 5.04 (m, 1H), 4.16 – 4.03 (m, 2H), 2.30 (t, J = 7.5 Hz, 2H), 2.28 – 2.21 (m, 2H), 2.21 (s, 6H), 2.07 – 1.89 (m, 2H), 1.70 – 1.65 (m, 3H), 1.68 – 1.60 (m, 2H), 1.63 – 1.58 (m, 3H), 1.59 – 1.50 (m, 1H), 1.53 – 1.38 (m, 4H), 1.39 – 1.28 (m, 3H), 1.24 – 1.12 (m, 1H), 0.91 (d, J = 6.6 Hz, 3H). ^{13}C NMR (126 MHz, $CDCl_3$) δ 173.79, 131.27, 124.53, 62.79, 59.59, 45.42, 36.94, 35.44, 34.30, 29.47, 27.36, 27.00, 25.67, 25.36, 24.91, 19.38, 17.61. FTIR (ATR): ν_{max} 2930; 1737; 1461; 1378; 1173; 1042 cm^{-1}. MS (APCI, pos): m/z 298.1 $[M+H]^+$.

(*E*)-3,7-dimethylocta-2,6-dien-1-yl 6-bromohexanoate (**G-Br**): Yield 89 % as a colorless oil; M_w = 331.29. ^1H NMR (500 MHz, $CDCl_3$) δ 5.39 – 5.30 (m, 1H), 5.13 – 5.05 (m, 1H), 4.60 (dd, J = 7.1, 0.9 Hz, 2H), 3.41 (t, J = 6.8 Hz, 2H), 2.33 (t, J = 7.5 Hz, 2H), 2.16 – 2.01 (m, 4H), 1.93 – 1.84 (m, 2H), 1.71 (d, J = 1.3 Hz, 3H), 1.69 (d, 3H), 1.68 – 1.63 (m, 2H), 1.61 (d, J = 1.3 Hz, 3H), 1.52 – 1.45 (m, 2H). ^{13}C NMR (126 MHz, $CDCl_3$) δ 173.43, 142.23, 131.79, 123.71, 118.27, 61.28, 39.50, 34.08, 33.44, 32.38, 27.64, 26.28, 25.65, 24.10, 17.66, 16.45. FTIR (ATR): ν_{max} 2931; 1733; 1456; 1253; 1170; 974 cm^{-1}. MS (ESI, pos): m/z 353.3, 355.2 $[M+Na]^+$.

(*E*)-3,7-dimethylocta-2,6-dien-1-yl 6-(dimethylamino)hexanoate (**G-DAK**): Yield 60 % as a yellowish oil; M_w = 295.47. ^1H NMR (500 MHz, $CDCl_3$) δ 5.38 – 5.29 (m, 1H), 5.12 – 5.04 (m, 1H), 4.59 (d, J = 6.7 Hz, 2H), 2.31 (t, J = 7.5 Hz, 2H), 2.28 – 2.21 (m, 2H), 2.20 (s, 6H), 2.15 – 2.00 (m, 4H), 1.71 – 1.69 (m, 3H), 1.68 (d, J = 1.3 Hz, 3H), 1.67 – 1.61 (m, 2H), 1.61-1.59 (m, 3H), 1.51 – 1.44 (m, 2H), 1.37 – 1.30 (m, 2H). ^{13}C NMR (126 MHz, $CDCl_3$) δ 173.71, 142.08, 131.76, 123.72, 118.35, 61.18, 59.63, 45.47, 39.50, 34.28, 27.41, 27.01, 26.27, 25.64, 24.93, 17.64, 16.43. FTIR (ATR): ν_{max} 2937; 2762; 1734; 1456; 1169; 980 cm^{-1}. MS (ESI, pos): m/z 296.4 $[M+H]^+$, 318.3 $[M+Na]^+$.

(*Z*)-3,7-dimethylocta-2,6-dien-1-yl 6-bromohexanoate (**N-Br**): Yield 96 % as a colorless oil; M_w = 331.29. ^1H NMR (500 MHz, $CDCl_3$) δ 5.41 – 5.31 (m, 1H), 5.14 – 5.06 (m, 1H), 4.57 (dd, J = 7.3, 1.0 Hz, 2H), 3.41 (t, J = 6.8 Hz, 2H), 2.33 (t, J = 7.5 Hz, 2H), 2.17 – 2.03 (m, 4H), 1.91 – 1.84 (m, 2H), 1.80 – 1.75 (m, 3H), 1.70 – 1.69 (m, 3H), 1.69 – 1.63 (m, 2H), 1.61 (s, 3H), 1.52 – 1.44 (m, 2H). ^{13}C NMR (126 MHz, $CDCl_3$) δ 173.40, 142.58, 132.13, 123.54, 119.14, 60.99, 34.09, 33.43, 32.38, 32.15, 27.64, 26.64, 25.66, 24.09, 23.49, 17.64. FTIR (ATR): ν_{max} 2934; 2837; 1733; 1436; 1169; 889 cm^{-1}. MS (APCI, pos): m/z 353.1, 355.1 $[M+Na]^+$.

(*Z*)-3,7-dimethylocta-2,6-dien-1-yl 6-(dimethylamino)hexanoate (**N-DAK**): Yield 71 % as a yellowish oil; M_w = 295.47. ^1H NMR (500 MHz, $CDCl_3$) δ 5.39 – 5.31 (m, 1H), 5.13 – 5.05

(m, 1H), 4.56 (dd, *J* = 7.3, 1.0 Hz, 2H), 2.30 (t, *J* = 7.6 Hz, 2H), 2.28 – 2.21 (m, 2H), 2.21 (s, 6H), 2.15 – 2.03 (m, 4H), 1.76 (q, *J* = 1.1 Hz, 3H), 1.68 (d, *J* = 1.3 Hz, 3H), 1.67 – 1.62 (m, 2H), 1.60 (d, *J* = 1.3 Hz, 3H), 1.51 – 1.44 (m, 2H), 1.37 – 1.29 (m, 2H). ^{13}C NMR (126 MHz, $CDCl_3$) δ 173.68, 142.43, 132.09, 123.55, 119.21, 60.89, 59.62, 45.46, 34.28, 32.13, 27.40, 27.01, 26.62, 25.65, 24.92, 23.48, 17.62. FTIR (ATR): ν_{max} 2936; 2763; 1735; 1455; 1168; 978 cm^{-1}. MS (APCI, pos): m/z 296.3 $[M+H]^+$, 318.2 $[M+Na]^+$.

3,7,11-trimethyldodeca-2,6,10-trien-1-yl 6-bromohexanoate (**F-Br**): Mixture of *cis/trans* isomers, yield 93 % as a colorless oil; M_w = 399.41. ^{1}H NMR (500 MHz, $CDCl_3$) δ 5.39 – 5.31 (m, 1H), 5.16 – 5.06 (m, 2H), 4.60 (d, *J* = 7.1 Hz, 2H), 3.41 (t, *J* = 6.8 Hz, 2H), 2.37 – 2.29 (m, 2H), 2.18 – 1.95 (m, 9H), 1.93 – 1.83 (m, 2H), 1.73 – 1.71 (m, 3H), 1.70 – 1.68 (m, 5H), 1.68 – 1.63 (m, 1H), 1.63 – 1.62 (m, 1H), 1.62 – 1.60 (m, 3H), 1.52 – 1.45 (m, 2H). ^{13}C NMR (126 MHz, $CDCl_3$) δ 173.43, 142.25, 142.22, 135.57, 135.43, 131.56, 131.30, 124.40, 124.28, 124.25, 123.59, 118.29, 61.28, 39.80, 39.68, 39.51, 34.08, 33.44, 32.39, 31.96, 27.65, 26.70, 26.57, 26.18, 26.08, 25.71, 25.67, 24.10, 23.35, 17.67, 17.63, 16.46, 16.00. FTIR (ATR): ν_{max} 2929; 1733; 1443; 1380; 1170; 970 cm^{-1}. MS (ESI, pos): m/z 421.4, 423.3 $[M+Na]^+$.

3,7,11-trimethyldodeca-2,6,10-trien-1-yl 6-(dimethylamino)hexanoate (**F-DAK**): Mixture of *cis/trans* isomers, yield 72 % as a yellowish oil; M_w = 363.59. ^{1}H NMR (500 MHz, $CDCl_3$) δ 5.38 – 5.30 (m, 1H), 5.15 – 5.05 (m, 2H), 4.59 (d, *J* = 7.1 Hz, 2H), 2.35 – 2.27 (m, 2H), 2.28 – 2.21 (m, 2H), 2.21 (s, 6H), 2.16 – 1.94 (m, 9H), 1.79 – 1.58 (m, 13H), 1.51 – 1.44 (m, 2H), 1.38 – 1.30 (m, 2H). ^{13}C NMR (126 MHz, $CDCl_3$) δ 173.71, 142.09, 142.06, 135.53, 135.40, 131.53, 131.26, 124.40, 124.27, 124.24, 123.60, 118.37, 61.18, 59.63, 45.47, 39.79, 39.66, 39.50, 34.28, 31.94, 27.41, 27.02, 26.68, 26.57, 26.55, 26.18, 26.07, 25.69, 25.65, 24.93, 23.33, 17.65, 17.61, 16.44, 15.98. FTIR (ATR): ν_{max} 2935; 1737; 1444; 1382; 1170; 960 cm^{-1}. MS (APCI, pos): m/z 364.4 $[M+H]^+$, 386.2 $[M+Na]^+$.

3,7-dimethylocta-1,6-dien-3-yl 6-bromohexanoate (**L-Br**): 2 mmol of linalool were mixed with 1 equivalent of 6-bromohexanoic acid and dimethylaminopyridine (24 mg) in 5 ml of dry chloroform at 0°C. 1 equivalent of 1-ethyl-3-(3-dimethylaminopropyl)carbodiimide (WSC) was added dropwise and the reaction mixture was allowed to warm up to room temperature. After 20 h, the reaction mixture was subsequently extracted by saturated $NaHCO_3$ and water. The organic phase was dried and evaporated under reduced pressure. The product was obtained by column chromatography on silica gel using a mixture of hexane:ethyl acetate 19:1 (v/v) as a mobile phase. The reactions and column chromatography were monitored by TLC on silica gel using mobile phase hexane:ethyl acetate 9:1. Yield 21 % as a colorless oil; M_w = 331.29. ^{1}H NMR (500 MHz, $CDCl_3$) δ 5.98 (dd, *J* = 17.5, 11.0 Hz, 1H), 5.18 – 5.07 (m, 3H), 3.41 (t, *J* = 6.8 Hz, 2H), 2.29 (t, *J* = 7.4 Hz, 2H), 2.01 – 1.93 (m, 2H), 1.92 – 1.73 (m, 4H), 1.68 (d, *J* = 1.3 Hz, 3H), 1.67 – 1.61 (m, 2H), 1.60 (d, *J* = 1.3 Hz, 3H), 1.55 (s, 3H), 1.52 – 1.45 (m, 2H). ^{13}C NMR (126 MHz, $CDCl_3$) δ 172.21, 141.83, 131.79, 123.77, 113.09, 82.83, 39.79, 35.05, 33.48, 32.44, 27.65, 25.64, 24.17, 23.60, 22.36, 17.57. FTIR (ATR): ν_{max} 2933; 1732; 1454; 1374; 1165; 922 cm^{-1}. MS (ESI, pos): m/z 353.1, 355.3 $[M+Na]^+$.

3,7-dimethylocta-1,6-dien-3-yl 6-(dimethylamino)hexanoate (**L-DAK**): Yield 71 % as a yellowish oil; M_w = 295.47. ^{1}H NMR (500 MHz, $CDCl_3$) δ 5.96 (dd, *J* = 17.5, 11.0 Hz, 1H), 5.14 (dd, *J* = 17.5, 1.0 Hz, 1H), 5.11 (dd, *J* = 11.0, 1.0 Hz, 1H), 5.11 – 5.06 (m, 1H), 2.30 – 2.22 (m, 4H), 2.22 (s, 6H), 2.01 – 1.92 (m, 2H), 1.90 – 1.80 (m, 1H), 1.80 – 1.70 (m, 1H), 1.67 (d, *J* = 1.3 Hz, 3H), 1.67 – 1.58 (m, 2H), 1.59 (d, *J* = 1.2 Hz, 3H), 1.53 (s, 3H), 1.52 – 1.45 (m, 2H), 1.37 – 1.30 (m, 2H). ^{13}C NMR (126 MHz, $CDCl_3$) δ 172.48, 141.91, 131.73, 123.79, 112.97, 82.64, 59.59, 45.40, 39.78, 35.24, 27.35, 26.97, 25.62, 24.97, 23.61, 22.33, 17.55. FTIR (ATR): ν_{max} 2936; 1733; 1456; 1374; 1165; 921 cm^{-1}. MS (APCI, pos): m/z 296.0 $[M+H]^+$.

(*S*)-(4-(prop-1-en-2-yl)cyclohex-1-en-1-yl)methyl 6-bromohexanoate (**P-Br**): Yield 94 % as a colorless oil; M_w = 329.28; $[\alpha]_D^{28.0°C}$ -36.50 (0.01 g/ml, $CHCl_3$). ^{1}H NMR (500 MHz, $CDCl_3$) δ 5.79 – 5.73 (m, 1H), 4.77 – 4.70 (m, 2H), 4.48 (s, 2H), 3.41 (t, *J* = 6.8 Hz, 2H), 2.36 (t, *J* = 7.5 Hz, 2H), 2.22 – 2.11 (m, 2H), 2.13 – 2.05 (m, 2H), 2.04 – 1.94 (m, 1H), 1.94 – 1.84 (m, 3H), 1.77 – 1.72 (m, 3H), 1.73 – 1.63 (m, 2H), 1.55 – 1.45 (m, 3H). ^{13}C NMR (126 MHz, $CDCl_3$) δ 173.31, 149.53, 132.61, 125.77, 108.76, 68.35, 40.79, 34.06, 33.44, 32.38, 30.44, 27.66, 27.28, 26.39, 24.11, 20.73. FTIR (ATR): ν_{max} 2934; 1733; 1436; 1254; 1169; 889 cm^{-1}. MS (ESI, pos): m/z 351.2, 353.1 $[M+Na]^+$.

(*S*)-(4-(prop-1-en-2-yl)cyclohex-1-en-1-yl)methyl 6-(dimethylamino)hexanoate (**P-DAK**): Yield 58 % as a yellowish oil; M_w = 293.45; $[\alpha]_D^{28.0°C}$ -38.00 (0.01 g/ml, $CHCl_3$). ^{1}H NMR (500 MHz, $CDCl_3$) δ 5.78 – 5.72 (m, 1H), 4.77 – 4.68 (m, 2H), 4.46 (s, 2H), 2.33 (t, *J* = 7.5 Hz, 2H), 2.28 – 2.21 (m, 2H), 2.21 (s, 6H), 2.20 – 2.10 (m, 2H), 2.11 – 2.04 (m, 2H), 2.03 – 1.91 (m, 1H), 1.89 – 1.81 (m, 1H), 1.74 (s, 3H), 1.69 – 1.62 (m, 2H), 1.54 – 1.44 (m, 3H), 1.38 – 1.30 (m, 2H). ^{13}C NMR (126 MHz, $CDCl_3$) δ 173.58, 149.54, 132.67, 125.62, 108.73, 68.23, 59.63, 45.48, 40.80, 34.26, 30.43, 27.41, 27.28, 27.01, 26.37, 24.93, 20.71. FTIR (ATR): ν_{max} 2936; 1734; 1457; 1374; 1169; 888 cm^{-1}. MS (APCI, pos): m/z 294.3 $[M+H]^+$, 316.3 $[M+Na]^+$.

(1*R*,2*S*,5*R*)-2-isopropyl-5-methylcyclohexyl 6-bromohexanoate (**M-Br**): Yield 34.4 % as a colorless oil; M_w = 333.31; $[\alpha]_D^{27.7°C}$ -40.67 (0.01 g/ml, $CHCl_3$). ^{1}H NMR (500 MHz, $CDCl_3$) δ 4.69 (td, *J* = 10.8, 4.4 Hz, 1H), 3.41 (t, *J* = 6.8 Hz, 2H), 2.31 (t, *J* = 7.4 Hz, 2H), 2.02 – 1.95 (m, 1H), 1.94 – 1.82 (m, 3H), 1.73 – 1.60 (m, 4H), 1.54 – 1.43 (m, 3H), 1.41 – 1.33 (m, 1H), 1.11 – 0.92 (m, 3H), 0.91 (d, *J* = 3.1 Hz, 3H), 0.90 (d, *J* = 3.6 Hz, 3H), 0.77 (d, *J* = 7.0 Hz, 3H). ^{13}C NMR (126 MHz, $CDCl_3$) δ 172.99, 74.06, 47.01, 40.95, 34.45, 34.25, 33.47, 32.41, 31.37, 27.65, 26.28, 24.23, 23.42, 22.00, 20.75, 16.30. FTIR (ATR): ν_{max} 2954; 2868; 1730; 1455; 1253; 1180; 985 cm^{-1}. MS (ESI, pos): m/z 355.2, 357.2 $[M+Na]^+$.

(1*R*,2*S*,5*R*)-2-isopropyl-5-methylcyclohexyl 6-(dimethylamino)hexanoate (**M-DAK**): Yield 77 % as a yellowish oil; M_w = 297.48; $[\alpha]_D^{27.8°C}$ -40.00 (0.01 g/ml, $CHCl_3$). ^{1}H NMR (300 MHz, $CDCl_3$) δ 4.64 (td, *J* = 10.9, 4.4 Hz, 1H), 3.01 – 2.89 (m, 2H), 2.77 (s, 6H), 2.28 (t, *J* = 7.3 Hz, 2H), 1.99 – 1.74 (m, 4H), 1.73 – 1.57 (m, 4H), 1.50 – 1.27 (m, 4H), 1.10 – 0.90 (m, 2H), 0.89 (d, *J* = 2.7 Hz, 3H), 0.86 (d, *J* = 3.2 Hz, 3H), 0.84 – 0.78 (m, 1H), 0.72 (d, *J* = 7.0 Hz, 3H). ^{13}C NMR (75 MHz, $CDCl_3$) δ 172.74, 74.17, 57.72, 46.88, 42.79, 40.88, 34.14, 34.08, 31.32, 26.22, 26.05, 24.23, 23.96, 23.34, 21.96, 20.69, 16.26. FTIR (ATR): ν_{max} 2948; 2480; 1726; 1469; 1250; 1182; 965 cm^{-1}. MS (APCI, pos): m/z 298.1 $[M+H]^+$.

(1*S*,4*S*)-1,7,7-trimethylbicyclo[2.2.1]heptan-2-yl 6-bromohexanoate (**B-Br**): predominantly *endo* (2*R*) isomer, yield 32 % as a colorless oil; M_w = 331.29; $[\alpha]_D^{27.4°C}$ -10.00 (0.01 g/ml, $CHCl_3$). ^1H NMR (500 MHz, $CDCl_3$) δ 4.99 – 4.80 (m, 0.7H, COOC$\underline{H}$), 4.74 – 4.58 (m, 0.3H, COOC$\underline{H}$), 3.45 – 3.36 (m, 2H), 2.47 – 2.21 (m, 2H), 2.07 – 1.81 (m, 3H), 1.79 – 1.70 (m, 2H), 1.70 – 1.54 (m, 3H), 1.54 – 1.44 (m, 2H), 1.37 – 1.03 (m, 3H), 0.99 – 0.94 (m, 1.5H), 0.91 (s, 2H), 0.88 (s, 2H), 0.84 (s, 1.5H), 0.83 (s, 2H).^{13}C NMR (126 MHz, $CDCl_3$) δ 173.70, 172.88, 80.86, 79.75, 48.72, 48.61, 47.76, 46.89, 45.01, 44.88, 38.82, 36.82, 34.50, 34.41, 33.73, 33.46, 33.44, 32.42, 28.03, 27.68, 27.66, 27.11, 27.00, 24.24, 24.18, 20.09, 19.88, 19.68, 18.82, 13.50, 11.44. FTIR (ATR): ν_{max} 2953; 2358; 1731; 1456; 1254; 1185; 1020 cm^{-1}. MS (ESI, pos): m/z 353.2, 355.2 $[M+Na]^+$.

(1*S*,4*S*)-1,7,7-trimethylbicyclo[2.2.1]heptan-2-yl 6-(dimethylamino)hexanoate (**B-DAK**): predominantly *endo* (2*R*) isomer, yield 68 % as a yellowish oil; M_w = 295.47; $[\alpha]_D^{27.5°C}$ -12.67 (0.01 g/ml, $CHCl_3$). ^1H NMR (500 MHz, $CDCl_3$) δ 4.90 – 4.84 (m, 0.7H, COOC$\underline{H}$), 4.67 – 4.61 (m, 0.3H, COOC$\underline{H}$), 2.31 (t, J = 7.5 Hz, 2H), 2.30 – 2.20 (m, 2H), 2.22 – 2.18 (m, 6H), 1.95 – 1.89 (m, 1H), 1.82 – 1.68 (m, 2H), 1.68 – 1.58 (m, 3H), 1.54 – 1.44 (m, 2H), 1.38 – 1.31 (m, 2H), 1.31 – 1.03 (m, 3H), 0.97 – 0.91 (m, 1.5H), 0.89 (s, 2H), 0.85 (s, 2H), 0.82 (s, 1.5H), 0.81 (s, 2H).^{13}C NMR (126 MHz, $CDCl_3$) δ 173.98, 173.15, 80.70, 79.58, 59.59, 59.57, 48.69, 48.57, 47.73, 46.87, 45.42, 44.99, 44.86, 38.81, 36.80, 34.69, 34.58, 33.71, 28.01, 27.34, 27.08, 27.00, 26.97, 25.03, 24.95, 20.08, 19.86, 19.67, 18.81, 13.48, 11.41. FTIR (ATR): ν_{max} 2949; 1732; 1454; 1244; 1178; 1025 cm^{-1}. MS (APCI, pos): m/z 296.1 $[M+H]^+$.

(5*R*)-2-methyl-5-(prop-1-en-2-yl)cyclohex-2-en-1-yl 6-bromohexanoate (**Ca-Br**): yield 42 % as a colorless oil; M_w = 329.28; $[\alpha]_D^{27.9°C}$ -51.33 (0.01 g/ml, $CHCl_3$). ^1H NMR (500 MHz, $CDCl_3$) δ 5.78 – 5.72 (m, 0.4H, COOC$\underline{H}$), 5.65 – 5.58 (m, 0.6H, COOC$\underline{H}$), 5.51 – 5.43 (m, 0.6H, =C$\underline{H}$-), 5.33 – 5.22 (m, 0.4H, =C$\underline{H}$-), 4.78 – 4.69 (m, 2H), 3.45 – 3.37 (m, 2H), 2.40 – 2.33 (m, 2H), 2.33 – 2.27 (m, 1H), 2.25 – 2.15 (m, 1H), 2.16 – 2.05 (m, 1H), 2.02 – 1.94 (m, 1H), 1.94 – 1.76 (m, 3H), 1.76 – 1.71 (m, 3H), 1.71 – 1.67 (m, 3H), 1.68 – 1.60 (m, 2H), 1.55 – 1.44 (m, 2H). ^{13}C NMR (126 MHz, $CDCl_3$) δ 173.22, 148.67, 148.25, 132.81, 130.89, 127.87, 125.93, 109.31, 109.19, 73.09, 70.54, 40.26, 35.87, 34.45, 34.39, 34.36, 34.02, 33.73, 33.43, 33.39, 32.40, 32.37, 30.85, 30.73, 27.66, 27.64, 24.31, 24.20, 20.80, 20.62, 20.46, 18.84. FTIR (ATR): ν_{max} 2939; 1735; 1453; 1251; 1184; 890 cm^{-1}. MS (ESI, pos): m/z 351.2, 353.2 $[M+Na]^+$.

(5*R*)-2-methyl-5-(prop-1-en-2-yl)cyclohex-2-en-1-yl 6-(dimethylamino)hexanoate (**Ca-DAK**): yield 51 % as a yellowish oil; M_w = 293.45; $[\alpha]_D^{27.8°C}$ -58.00 (0.01 g/ml, $CHCl_3$). ^1H NMR (500 MHz, $CDCl_3$) δ 5.77 – 5.70 (m, 0.4H, COOC$\underline{H}$), 5.63 – 5.57 (m, 0.6H, COOC$\underline{H}$), 5.50 – 5.42 (m, 0.6H, =C$\underline{H}$-), 5.29 – 5.24 (m, 0.4H, =C$\underline{H}$-), 4.77 – 4.68 (m, 2H), 2.37 – 2.29 (m, 2H), 2.28 – 2.21 (m, 2H), 2.21 (s, 3H), 2.20 (s, 3H), 2.22 – 2.14 (m, 1H), 2.15 – 2.03 (m, 1H), 2.02 – 1.93 (m, 1H), 1.93 – 1.81 (m, 1H), 1.73 – 1.71 (m, 3H), 1.69 – 1.67 (m, 2H), 1.67 – 1.64 (m, 1H), 1.64 – 1.62 (m, 2H), 1.52 – 1.43 (m, 3H), 1.39 – 1.31 (m, 2H). ^{13}C NMR (126 MHz, $CDCl_3$) δ 173.50, 148.69, 148.28, 132.91, 130.97, 127.76, 125.83, 109.28, 109.15, 72.93, 70.39, 59.61, 45.45, 40.26, 35.86, 34.66, 34.56, 34.01, 33.74, 30.84, 30.73, 27.40, 27.39, 27.02,

26.99, 25.14, 25.02, 20.80, 20.60, 20.46, 18.81. FTIR (ATR): ν_{max} 2938; 1731; 1453; 1374; 1169; 889 cm^{-1}. MS (ESI, pos): m/z 294.4 $[M+H]^+$, 316.3 $[M+Na]^+$.

(*E*)-3-phenylprop-2-en-1-yl 6-bromohexanoate (**Ci-Br**): Yield 82 % as a colorless oil; M_w = 311.22. ^1H NMR (500 MHz, $CDCl_3$) δ 7.44 – 7.37 (m, 2H), 7.37 – 7.30 (m, 2H), 7.31 – 7.24 (m, 1H), 6.67 (d, *J* = 15.9 Hz, 1H), 6.30 (dt, *J* = 15.9, 6.5 Hz, 1H), 4.75 (dd, *J* = 6.5, 1.4 Hz, 2H), 3.42 (t, *J* = 6.7 Hz, 2H), 2.39 (t, *J* = 7.4 Hz, 2H), 1.93 – 1.78 (m,2H), 1.74 – 1.66 (m, 2H), 1.55 – 1.47 (m, 2H). ^{13}C NMR (126 MHz, $CDCl_3$) δ 173.15, 136.17, 134.20, 128.57, 128.05, 126.58, 123.18, 64.95, 34.06, 33.43, 32.36, 27.63, 24.19. FTIR (ATR): ν_{max} 2941; 1732; 1449; 1254; 1168; 745; 693 cm^{-1}. MS (ESI, pos): m/z 333.2, 335.1 $[M+Na]^+$.

(*E)*-3-phenylprop-2-en-1-yl 6-(dimethylamino)hexanoate (**Ci-DAK**): Yield 83 % as a yellowish oil; M_w = 275.39. ^1H NMR (300 MHz, $CDCl_3$) δ 7.44 – 7.19 (m, 5H), 6.64 (d, *J* = 15.9 Hz, 1H), 6.28 (dt, *J* = 15.9, 6.4 Hz, 1H), 4.72 (dd, *J* = 6.4, 1.3 Hz, 2H), 2.36 (t, *J* = 7.5 Hz, 2H), 2.30 – 2.16 (m, 8H), 1.73 – 1.61 (m, 2H), 1.54 – 1.42 (m, 2H), 1.41 – 1.28 (m, 2H). ^{13}C NMR (75 MHz, $CDCl_3$) δ 173.43, 136.17, 134.05, 128.54, 127.99, 126.54, 123.24, 64.85, 59.56, 45.42, 34.21, 27.35, 26.98, 24.87. FTIR (ATR): ν_{max} 2939; 1735; 1450; 1167; 966; 746; 693 cm^{-1}. MS (APCI, pos): m/z 276.7 $[M+H]^+$.

Drug solubility in donor samples

To determine the effects of the studied compounds on the drug solubility in the donor solvent C_0, the equilibrated donor samples were centrifuged at 6700 × g for 10 min. The supernatant was withdrawn and diluted with pH 7.4 phosphate-buffered saline (PBS). The concentrations of model drugs in the samples with and without enhancers were analyzed by HPLC.

Permeation experiments

The effects of the prepared compounds on the skin permeability was studied *in vitro* using Franz diffusion cells. Frozen human skin was slowly thawed, examined for any potential damage and cut into approximately 2 cm^2 squares. Skin fragments were sandwiched between two Teflon holders with a permeation area of 1 cm^2 and sealed with silicone grease. The skin samples in the holders were mounted into the Franz cells dermal side down and the acceptor compartments were filled with PBS (containing 0.005% gentamicine as a preservative). The exact acceptor volume of each cell was measured (15.7 ± 0.1 ml for TH and HC; 6.8 ± 0.1 ml for CDV) and used in the calculation of drug flux. The cells were allowed to equilibrate at 32°C for 1 h and the skin integrity was checked using electrical impedance (see later).

Then, an infinite dose (150 µl) of the respective donor sample was applied on the skin surface and the donor compartment of each cell was covered with glass slide to prevent the sample evaporation. The acceptor was stirred at 32°C throughout all experiments. The sink conditions were maintained during the experiment because the solubilities of TH, HC and CDV in the acceptor were 7.65 ± 0.02 mg/ml, 0.28 ± 0.004 mg/ml, and over 100 mg/ml, respectively. The experiment was conducted for 48, 64, and 72 h for TH, HC and CDV, respectively, to reach

steady-state conditions.[1] The samples of acceptor phase (300 µl) were withdrawn in predetermined time intervals and replaced by the same volume of fresh PBS.

High-Performance Liquid Chromatography (HPLC)

Concentration of TH, HC, and CDV in all samples were determined by a Shimadzu Prominence instrument (Shimadzu, Japan) consisting of LC-20AD pumps with a DGU-20A3 degasser, SIL-20A HT autosampler, CTO-20AC column oven, SPD-M20A diode array detector, CBM-20A communication module, and LCsolutions 1.22 software.

TH samples were analyzed on a LiChroCART 250-4 column (LiChrospher 100 RP-18, 5 µm, Merck, Germany) at 35°C, using a mobile phase consisting of 40% methanol in 0.1 M NaH_2PO_4 (v/v) at a flow rate of 1.2 ml/min, and detected at 272 nm. The calibration curve was linear in the 0.5-100 µg/ml range ($r^2 \geq 0.999$, $p<0.001$).[2]

HC was determined on a Discovery® HS C18 HPLC column (150-4.6 mm, 5 µm particles, Supelco, USA) at 40°C. 60% methanol with 1% tetrahydrofuran (v/v) at 1.2 ml/min was used as a mobile phase and HC concentration was measured at 242 nm. The calibration curve was linear in the range of 0.5–100 µg/ml ($r^2 \geq 0.999$, $p < 0.001$).[3, 4] Notably, cinnamyl alcohol and its derivative Ci-DAK absorb UV at 242 nm and have similar retention to HC using the conditions described above. Thus, the analysis of the samples with HC and cinnamyl derivatives was modified as follows: A LiChroCART 250-4 column (LiChrospher 100 RP-18, 5 µm, Merck, Germany) at 40°C, with a mobile phase consisting of 40% acetonitrile in 0.5% acetic acid (v/v) at a flow rate of 1.5 ml/min and detection at 242 nm. The calibration curve was linear in the range of 0.5-100 µg/ml ($r^2 \geq 0.999$, $p<0.001$).

CDV was quantified in HILIC mode using a SeQuantTM ZIC®-HILIC 150-4.6 mm column with 5 µm particles with a porosity of 200 Å (Merck, Germany) at 30°C. Mobile phase consisted of 75% acetonitrile in 50 mM ammonium acetate (v/v) and was used at a flow rate of 2 ml/min; CDV was monitored at 274 nm. The calibration curve was linear over the range 0.1 – 100 µg/ml ($r^2 \geq 0.999$, $p < 0.001$).[5]

Cellular toxicities of enhancers

Both the 3T3 and HaCaT cell lines were cultivated in Dulbecco's modified Eagle's medium (DMEM, Lonza, Belgium) supplemented with 10% heat-inactivated fetal bovine serum (Sigma, Germany), 10 mM HEPES buffer (Sigma, Germany) and 1% penicillin/streptomycin solution (Lonza, Belgium) in 75 cm^2 tissue culture flasks (TPP, Switzerland) at 37°C in a humidified atmosphere of 5% CO_2. Sub-confluent cells were sub-cultured every 3-4 days. For toxicity studies and confocal microscopy, cells (passage 15 to 25) were seeded at a density of 10,000 cells per well (in 100 µl of cell culture medium) into 96-well plates (TPP) and 100,000 cells per well (in 500 µl of cell-culture medium) into 4-well cell-imaging slide (Eppendorf, Germany), respectively. Seeded cells were left to grow for 24 h before addition of studied enhancers.

The stock samples for toxicity studies were prepared as solutions of the studied enhancers in dimethyl sulfoxide (150 mM stock solution) and then diluted to the required concentrations (1 – 800 μM) by the cell-culture medium immediately before the application on the cells. Total amount of dimethyl sulfoxide in each sample was kept well under the limit of its intrinsic measurable toxicity (0.2 vol. % for most of the experiments and under 0.6 vol. % for Ci-DAK on HaCaT cells). Control experiments without enhancers and DMSO (100 % viability), control experiments with DMSO and experiments with cells killed by lethal dose of H_2O_2 (0 % viability) were conducted as well. The cell viability was determined using 3-(4,5-dimethylthiazol-2-yl)-2,5-diphenyltetrazolium bromide (MTT) uptake assay after 48 h incubation with the enhancer. This method is based on the transformation of yellow MTT into purple formazan by mitochondrial reductases in living cells. MTT was dissolved in PBS (3 mg/ml), 25 μl of the prepared solution was added into each well and incubated at 37°C for 2 h. The medium with MTT was removed and the cells were lysed using 100 μl of dimethyl sulfoxide per well and 12 h vigorous shaking. After lysis, the cell viability was assessed by optical density measurement using a Tecan Infinite 200 M plate reader (Tecan, Austria) at λ = 570 nm and 690 nm.

Laser scanning confocal microscopy

Morphological changes were evaluated using laser scanning confocal microscopy after 48 h incubation of the cells with enhancers under the above-mentioned conditions using concentrations of compounds corresponding to their IC_{15} and IC_{85}. After incubation, cells were rinsed twice with warm PBS and fixative solution (4% paraformaldehyde in cell-culture medium) was added for 15 min. After fixation, the samples were washed three times with PBS, permeabilized for 15 min (0.5% Triton ×100) at room temperature, washed again three times and blocked using 3% bovine serum albumin in PBS for 60 min. Staining for actin and tubulin cytoskeleton was performed for 90 min using 5 U/mL Alexa Fluor 555 phalloidin (Molecular Probes) and 2 μg/mL α-tubulin antibody, Alexa Fluor 488 conjugate (Thermo Fisher Scientific), respectively. Specimens were rinsed twice with PBS and stained with 4 nM Hoechst 33342 in Dulbecco´s modified PBS for 10 min. Removing excess salts from specimens was performed by washing with demineralized water for 5 min. Dry samples were mounted on glass slide with ProLong Gold antifade mountant (Molecular Probes) overnight at rt and sealed using colorless nail polish. Photomicrographs were taken using Nikon A1+ confocal system (Nikon, Japan).

Skin electrical impedance

Skin electrical impedance was measured with an LCR meter 4080 (Conrad electronic, Germany, measuring range 20 Ω – 10 MΩ, error at kΩ values < 0.5 %) operated in parallel mode with an alternating frequency of 120 Hz, which was used in our previous work.[5, 6] First, the volume of 0.5 ml of PBS was applied on the skin surface. After 20 min equilibration period, electrical impedance was recorded with two stainless steel electrodes immersed in

PBS in the donor and acceptor compartments of the Franz cell. PBS was removed with a cotton swab and the skin surface was blotted dry after each impedance measurement.

Transepidermal water loss (TEWL)

The reversibility of the effects of the studied compounds on the skin water loss was measured using a Tewameter® TM 300 probe and Multi-Probe Adapter Cutometer® MPA 580 (CK electronic GmbH, Germany). The upper part of the Franz cell was removed and the Tewameter probe was placed onto the Teflon holder. Measurements were conducted at air temperature 24-28°C and relative air humidity 25-43%. For details, see our previous studies.[5, 6]

References

(1) Janůšová, B., Školová, B., Tükörová, K., Wojnarová, L., Šimůnek, T., Mladěnka, P., Filipský, T., Říha, M., Roh, J., and Palát, K. (2013) Amino acid derivatives as transdermal permeation enhancers. *J Control Release 165*, 91-100.

(2) Novotný, J., Janůšová, B., Novotný, M., Hrabálek, A., and Vávrová, K. (2008) Short-chain ceramides decrease skin barrier properties. *Skin Pharmacol Physiol 22*, 22-30.

(3) Novotný, J., Kovaříková, P., Novotný, M., Janůšová, B., Hrabálek, A., and Vávrová, K. (2009) Dimethylamino acid esters as biodegradable and reversible transdermal permeation enhancers: effects of linking chain length, chirality and polyfluorination. *Pharm Res 26*, 811-821.

(4) Novotný, M., Klimentová, J., Janůšová, B., Palát, K., Hrabálek, A., and Vávrová, K. (2011) Ammonium carbamates as highly active transdermal permeation enhancers with a dual mechanism of action. *J Control Release 150*, 164-170.

(5) Kopečná, M., Macháček, M., Prchalová, E., Štěpánek, P., Drašar, P., Kotora, M., and Vávrová, K. (2017) Dodecyl Amino Glucoside Enhances Transdermal and Topical Drug Delivery via Reversible Interaction with Skin Barrier Lipids. *Pharm Res 34*, 640-653.

(6) Kopečná, M., Macháček, M., Prchalová, E., Štěpánek, P., Drašar, P., Kotora, M., and Vávrová, K. (2017) Galactosyl Pentadecene Reversibly Enhances Transdermal and Topical Drug Delivery. *Pharm Res 34*, 2097-2108.

3,7-dimethyloct-6-en-1-yl 6-bromohexanoate (**C-Br**)

^{1}H NMR (500 MHz, $CDCl_3$):

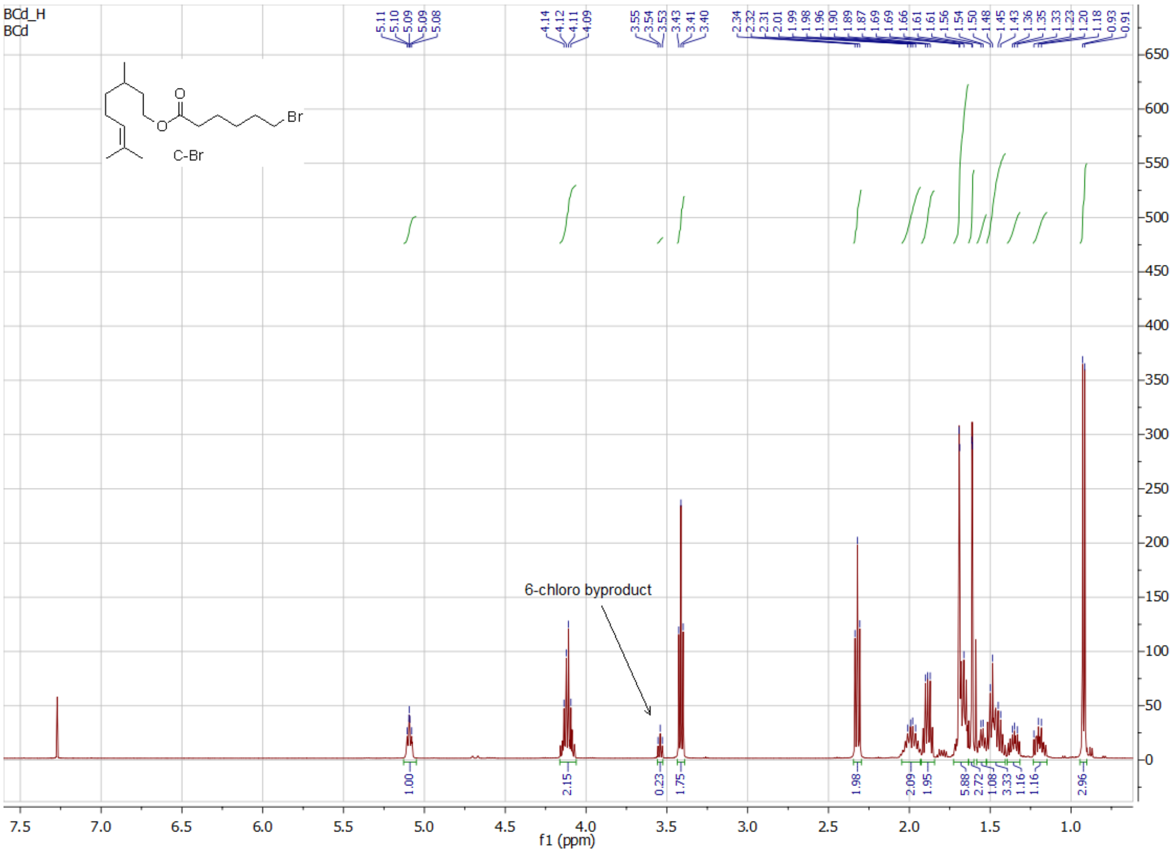

^{13}C NMR (126 MHz, $CDCl_3$):

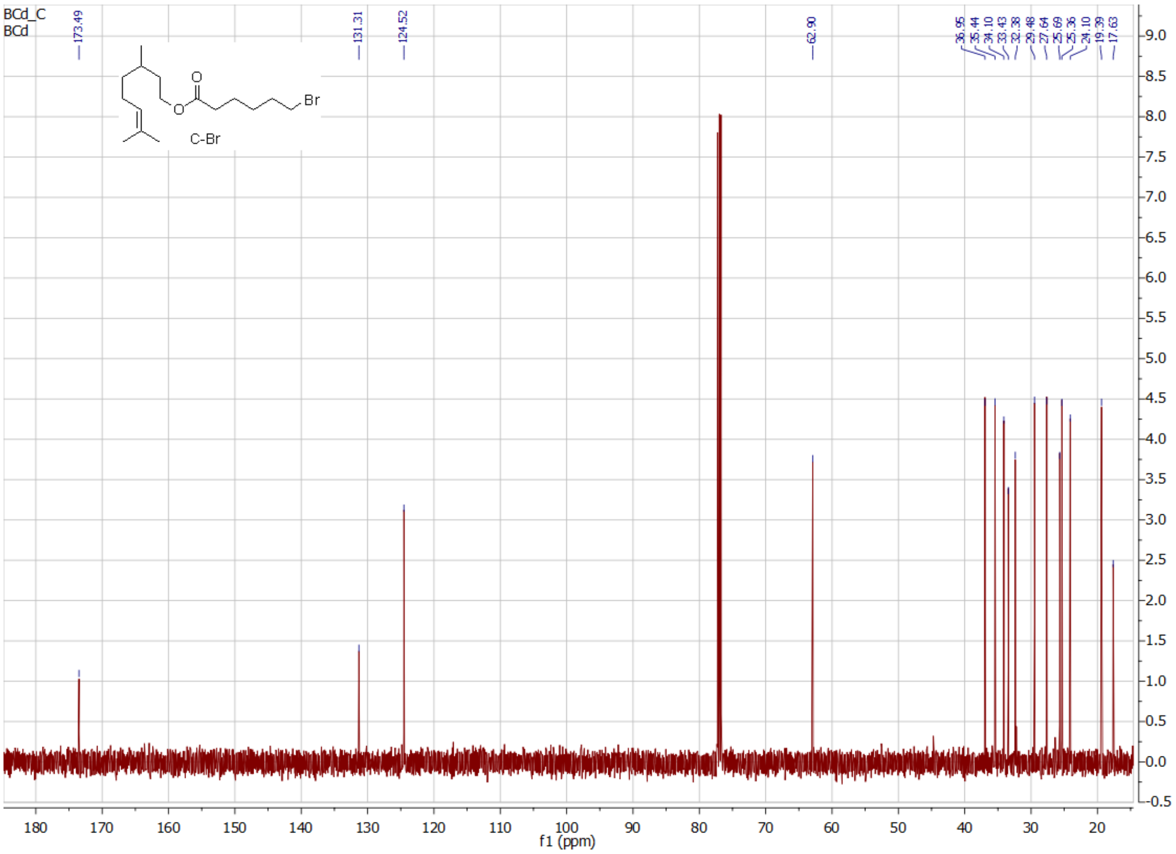

3,7-dimethyloct-6-en-1-yl 6-(dimethylamino)hexanoate (**C-DAK**)

^{1}H NMR (500 MHz, $CDCl_3$):

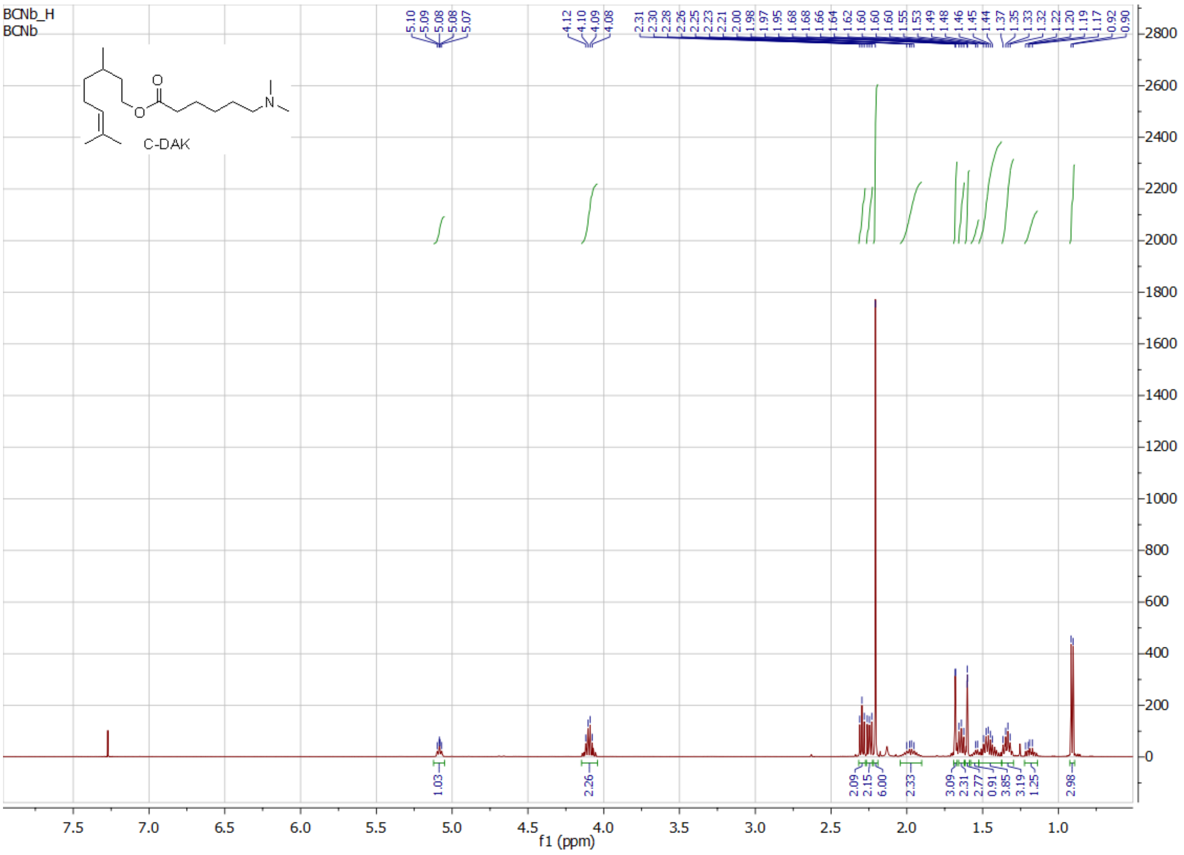

^{13}C NMR (126 MHz, $CDCl_3$):

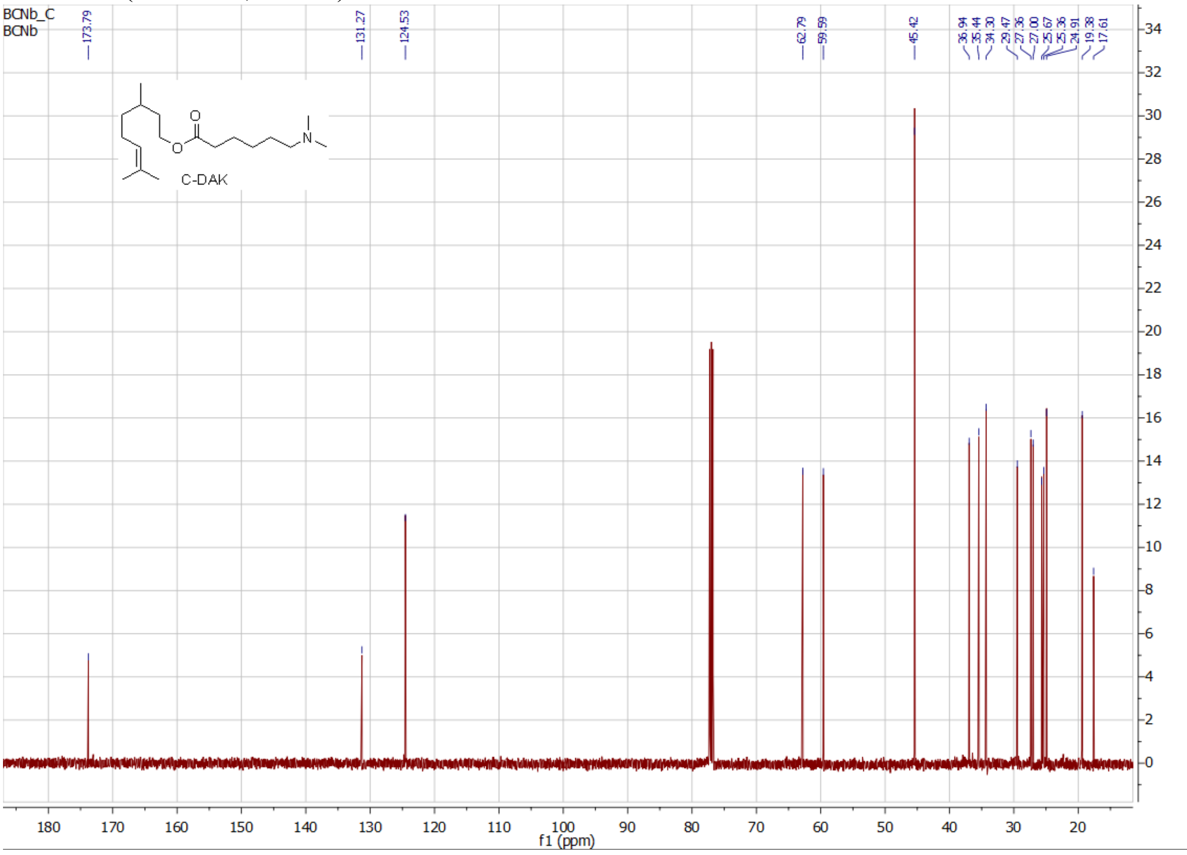

(*E*)-3,7-dimethylocta-2,6-dien-1-yl 6-bromohexanoate (**G-Br**)

^{1}H NMR (500 MHz, $CDCl_3$):

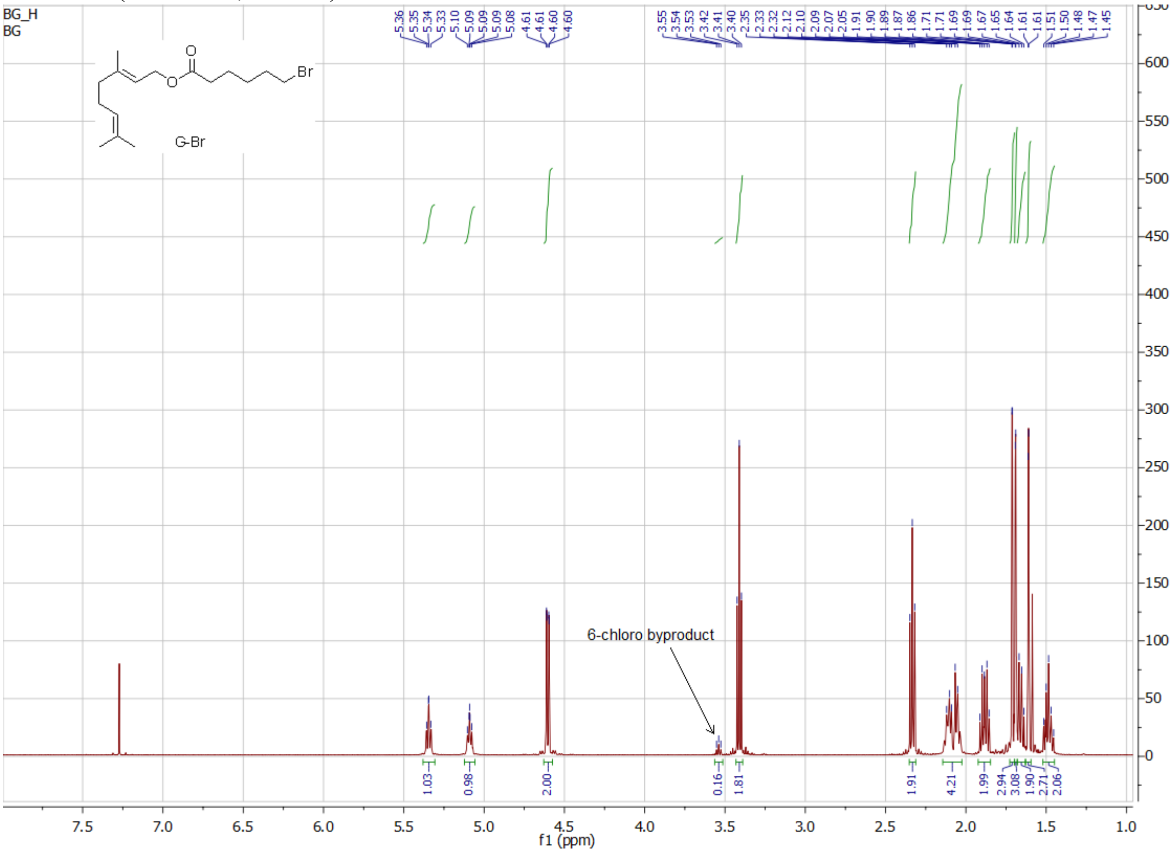

^{13}C NMR (126 MHz, $CDCl_3$):

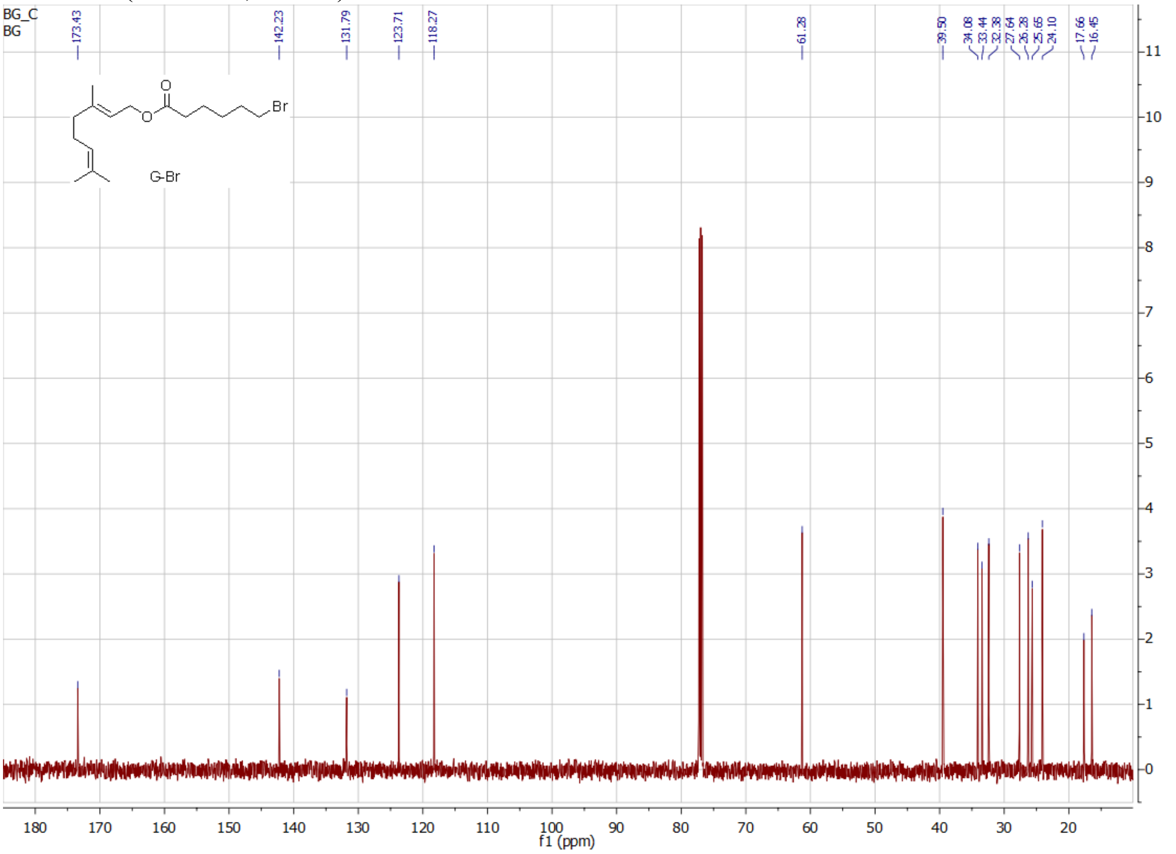

(*E*)-3,7-dimethylocta-2,6-dien-1-yl 6-(dimethylamino)hexanoate (**G-DAK**)

^{1}H NMR (500 MHz, $CDCl_3$):

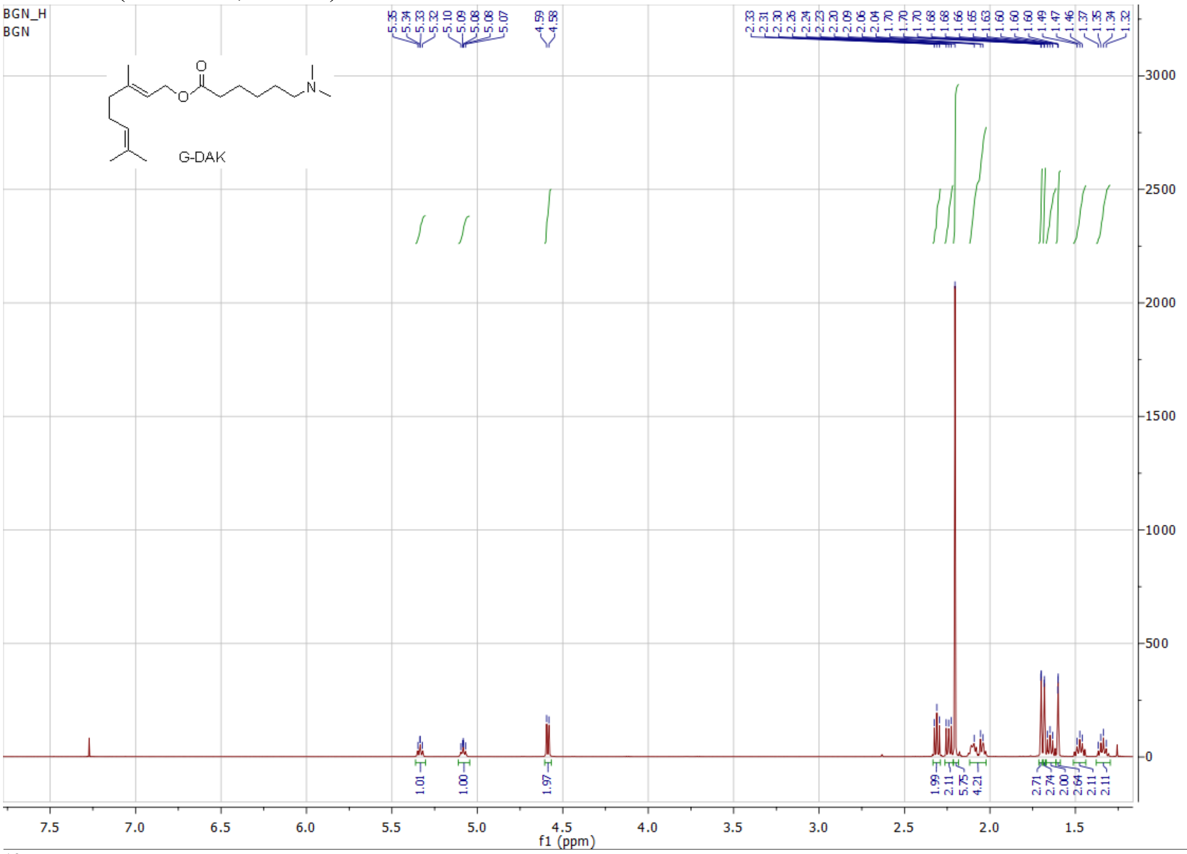

^{13}C NMR (126 MHz, $CDCl_3$):

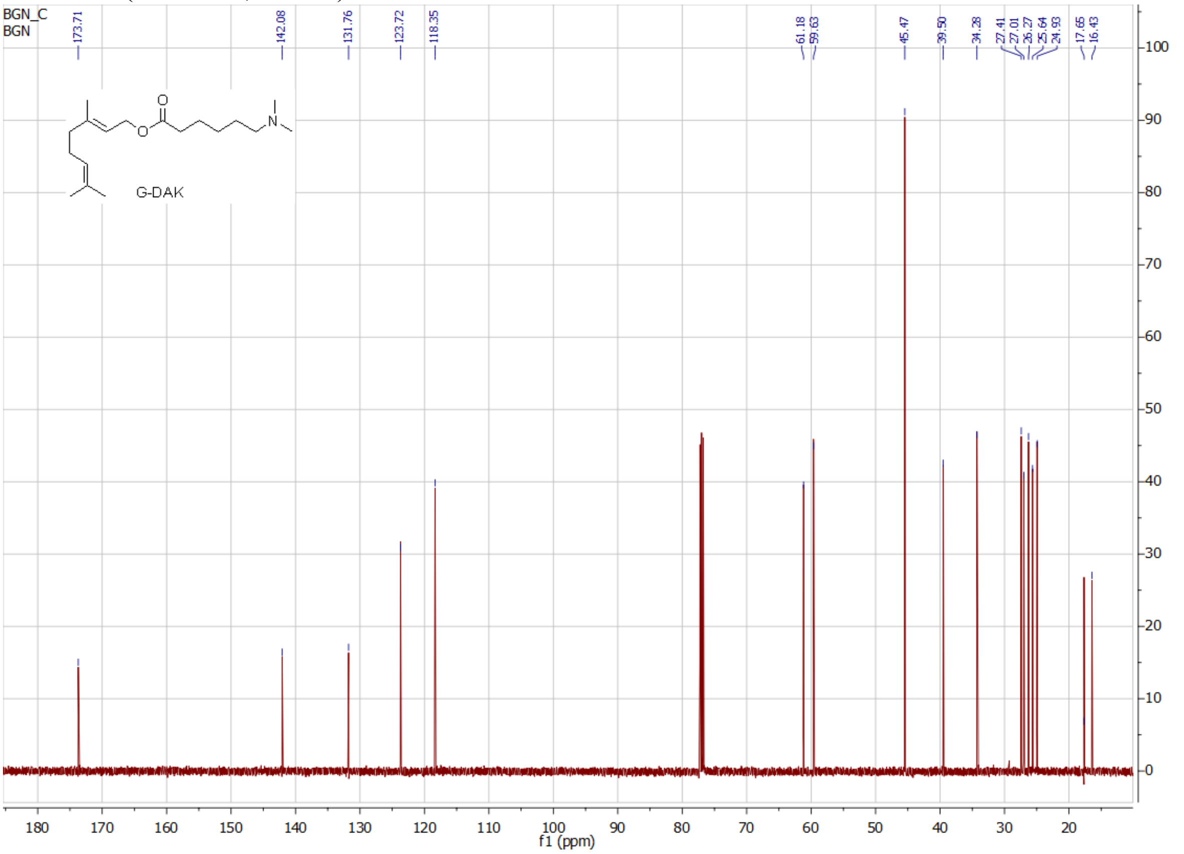

(Z)-3,7-dimethylocta-2,6-dien-1-yl 6-bromohexanoate (**N-Br**):

^{1}H NMR (500 MHz, $CDCl_3$):

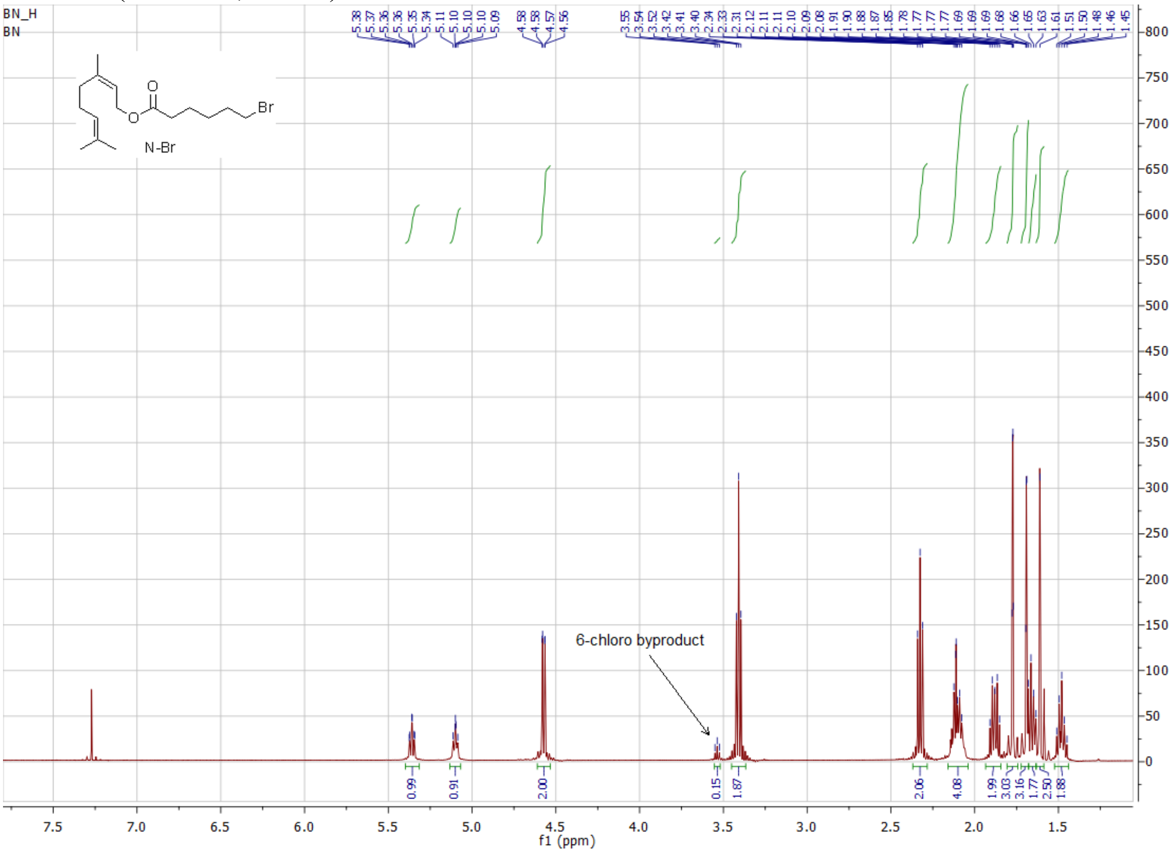

^{13}C NMR (126 MHz, $CDCl_3$):

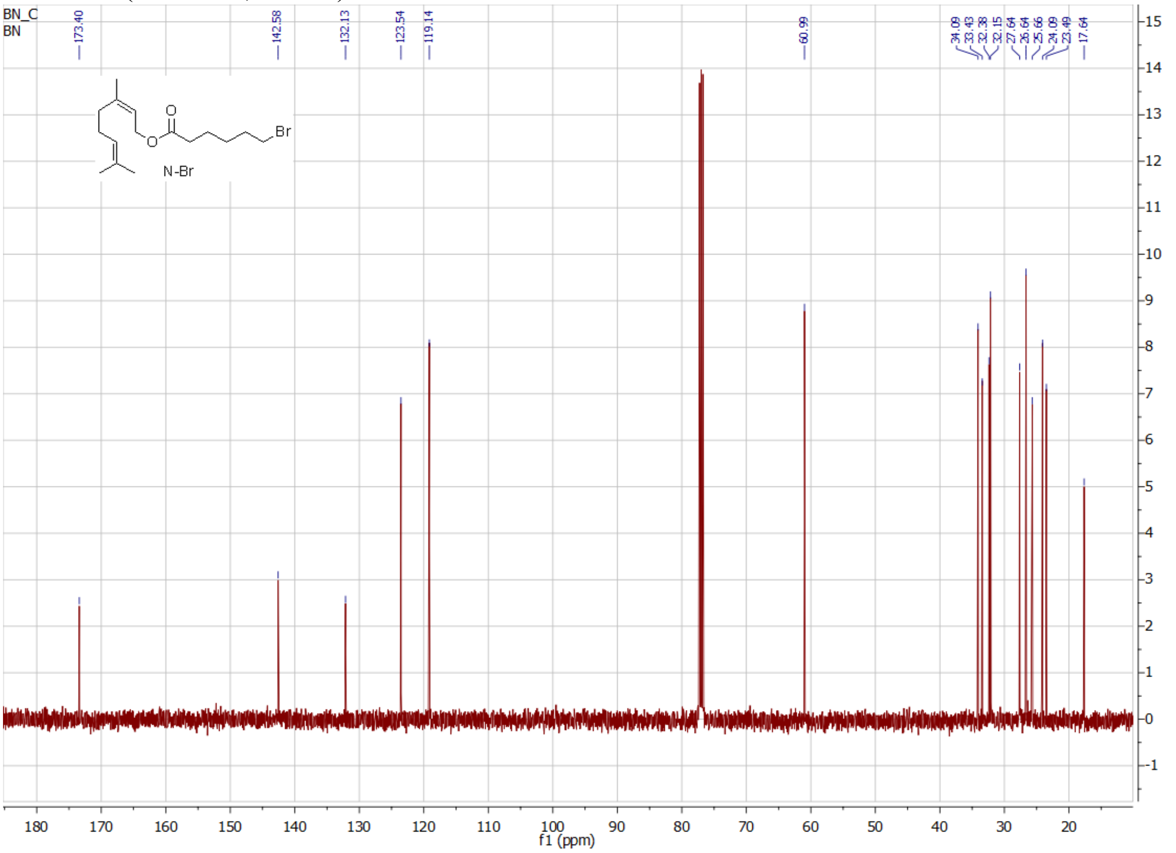

(*Z*)-3,7-dimethylocta-2,6-dien-1-yl 6-(dimethylamino)hexanoate (**N-DAK**)

^{1}H NMR (500 MHz, $CDCl_3$):

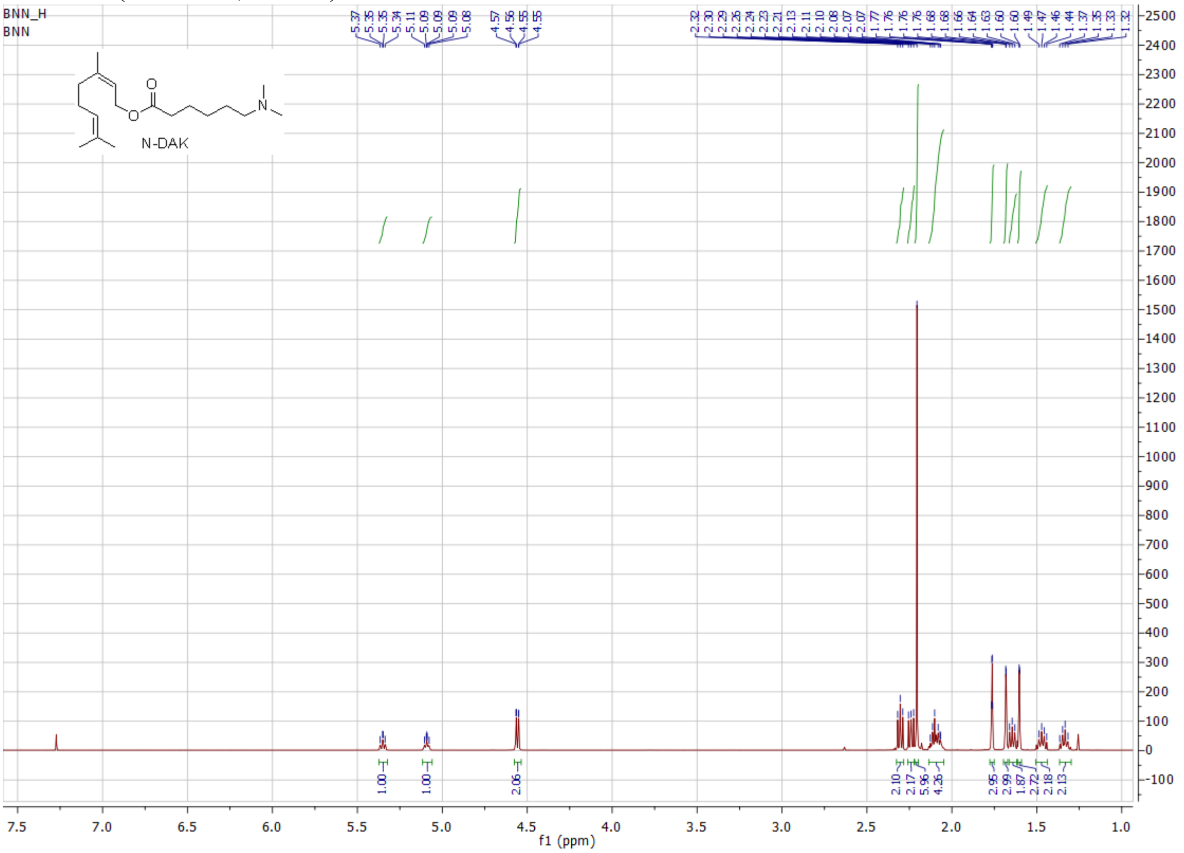

^{13}C NMR (126 MHz, $CDCl_3$):

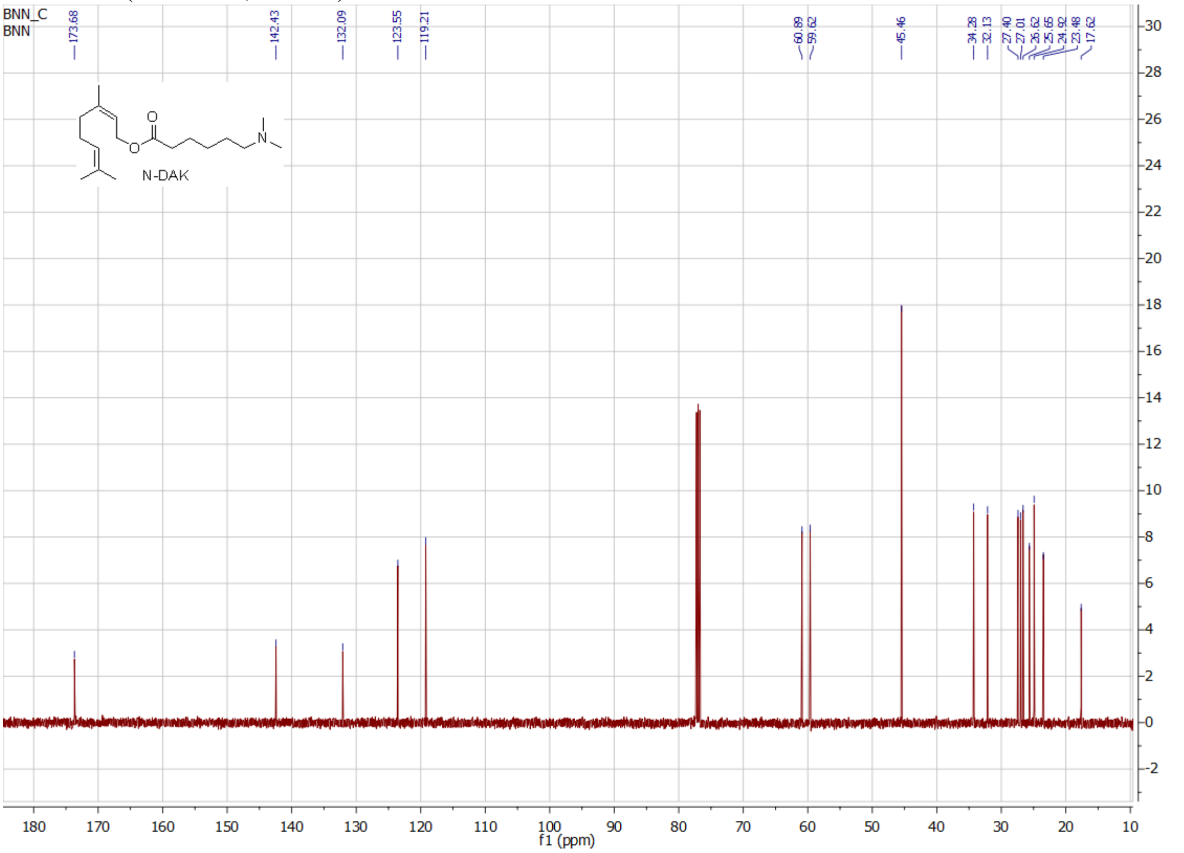

3,7,11-trimethyldodeca-2,6,10-trien-1-yl 6-bromohexanoate (**F-Br**)

^{1}H NMR (500 MHz, $CDCl_3$):

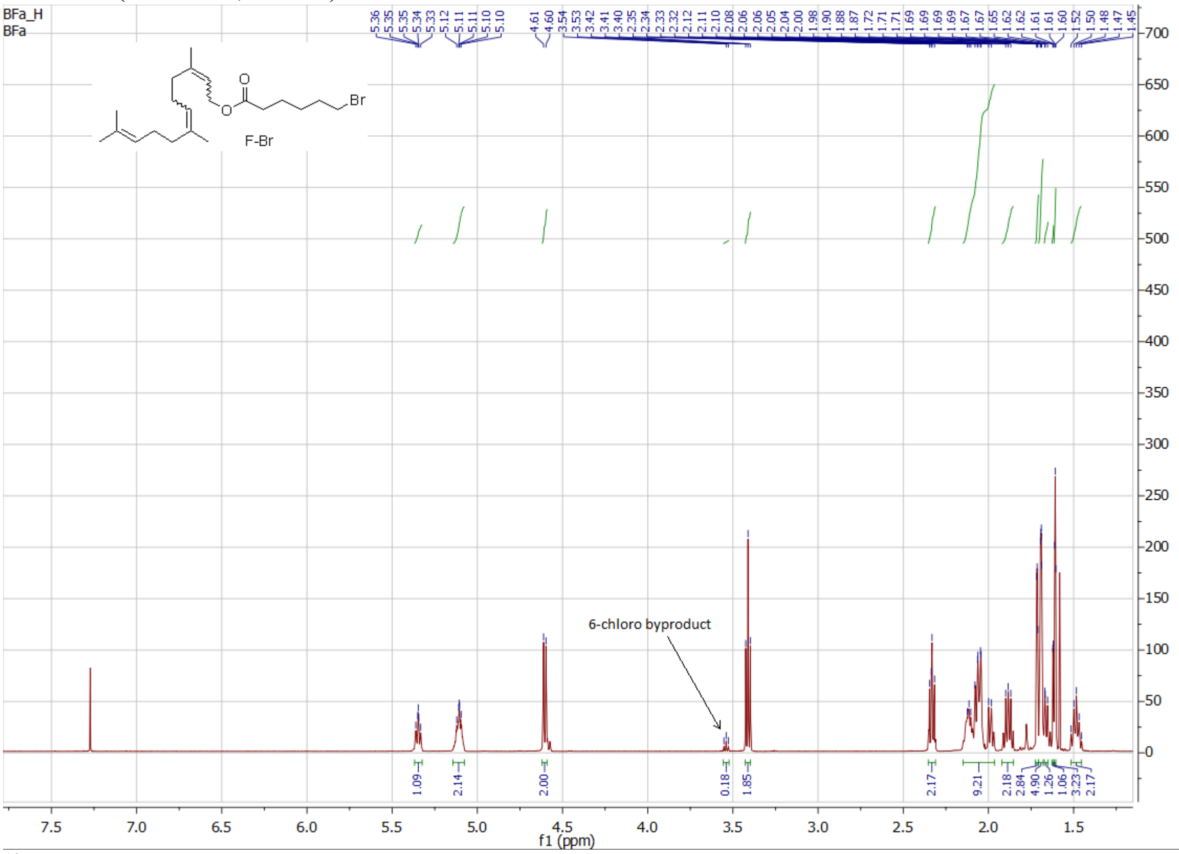

^{13}C NMR (126 MHz, $CDCl_3$):

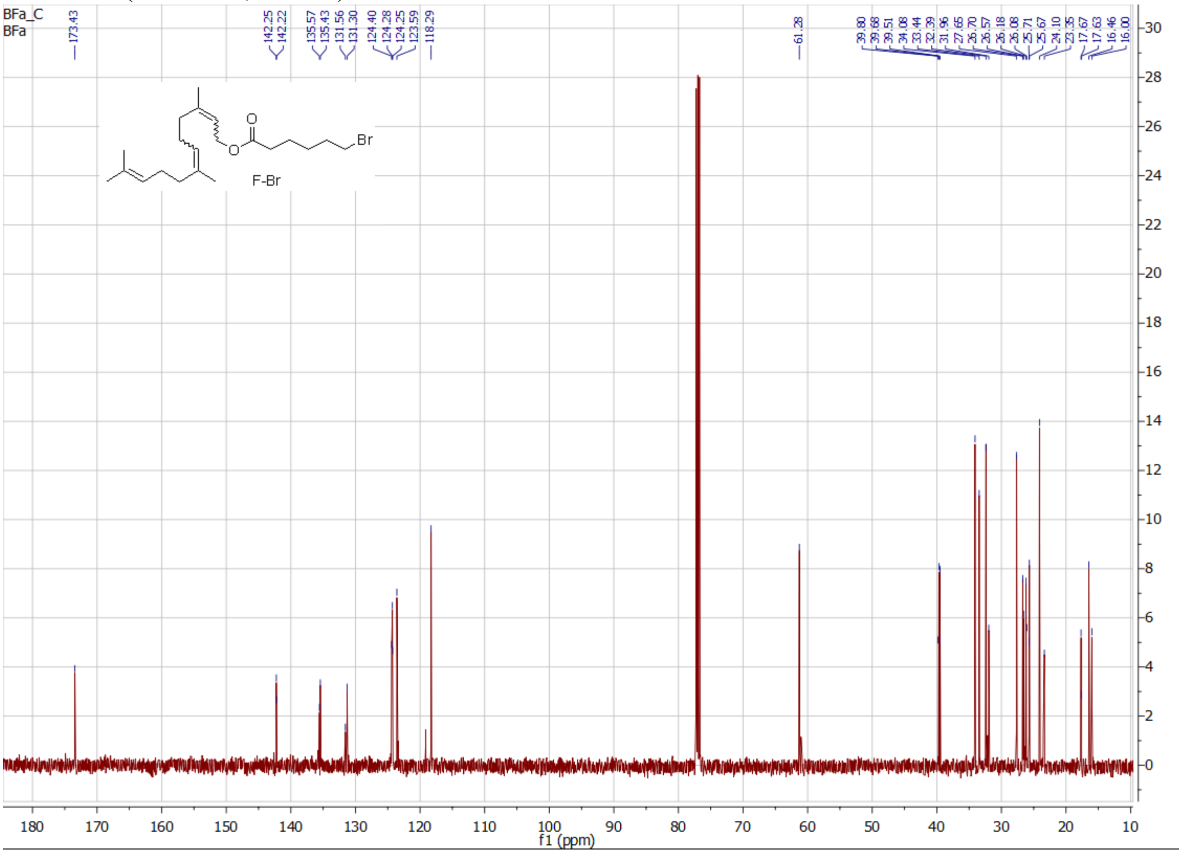

3,7,11-trimethyldodeca-2,6,10-trien-1-yl 6-(dimethylamino)hexanoate (**F-DAK**)

^{1}H NMR (500 MHz, $CDCl_3$):

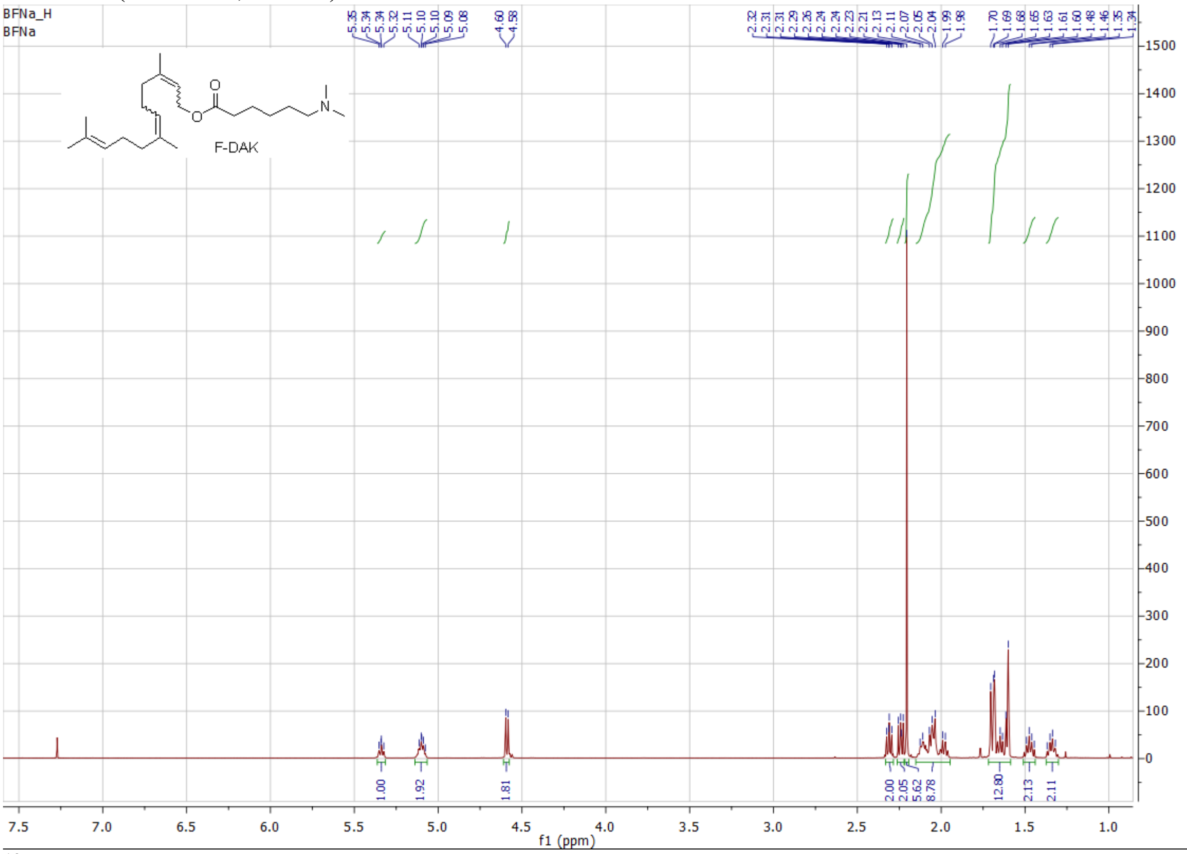

^{13}C NMR (126 MHz, $CDCl_3$):

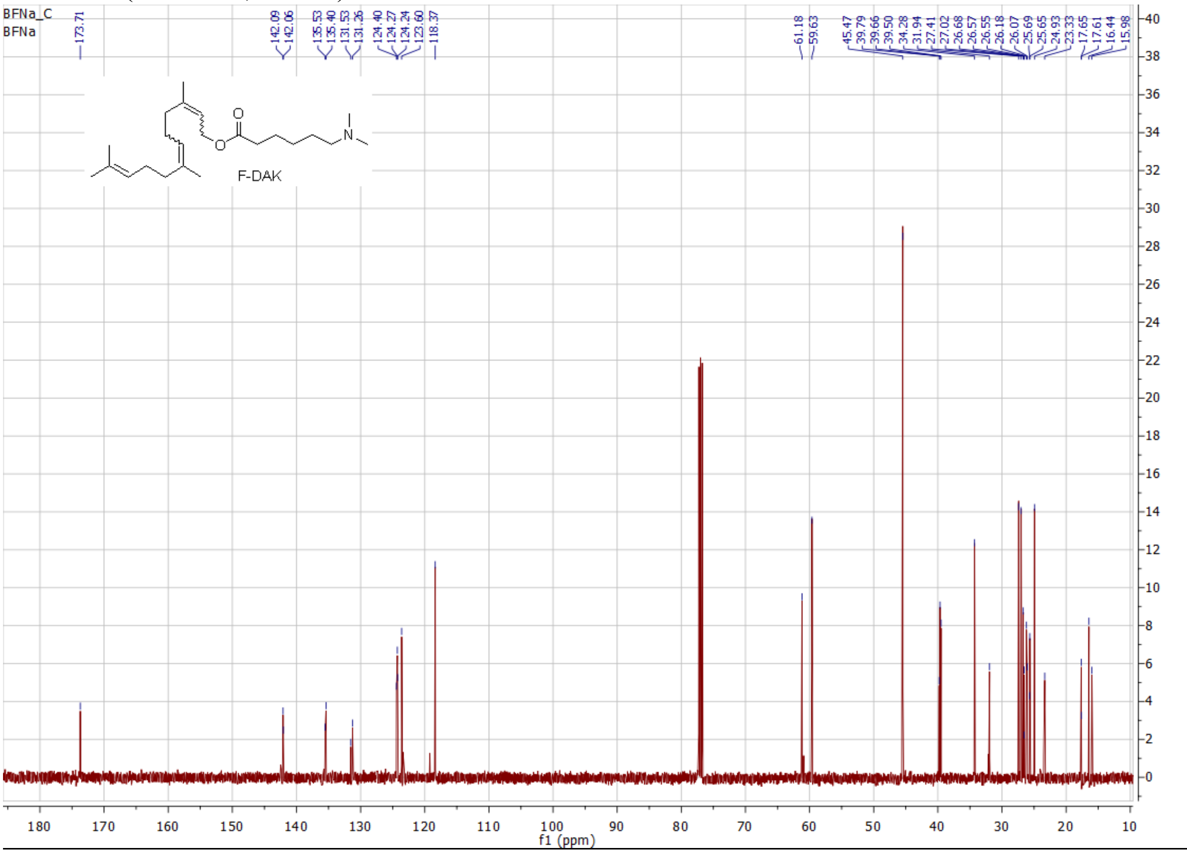

3,7-dimethylocta-1,6-dien-3-yl 6-bromohexanoate (**L-Br**)

^{1}H NMR (500 MHz, CDCl$_3$):

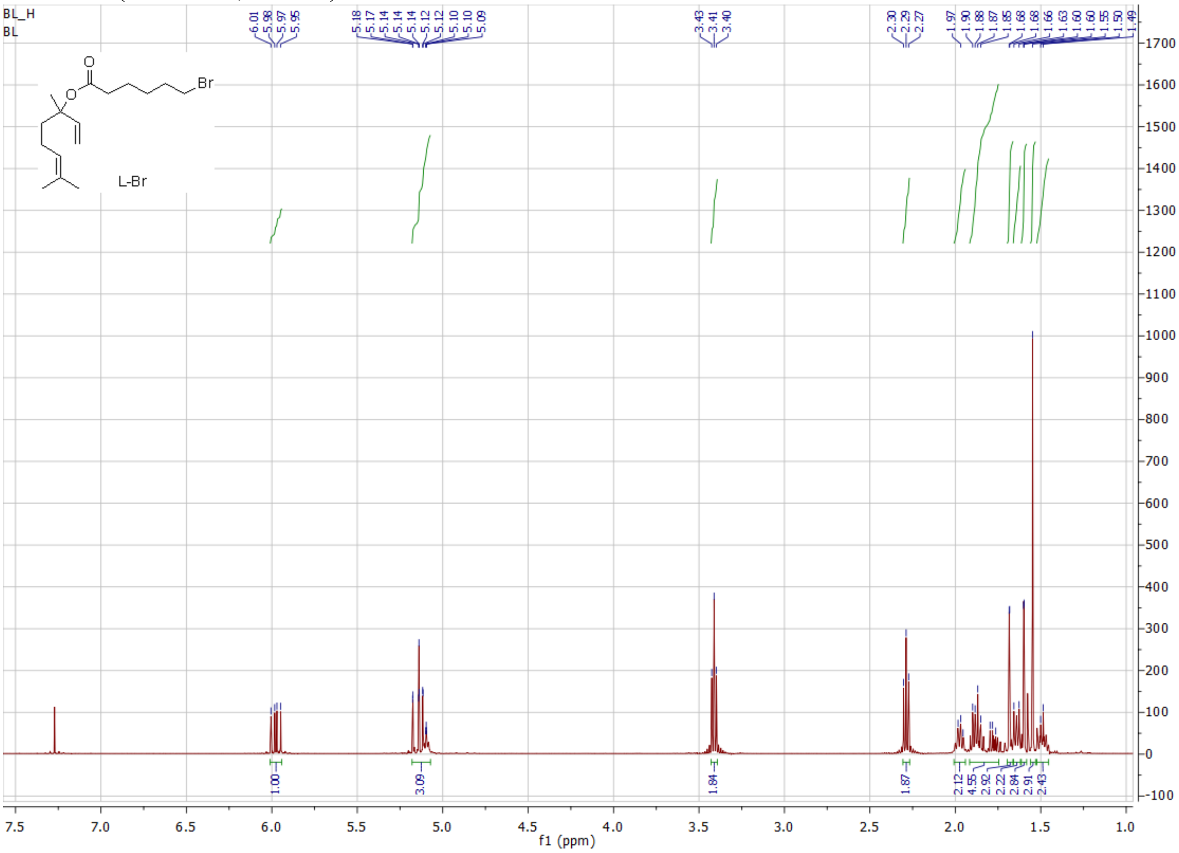

^{13}C NMR (126 MHz, CDCl$_3$):

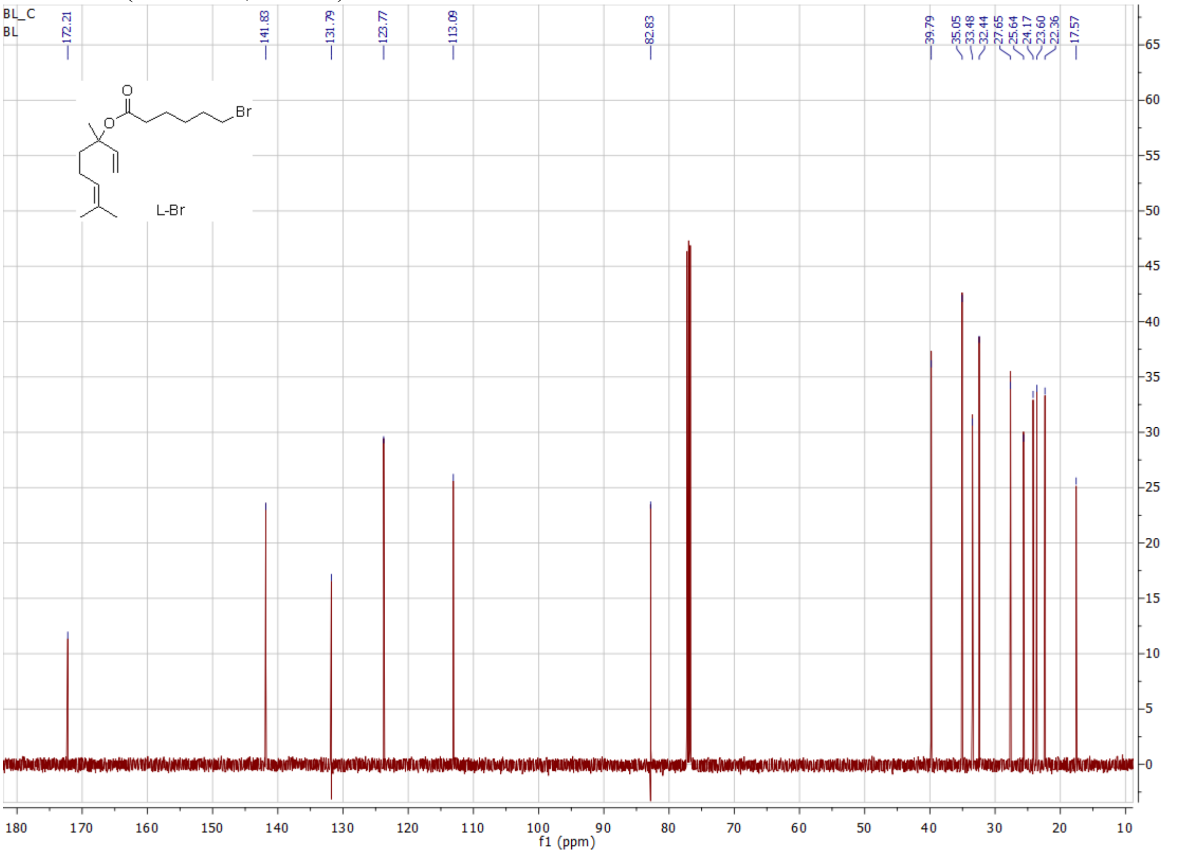

3,7-dimethylocta-1,6-dien-3-yl 6-(dimethylamino)hexanoate (**L-DAK**)

^{1}H NMR (500 MHz, $CDCl_3$):

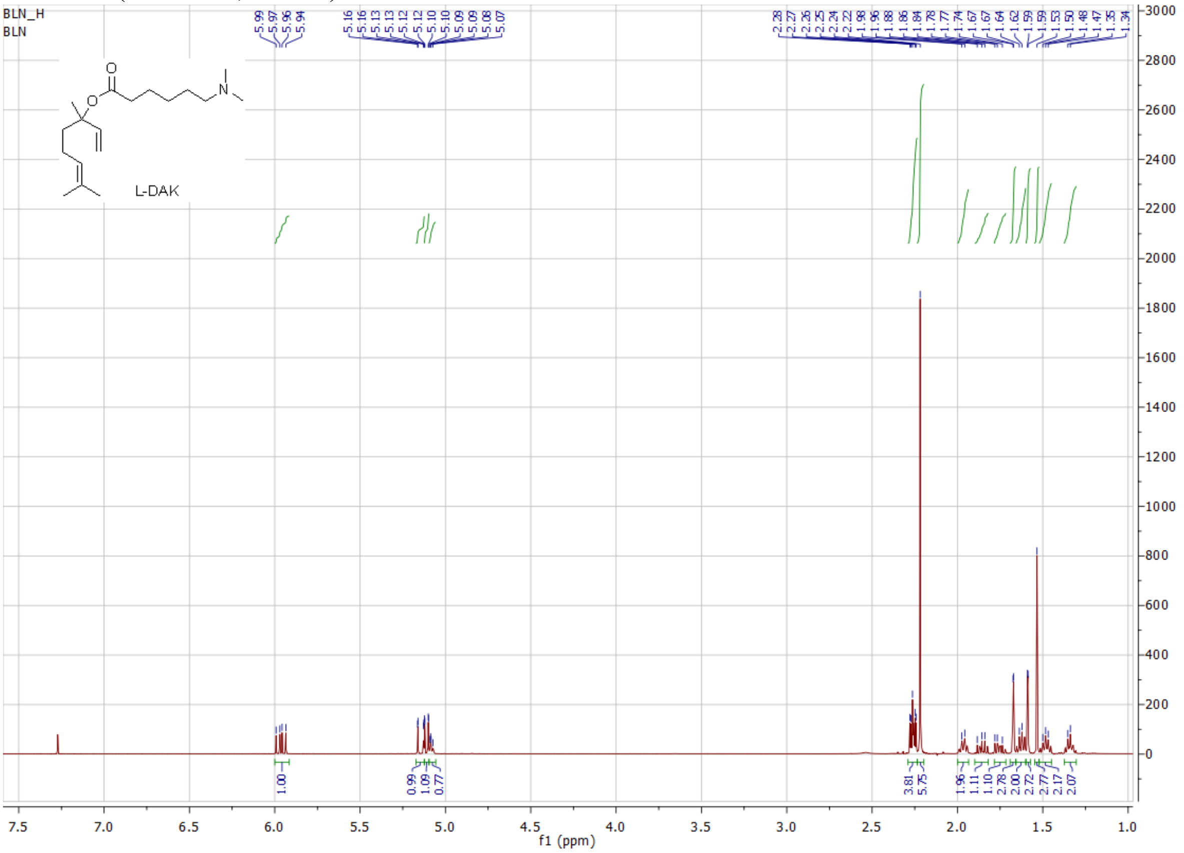

^{13}C NMR (126 MHz, $CDCl_3$):

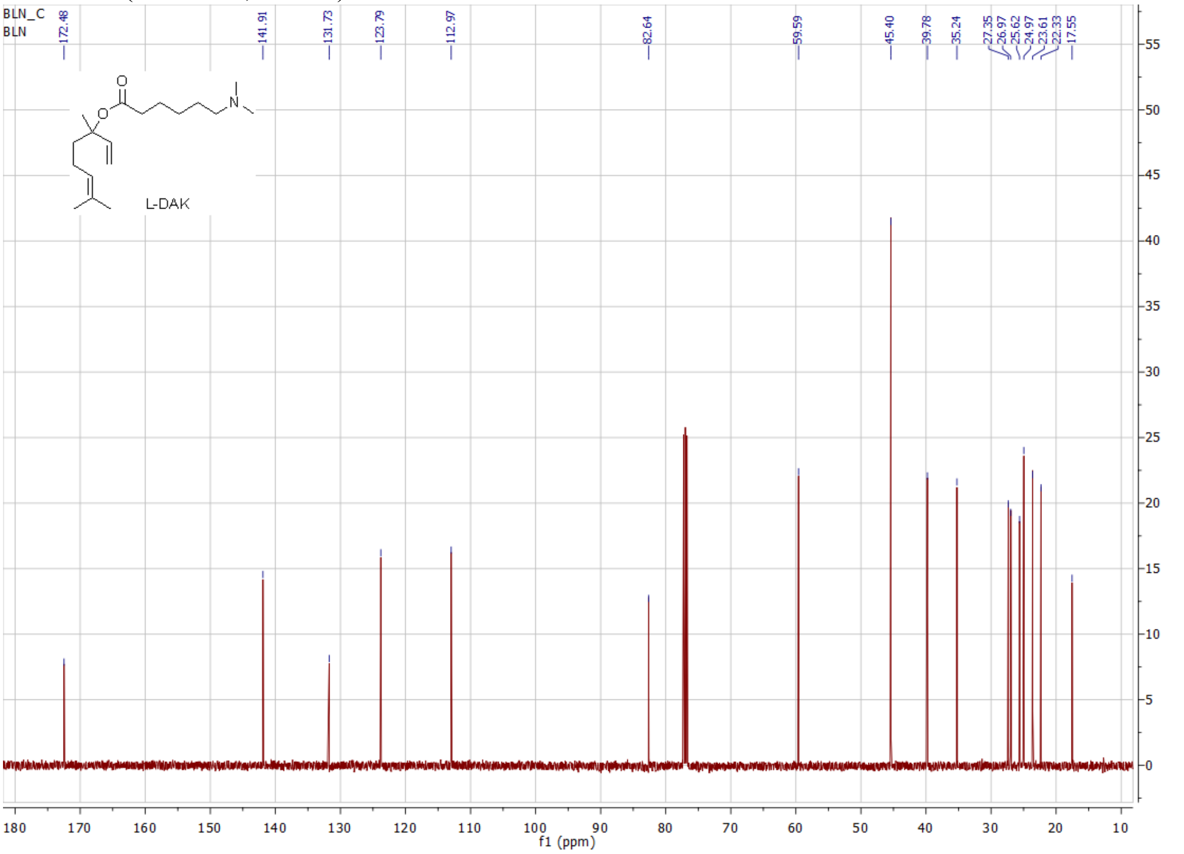

(*S*)-(4-(prop-1-en-2-yl)cyclohex-1-en-1-yl)methyl 6-bromohexanoate (**P-Br**)

^{1}H NMR (500 MHz, $CDCl_3$):

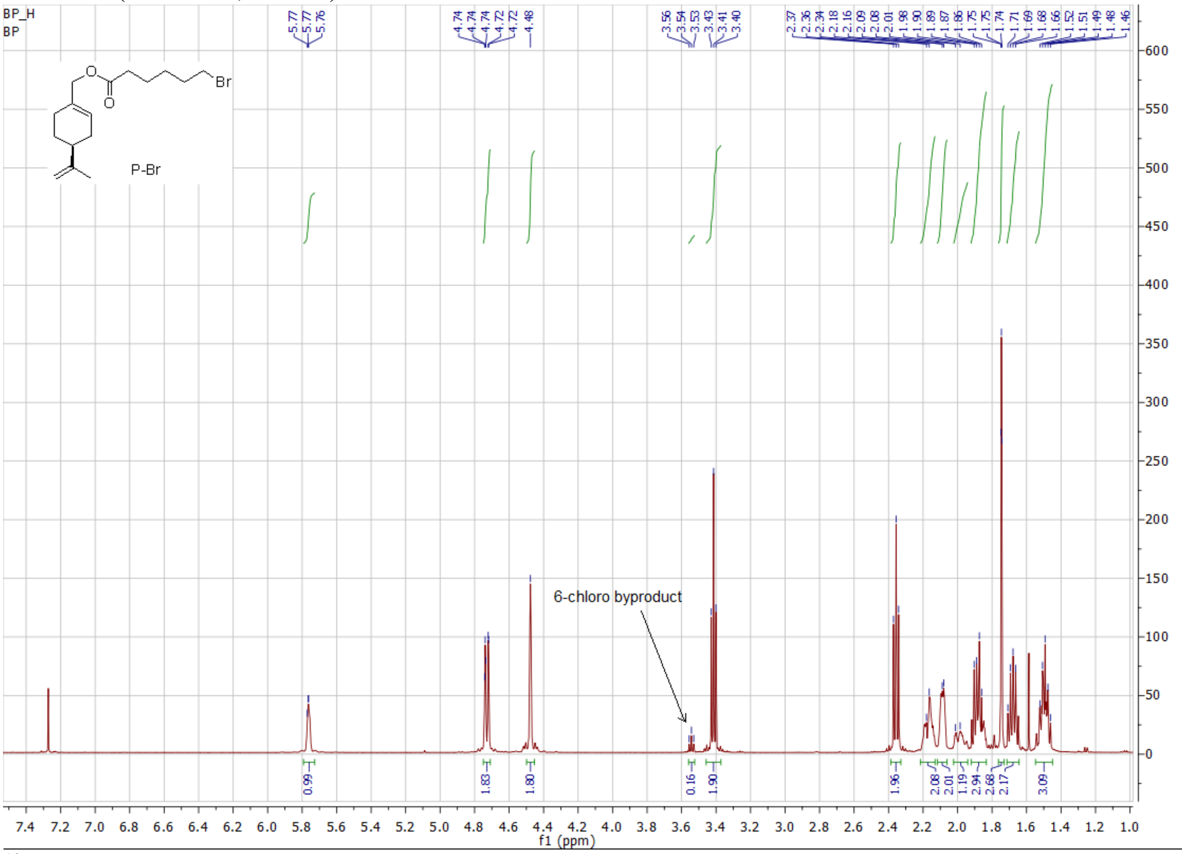

^{13}C NMR (126 MHz, $CDCl_3$):

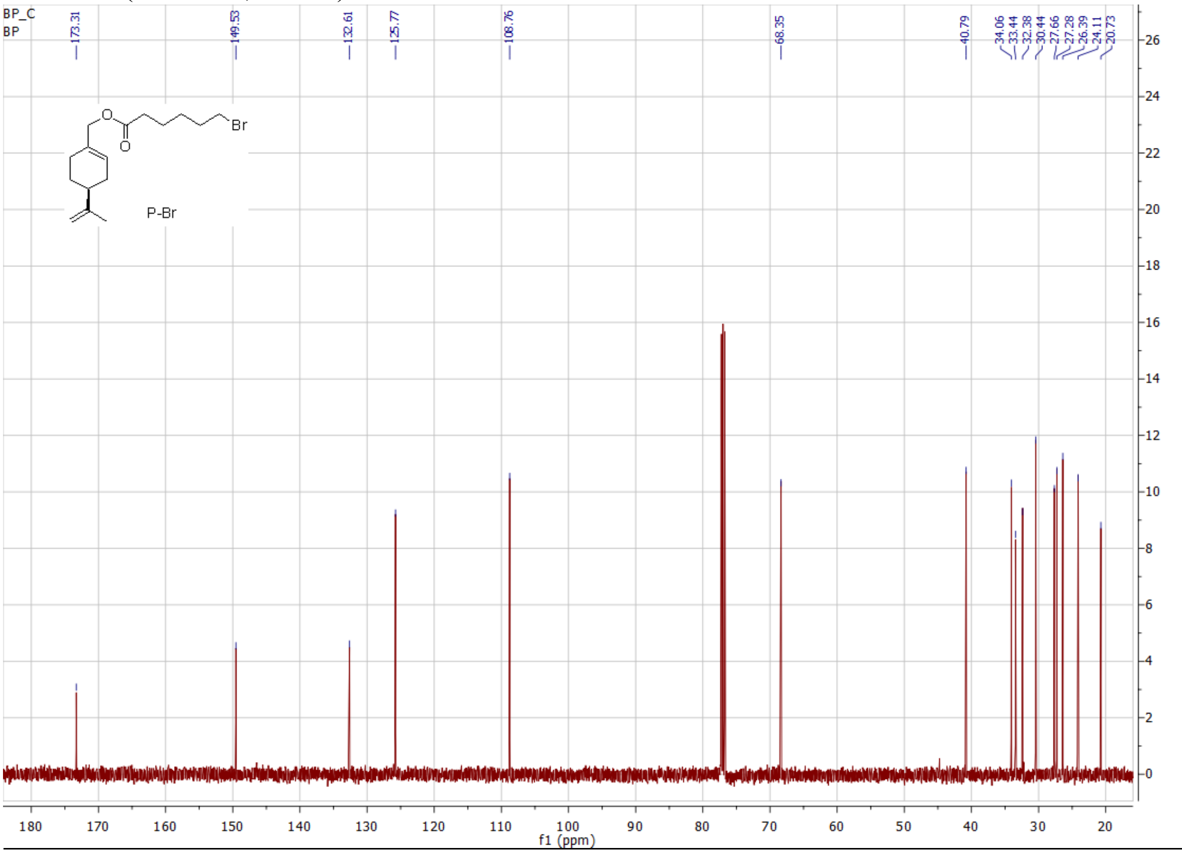

(*S*)-(4-(prop-1-en-2-yl)cyclohex-1-en-1-yl)methyl 6-(dimethylamino)hexanoate (**P-DAK**)

H NMR (500 MHz, $CDCl_3$):

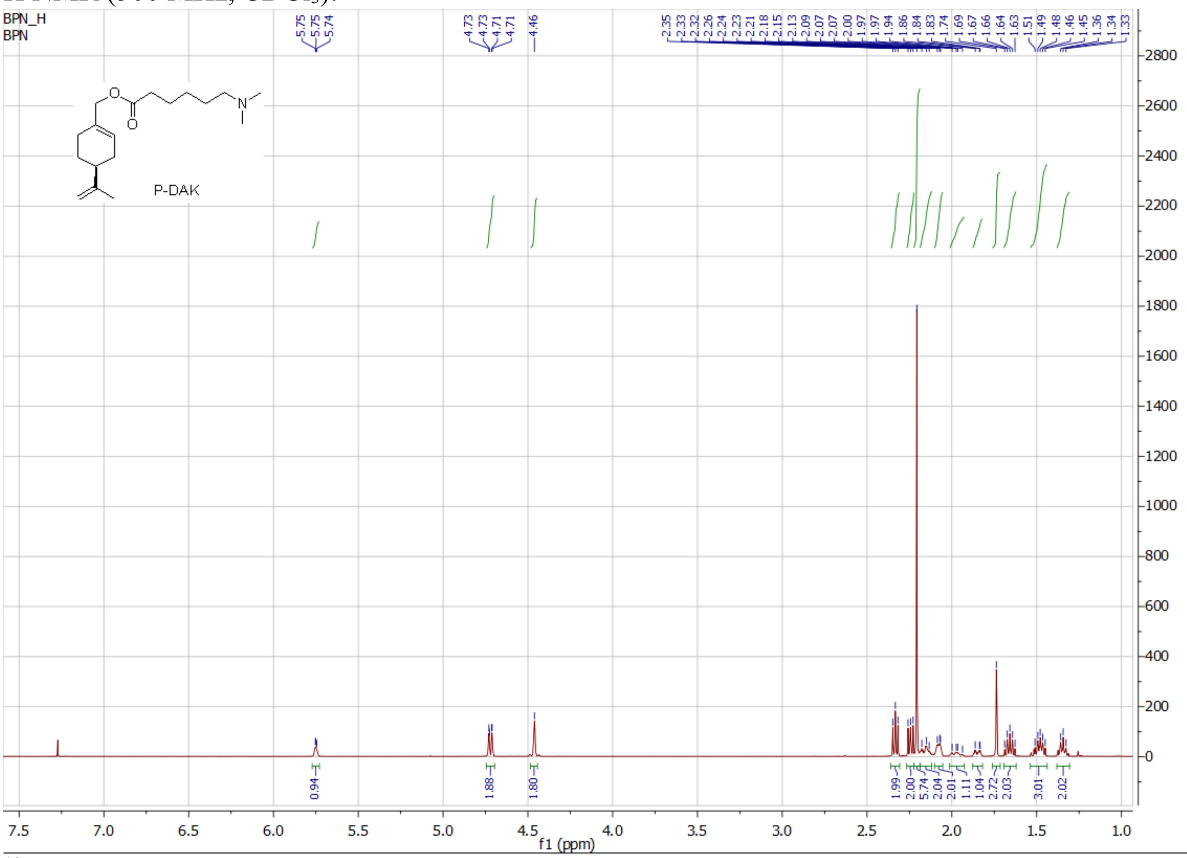

^{13}C NMR (126 MHz, $CDCl_3$):

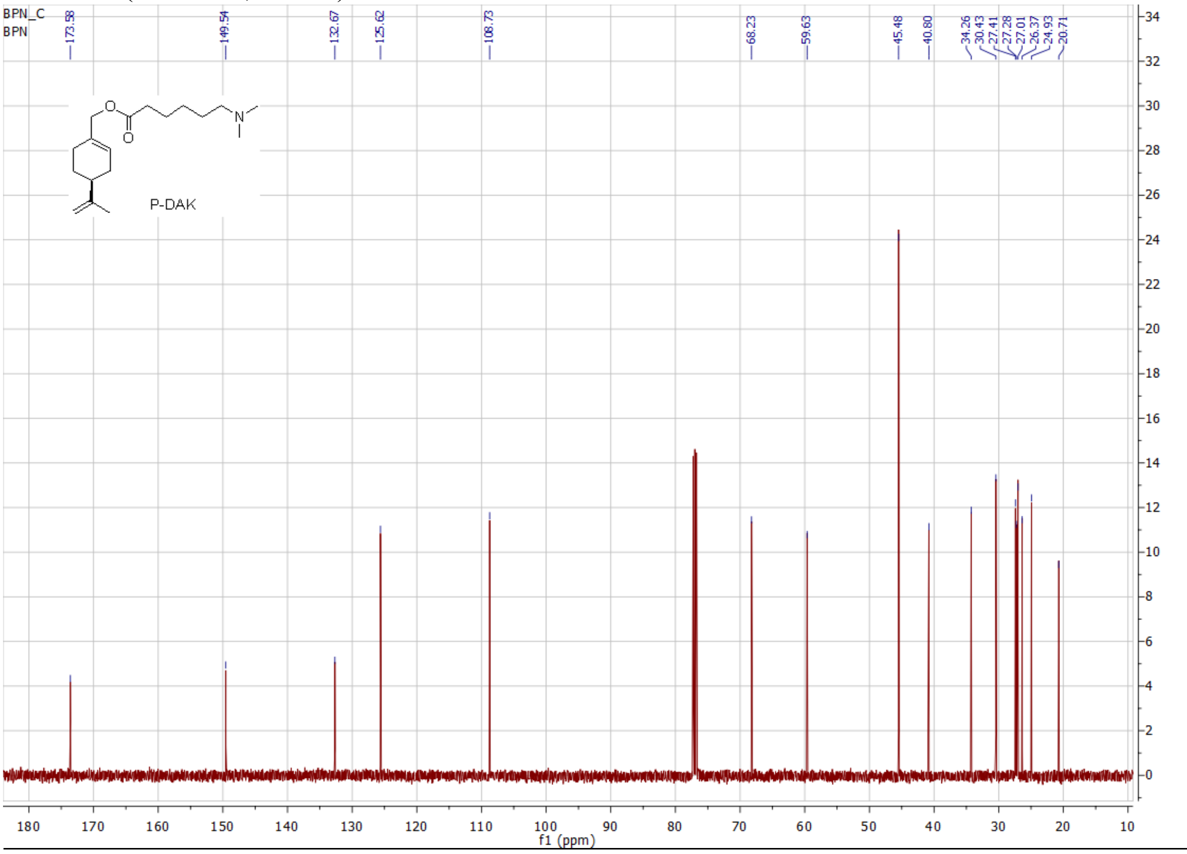

(1*R*,2*S*,5*R*)-2-isopropyl-5-methylcyclohexyl 6-bromohexanoate (**M-Br**)

^{1}H NMR (500 MHz, $CDCl_3$):

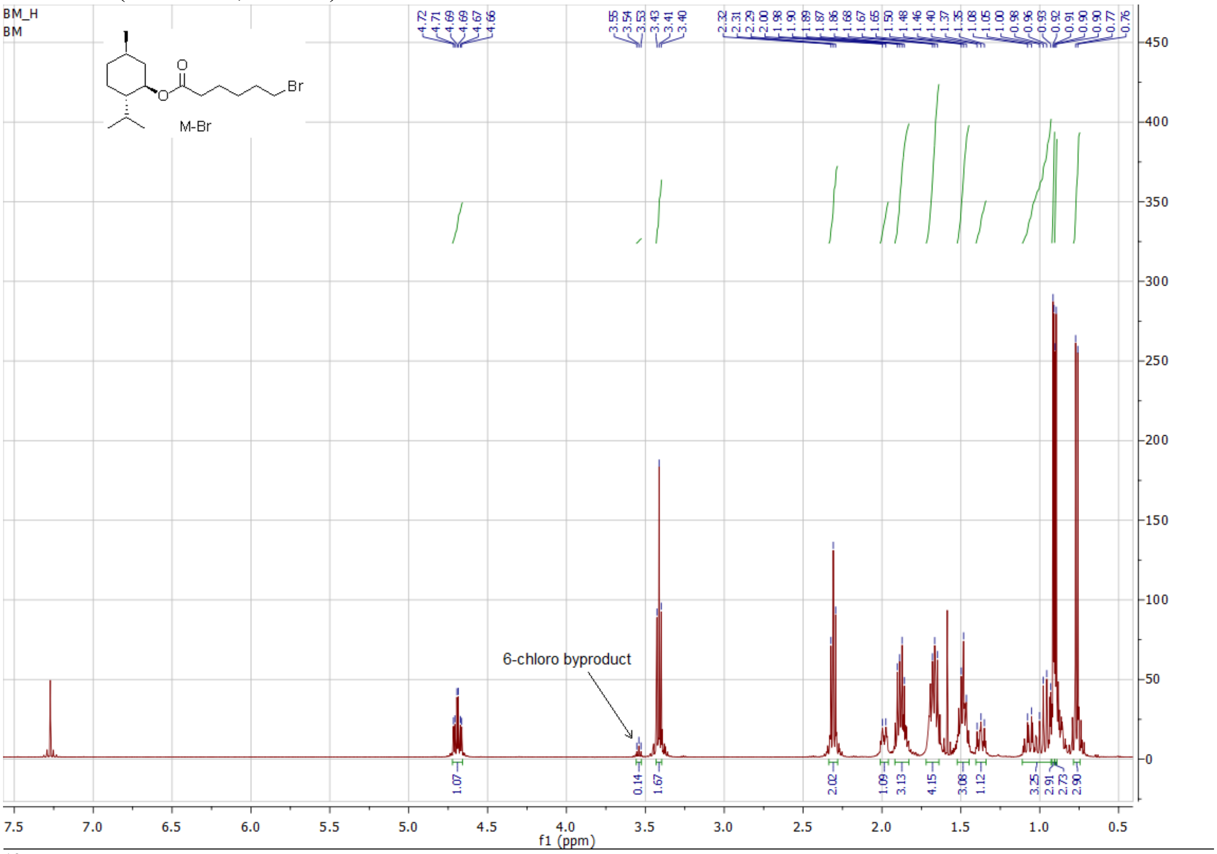

^{13}C NMR (126 MHz, $CDCl_3$):

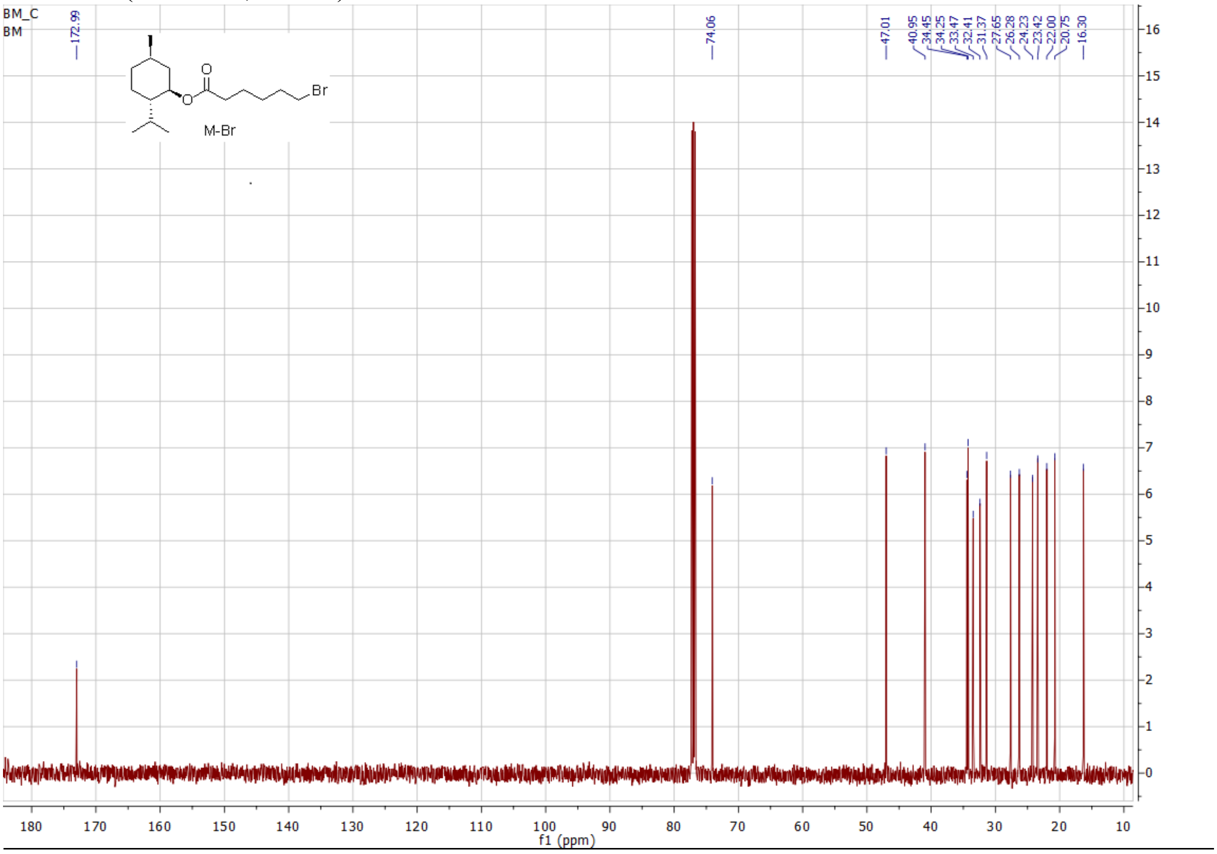

(1*R*,2*S*,5*R*)-2-isopropyl-5-methylcyclohexyl 6-(dimethylamino)hexanoate (**M-DAK**)

^{1}H NMR (300 MHz, $CDCl_3$):

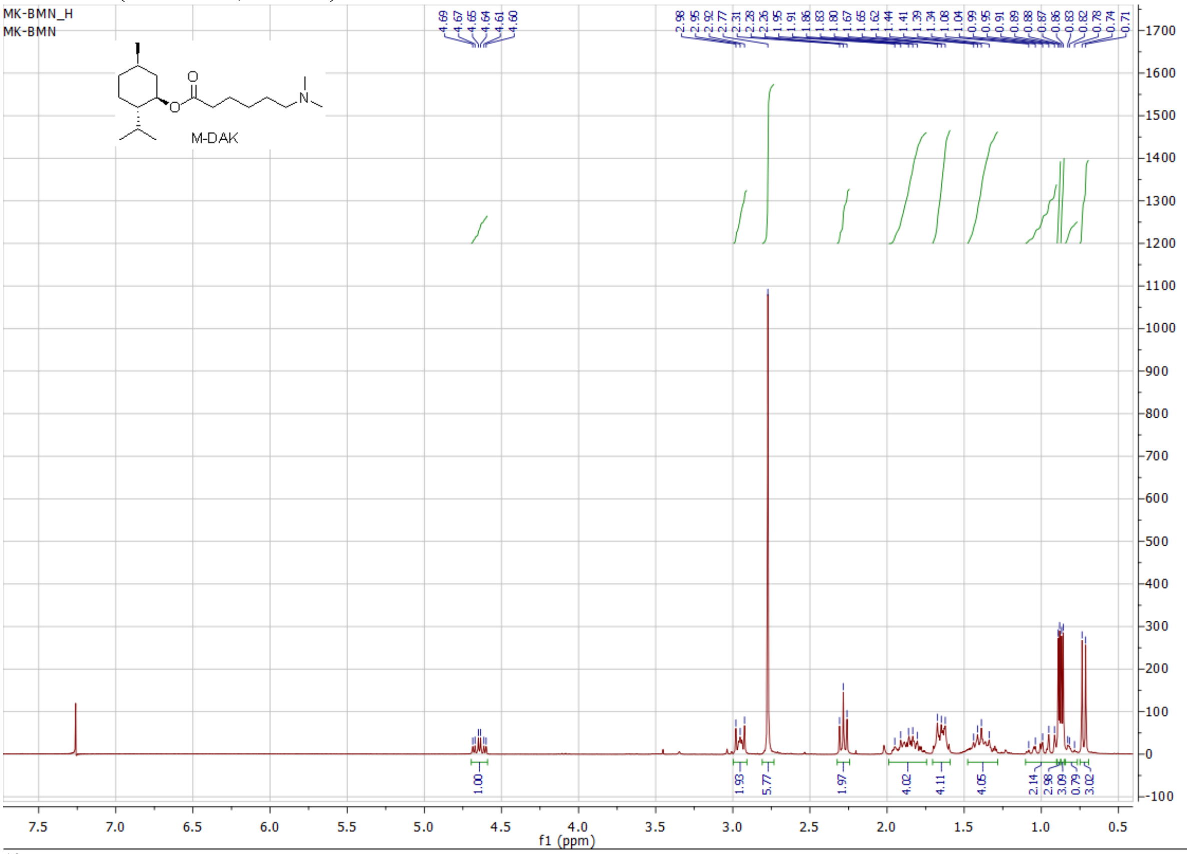

^{13}C NMR (75 MHz, $CDCl_3$):

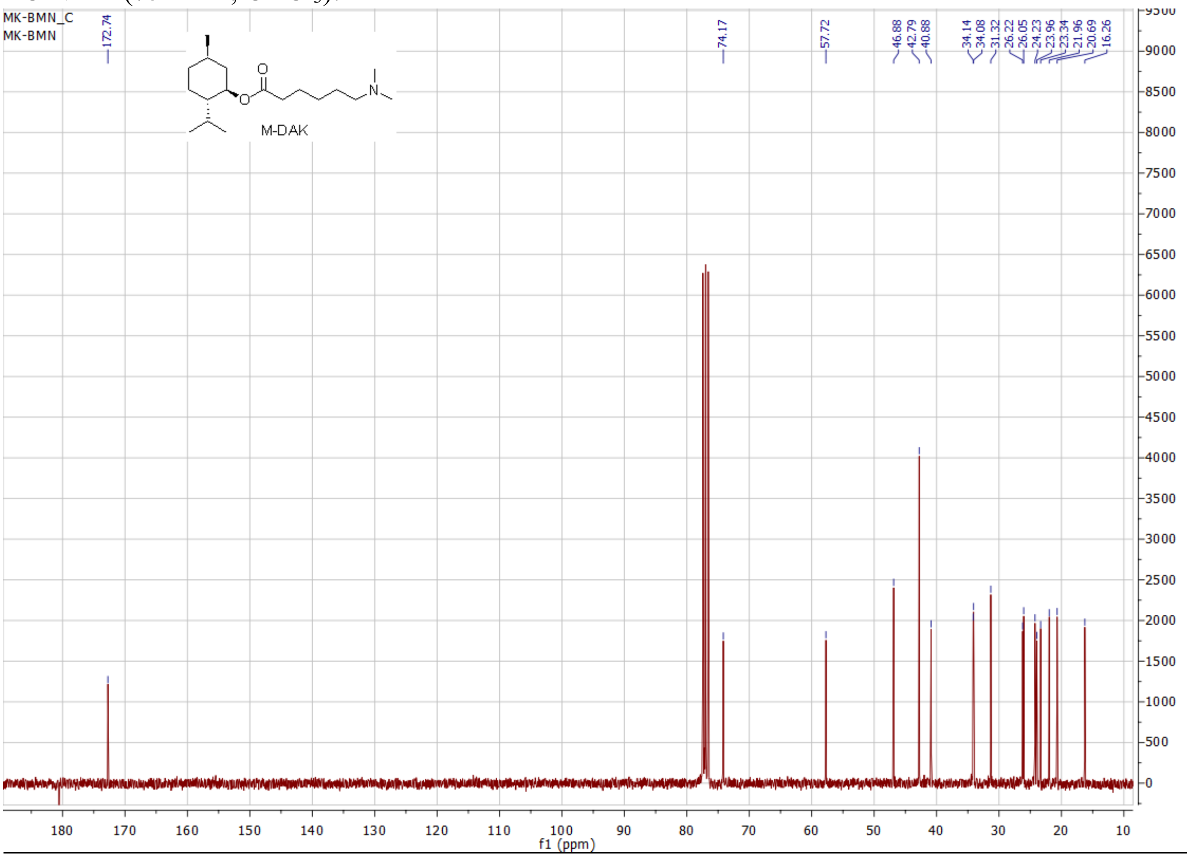

(1*S*,4*S*)-1,7,7-trimethylbicyclo[2.2.1]heptan-2-yl 6-bromohexanoate (**B-Br**)

^{1}H NMR (500 MHz, $CDCl_3$):

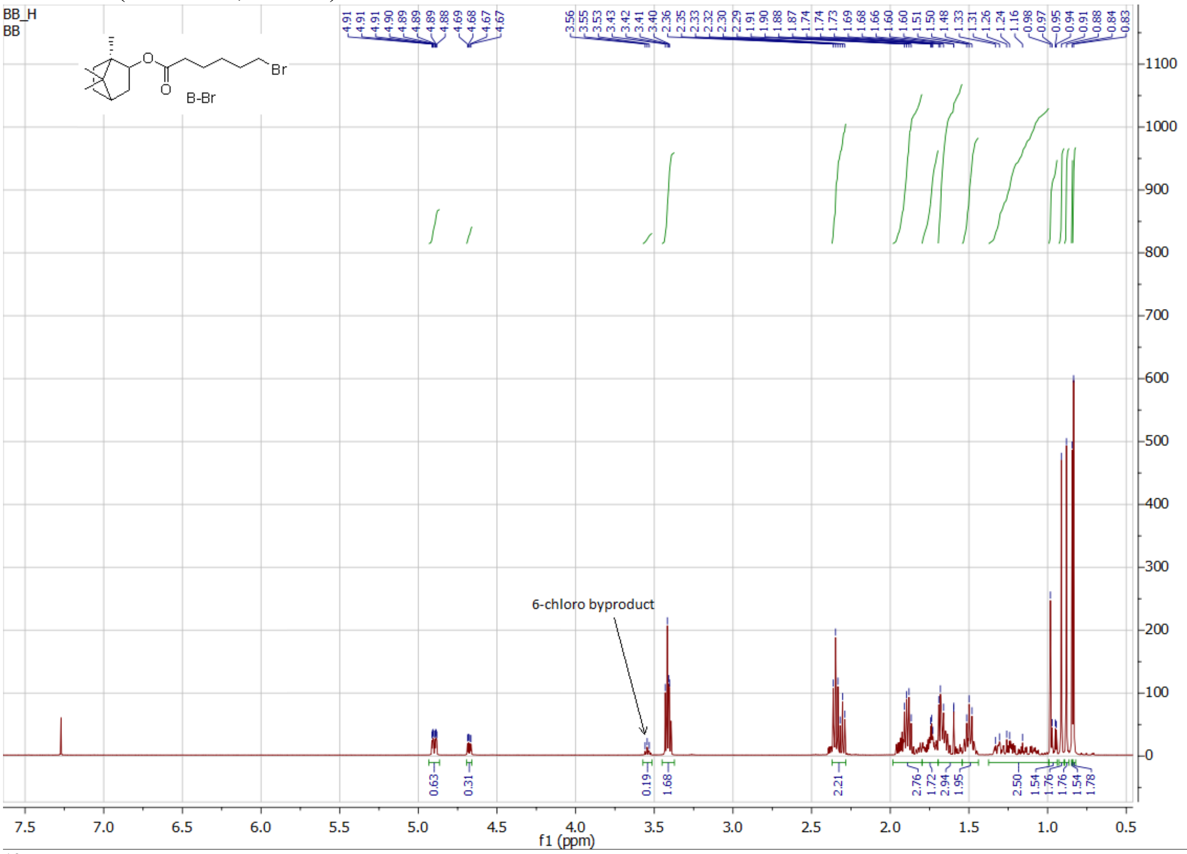

^{13}C NMR (126 MHz, $CDCl_3$):

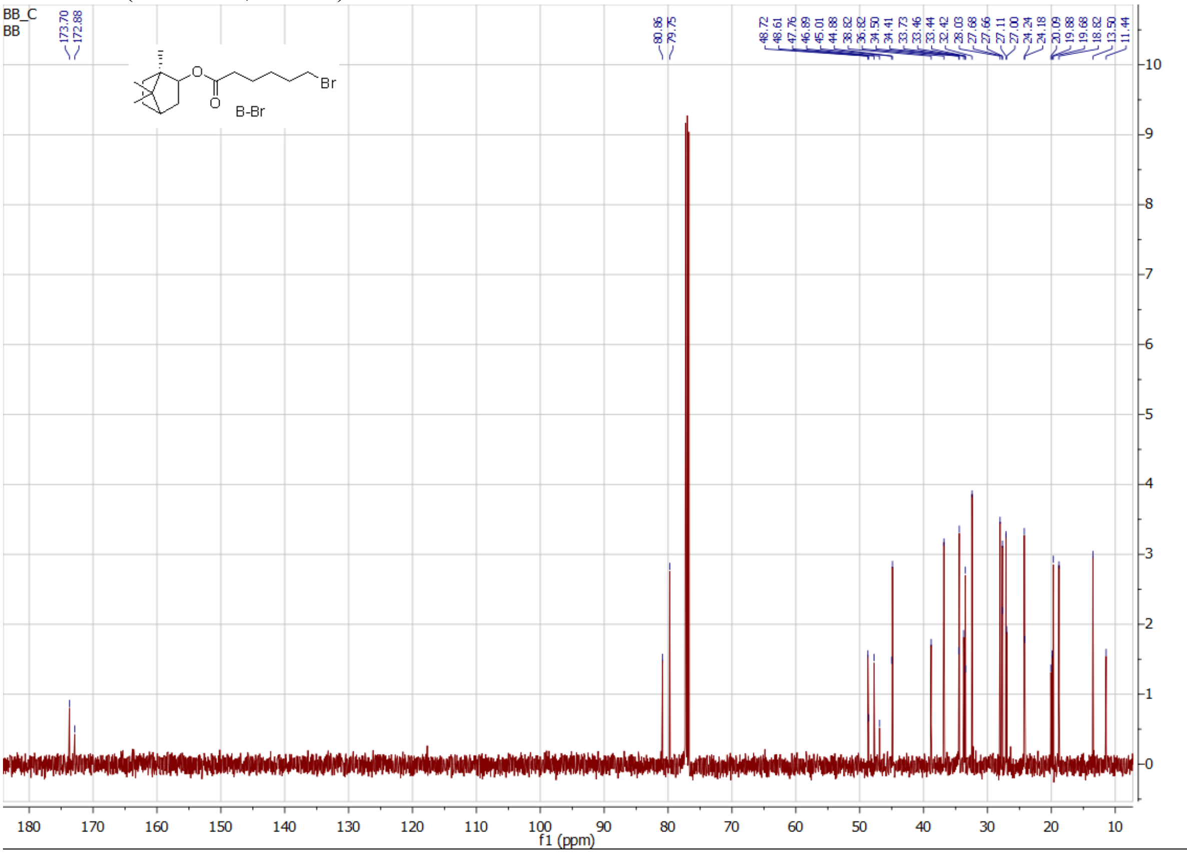

(1*S*,4*S*)-1,7,7-trimethylbicyclo[2.2.1]heptan-2-yl 6-(dimethylamino)hexanoate (**B-DAK**)

^{1}H NMR (500 MHz, $CDCl_3$):

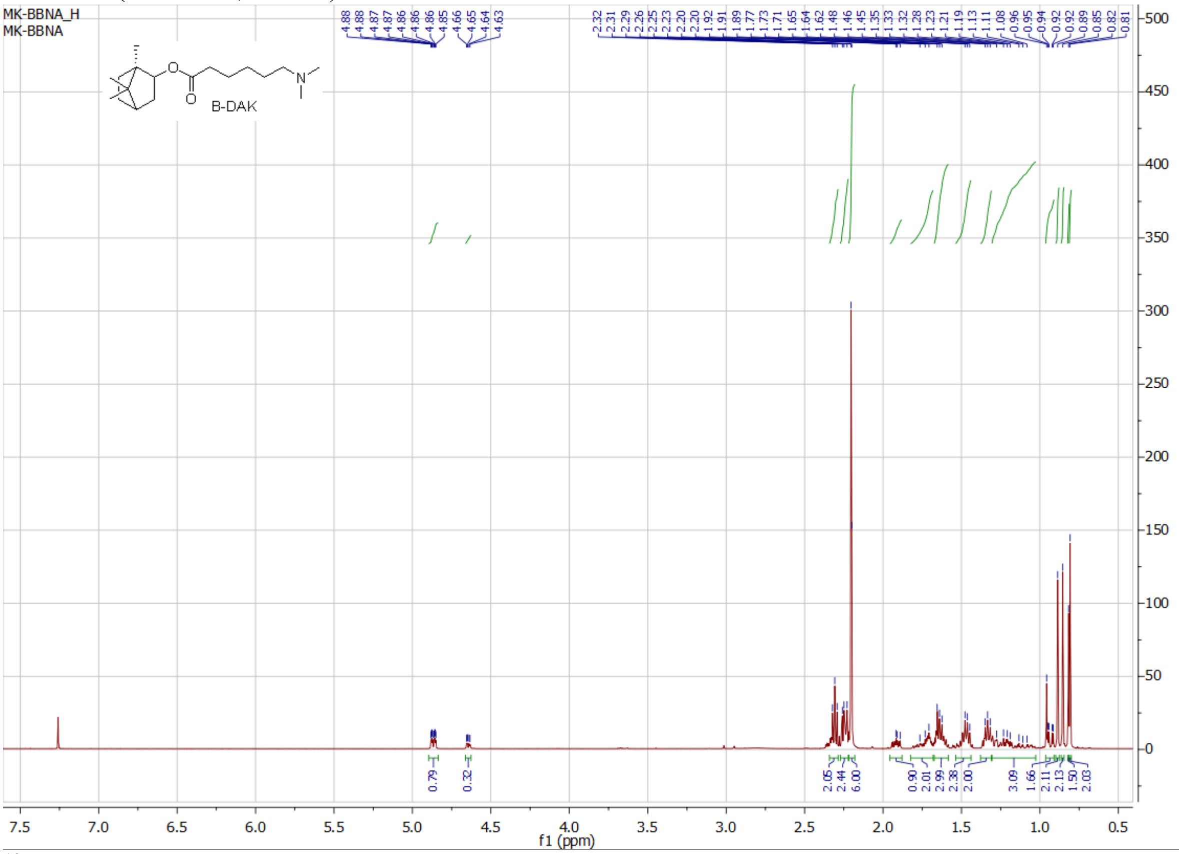

^{13}C NMR (126 MHz, $CDCl_3$):

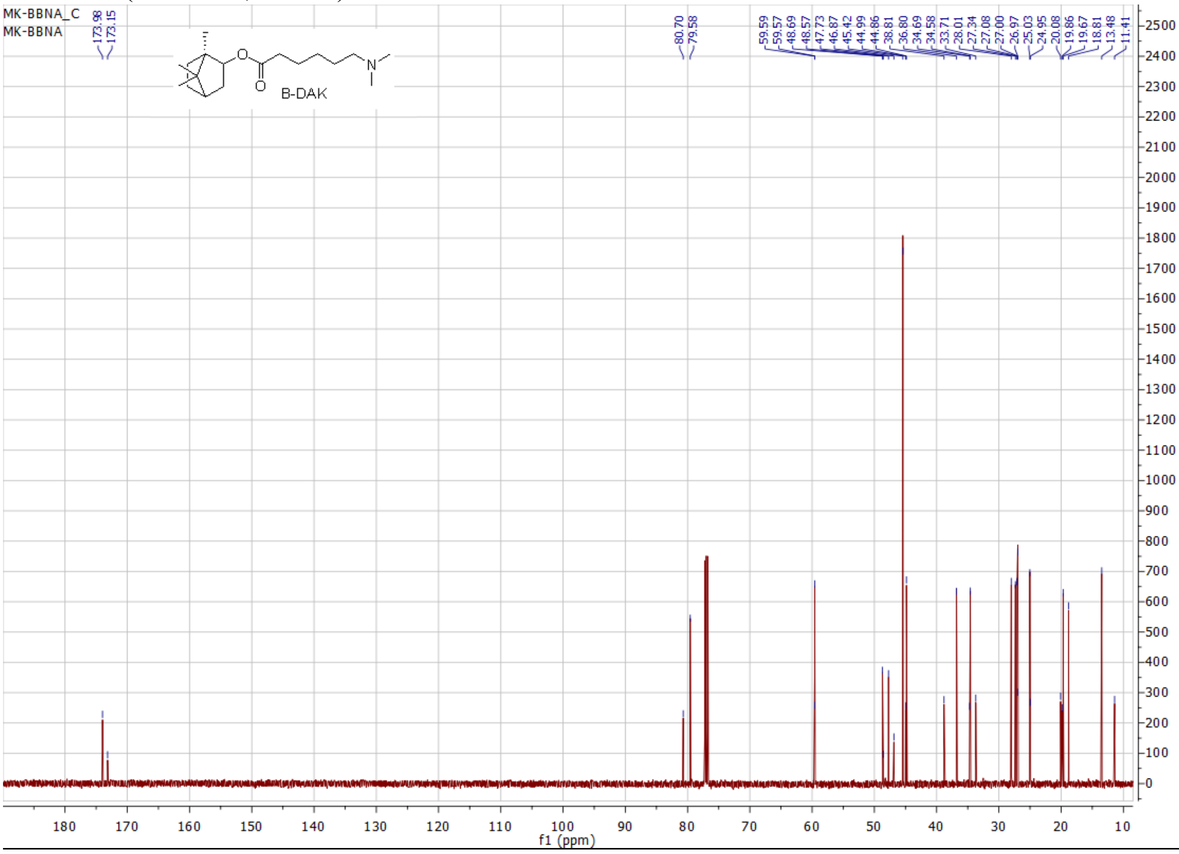

(5*R*)-2-methyl-5-(prop-1-en-2-yl)cyclohex-2-en-1-yl 6-bromohexanoate (**Ca-Br**)

^{1}H NMR (500 MHz, $CDCl_3$):

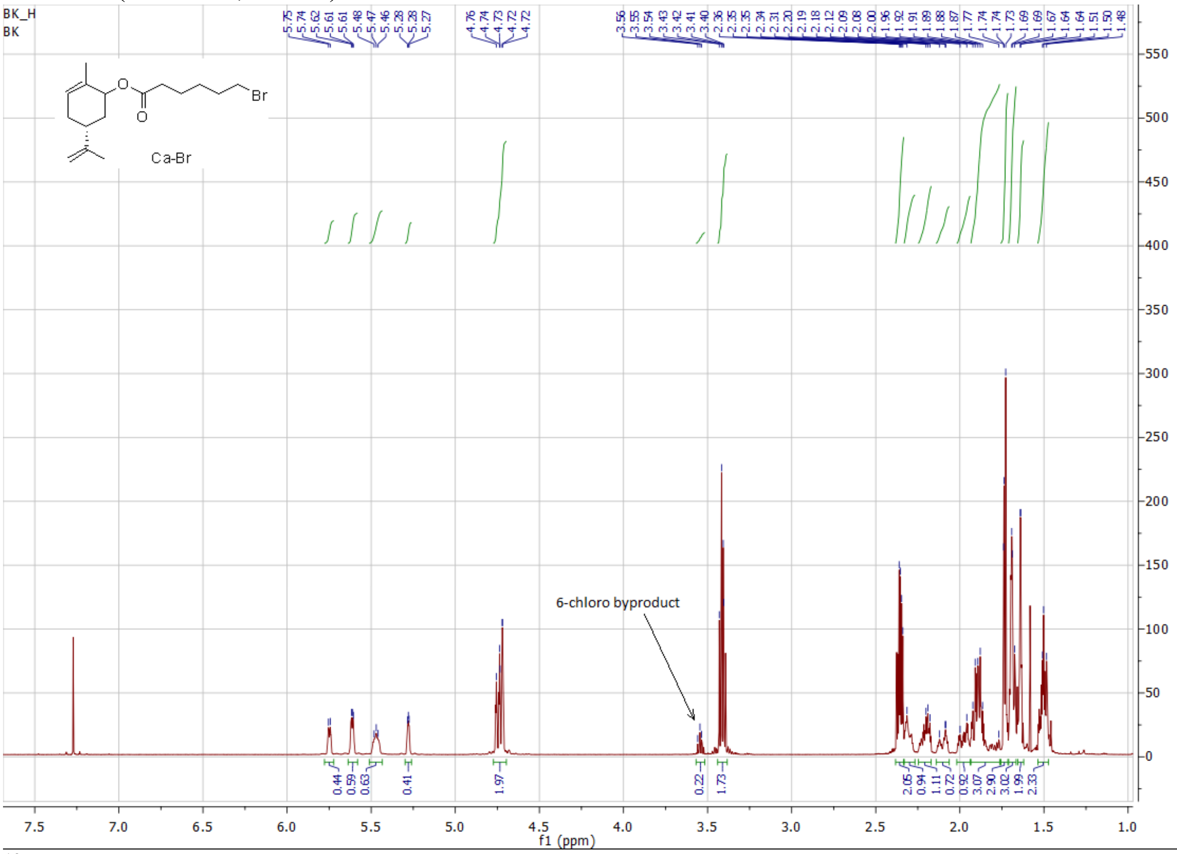

^{13}C NMR (126 MHz, $CDCl_3$):

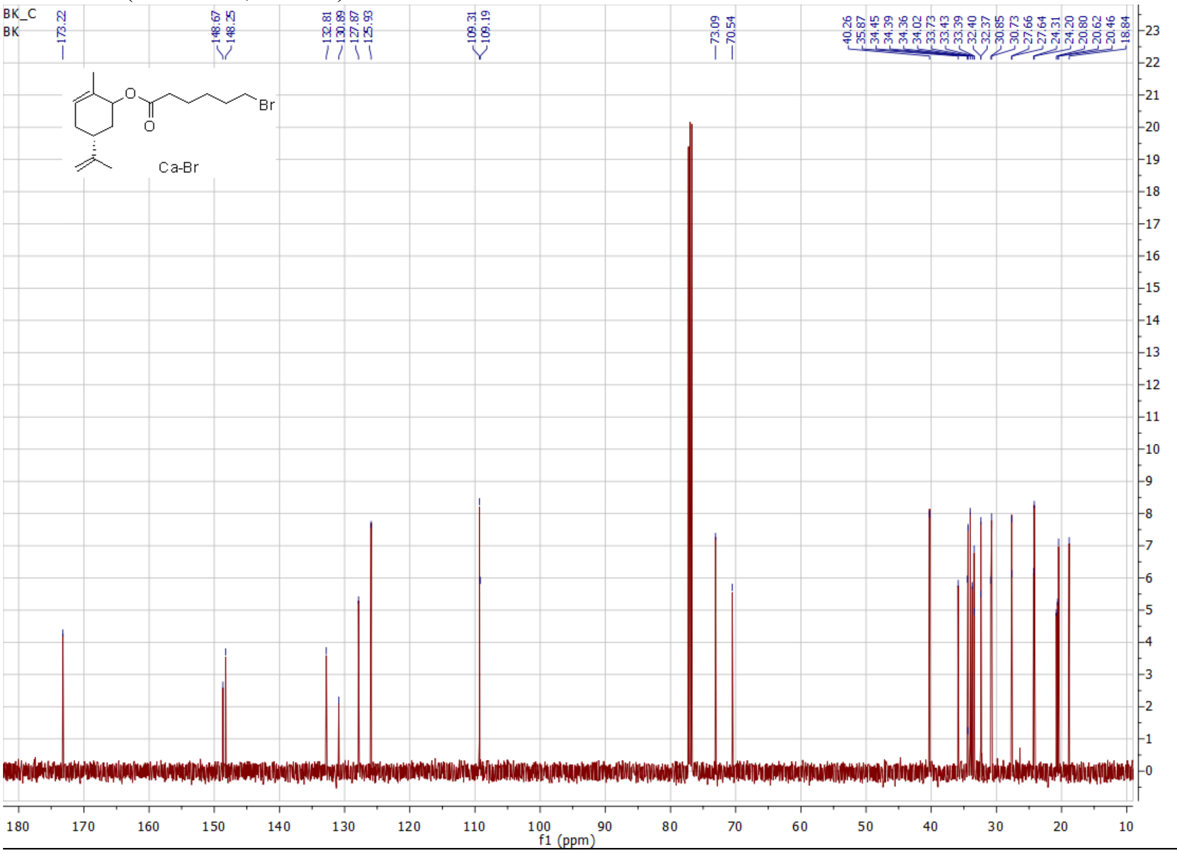

(5*R*)-2-methyl-5-(prop-1-en-2-yl)cyclohex-2-en-1-yl 6-(dimethylamino)hexanoate (**Ca-DAK**)

^{1}H NMR (500 MHz, $CDCl_3$):

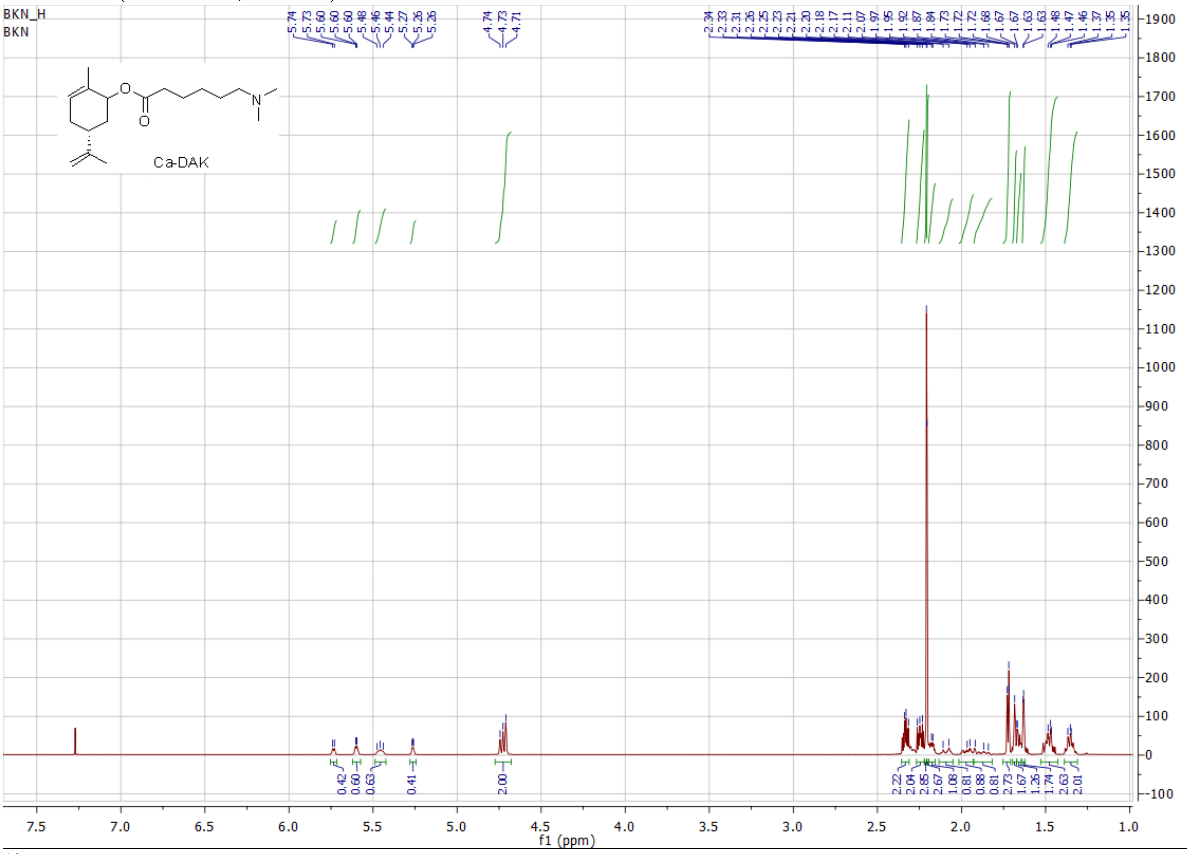

^{13}C NMR (126 MHz, $CDCl_3$):

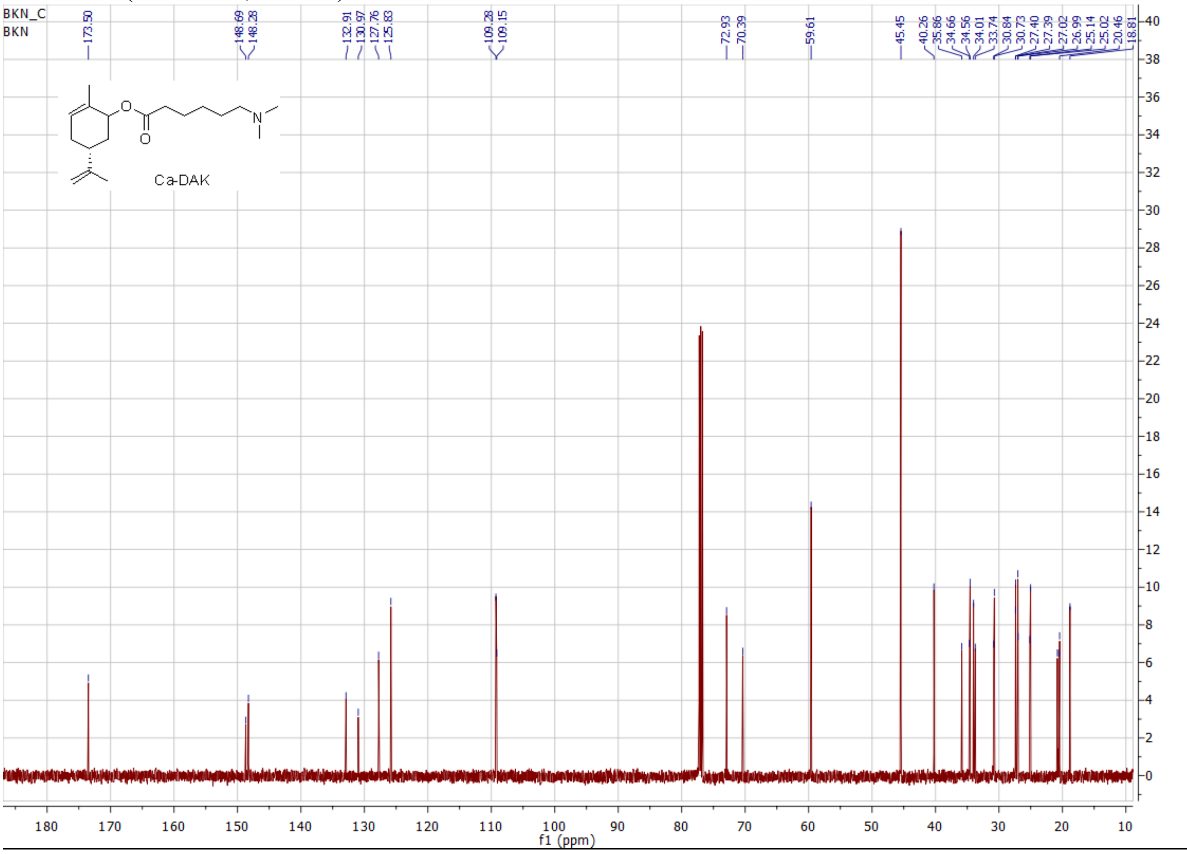

(*E*)-3-phenylprop-2-en-1-yl 6-bromohexanoate (**Ci-Br**)

^{1}H NMR (500 MHz, $CDCl_3$):

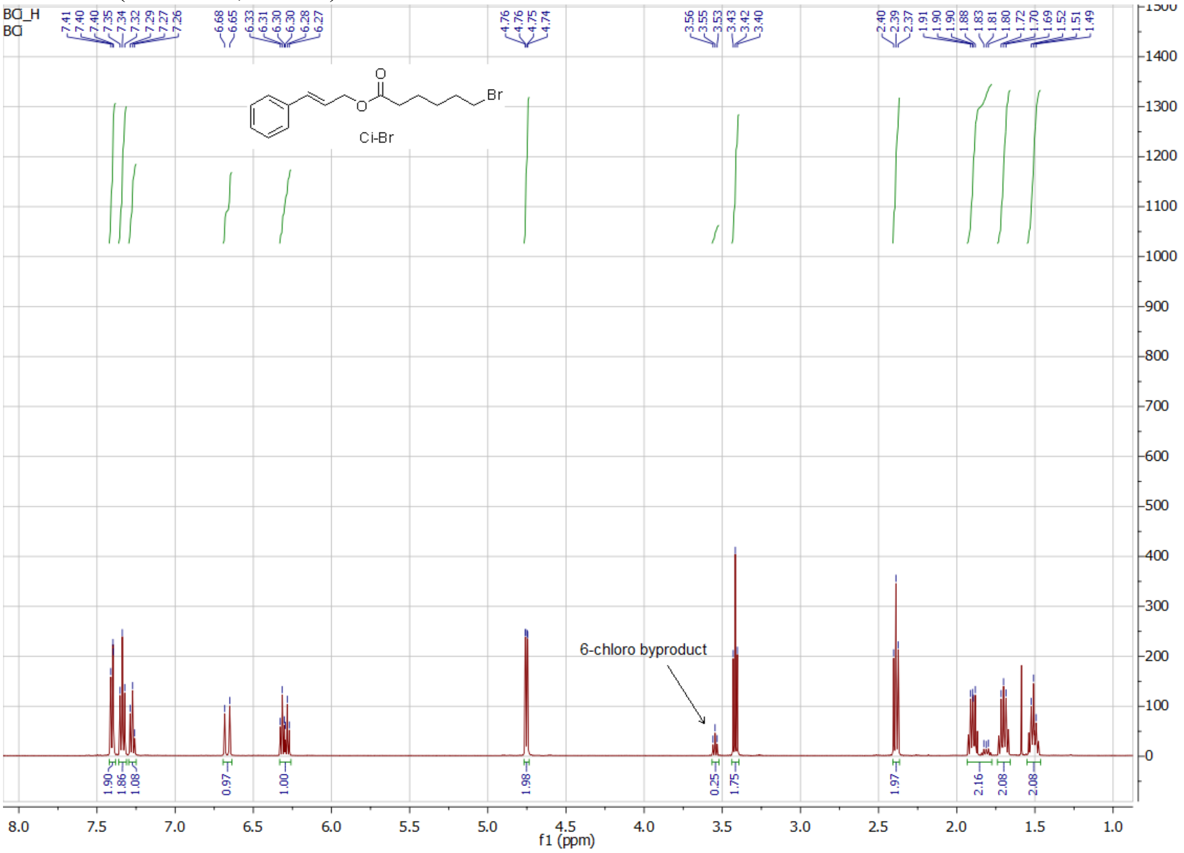

^{13}C NMR (126 MHz, $CDCl_3$):

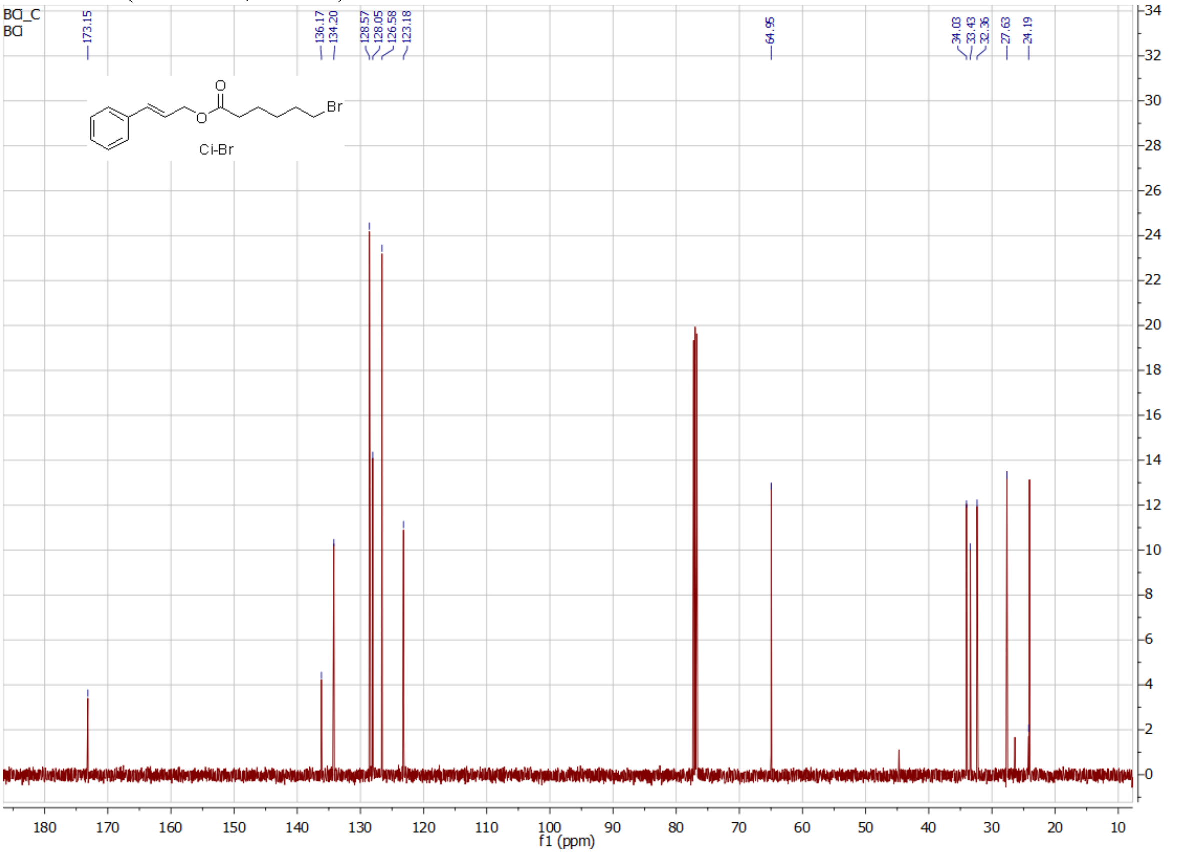

(*E*)-3-phenylprop-2-en-1-yl 6-(dimethylamino)hexanoate (**Ci-DAK**)

^{1}H NMR (300 MHz, $CDCl_3$):

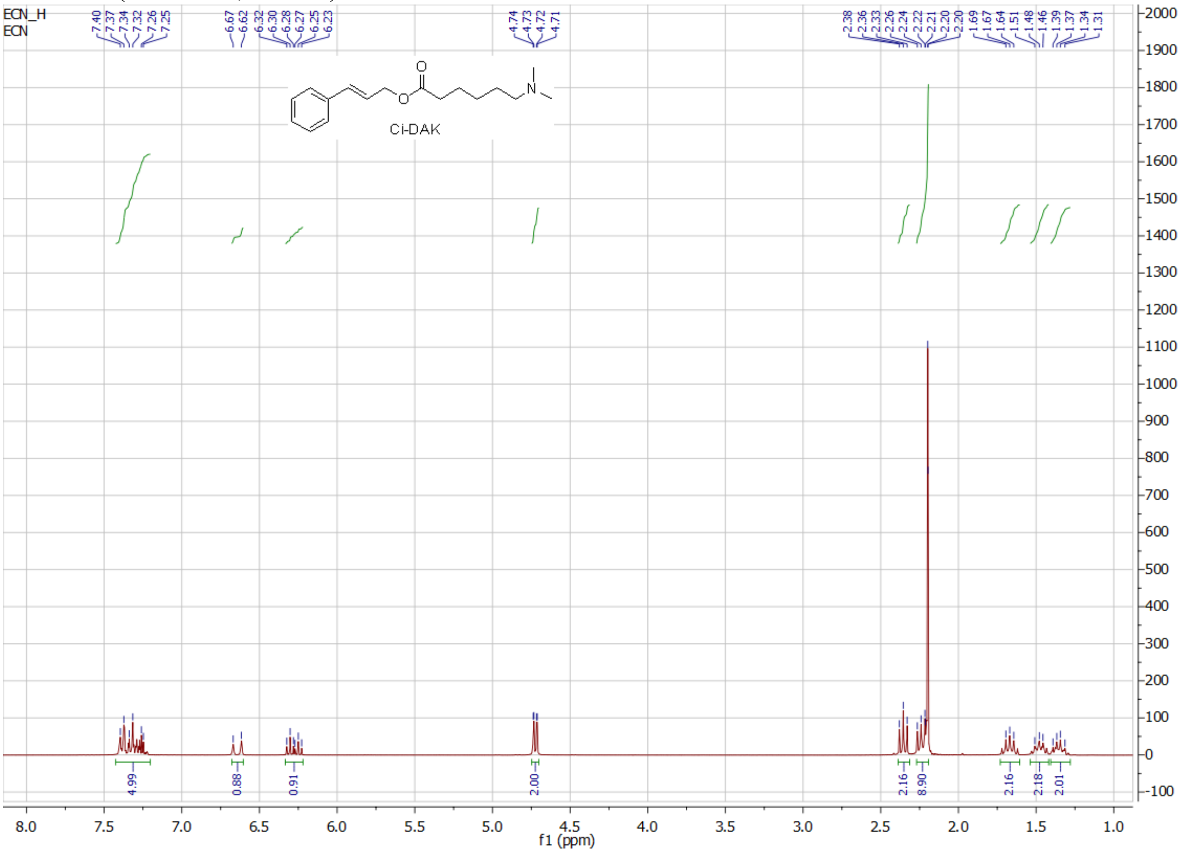

^{13}C NMR (75 MHz, $CDCl_3$):

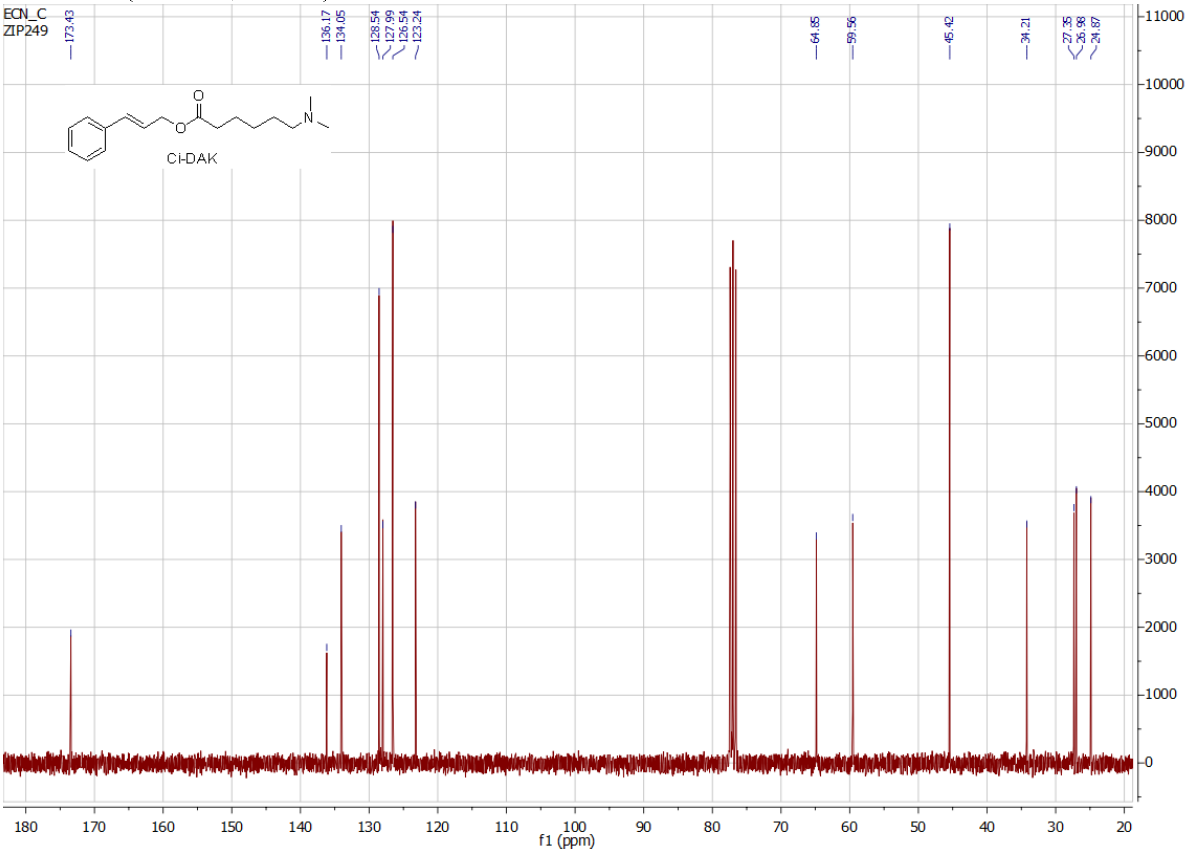

Supplement: Supplementary file 1 — Supplementary info [file 41598_2019_51226_MOESM1_ESM.pdf]
